# Recovery-oriented mental health training interventions: An integrative review

**DOI:** 10.1016/j.ijnsa.2026.100510

**Published:** 2026-02-15

**Authors:** Natthapon Inta, Mary Leamy, Annmarie Grealish

**Affiliations:** a Florence Nightingale Faculty of Nursing, Midwifery & Palliative Care, King’s College London, London, UK; b Princess Agrarajakumari Faculty of Nursing, Chulabhorn Royal Academy, Bangkok, Thailand; c School of Nursing and Midwifery, Health Research Institute, University of Limerick, Limerick, Ireland

**Keywords:** Mental health recovery, Recovery-oriented intervention, Educational training, Healthcare professionals, Peer support workers, Substance misuse

## Abstract

**Background:**

Whilst the concept of personal recovery in the mental health field, supporting people with mental illness in finding purpose and meaning in their lives, is gaining international recognition, many healthcare providers remain more familiar with clinical recovery, which focuses on medication and symptom management. In this review, we focused on personal recovery-oriented training interventions designed to improve recovery-related knowledge, attitudes, and competency among healthcare professionals and peer support workers caring for people with mental illness within mental health and substance misuse services. We have conceptualised key components and evaluated the effects of personal recovery-oriented training interventions to build a solid foundation of knowledge about these interventions, providing insight for enhancing recovery-oriented research and practices.

**Aims:**

To categorise recovery-oriented training interventions for healthcare professionals and peer support workers; to evaluate their effects on staff, service users, organisations; and to identify barriers and enablers influencing the implementation of recovery-oriented training programmes.

**Study design:**

An integrative review, registered to PROSPERO (CRD42023493803).

**Methods:**

We utilised eight databases (ASSIA, CINAHL, Medline, PsycINFO, Web of Science, Eric, TCI, and ThaiJo). The Template for Intervention Description and Replication (TIDieR) checklist and Kirkpatrick’s four level of training evaluation model were used in data extraction, then a narrative synthesis was used for the data synthesis review stages.

**Results:**

We analysed 33 studies and identified seven categories of recovery-oriented training interventions, based on their core attributes and underlying theories. Key components of the training included understanding recovery concepts, addressing staff and team attitudes, fostering supportive and collaborative relationships, and promoting patients' autonomy, hope, and motivation. Most interventions showed a positive impact on healthcare professionals' knowledge and attitudes towards recovery, as well as on patient recovery outcomes. However, a failure to establish effectiveness of recovery-oriented training interventions can also be attributed to inadequate implementation, rather than to the content of training. Barriers to effective implementation included organisational structure changes, workload, professional constraints (such as time limitations and philosophical opposition), and service users’ conditions.

**Conclusions:**

We suggest that recovery-oriented training interventions have the potential to improve recovery knowledge and attitudes among healthcare professionals and thus improve patients’ recovery outcomes. To address implementation challenges, the content and format of the training interventions should be tailored to the context and audience by adjusting the intensity and duration to better suit the needs of the participants or organisations.

**What is already known**

• Training and education programmes are a key strategy for improving practices, knowledge, and skills in health services.
• Training interventions focused on personal recovery in mental health have the potential to enhance the recovery-related knowledge, attitudes, and competency of healthcare professionals.

Alt-text: Unlabelled box dummy alt text

**What this paper adds**

• Seven categories of recovery-oriented training were identified.
• We suggest that training can improve staff knowledge and attitudes and enhance service users... recovery outcomes.
• Organisational structure changes and workload, professional barriers (i.e., time constraints, philosophical opposition, disengagement), and service user characteristics (i.e., severity of illness) are factors that impede the implementation of recovery-oriented training interventions.
• We found that the dominance of research from the United States, United Kingdom, and Australia and the lack of data on non-white participants highlight the need for cultural-adapted recovery interventions in under-researched populations, especially in low-to-middle-income countries, to maintain fidelity, engagement, and cultural competence.

Alt-text: Unlabelled box dummy alt text

## 1 Introduction

Traditionally, mental health service has been understood from a psychiatric perspective, often referred to as clinical recovery (Pilgrim and McCranie, 2013). This approach focuses on the reduction of symptoms, the resolution of mental disorders, and the eventual discontinuation of mental health services, with progress typically measured through clinical outcomes (Kuek et al., 2020, Topor et al., 2022). The concept of mental health recovery emerged in the late 1980s in Western countries, driven by a movement of people living with mental illness who sought to challenge assumptions about their ability to lead productive and fulfilling lives (Bellack, 2006, Fisher, 2003, Hawsawi et al., 2021). Personal recovery emerged to address the limitations of clinical recovery, which often overlooks individuals’ lived experiences and their aspirations for a meaningful life (Davidson et al., 2008). It emphasises supporting people with mental illness in finding purpose and meaning in their lives, even whilst living with ongoing symptoms (Anthony, 1993).

In recognition of this broader perspective, there has been a shift in mental health services, encouraged by government policies and the World Health Organization’s comprehensive health action plans (World Health Organization, 2021), towards placing greater emphasis on personal recovery in mental health rather than focusing solely on clinical outcomes. Although this underscores the significance and timeliness of recovery-oriented approaches in mental health services, not all countries have adopted a recovery-oriented approach, particularly in low- and middle-income countries (Gamieldien et al., 2021). However, personal recovery has been endorsed in many anglophone countries; for example in the United Kingdom (UK) (Department of Health, 2011), the United States of America (USA) (Substance Abuse and Mental Health Service Administration, 2005), Canada (Mental Health Commission of Canada, 2009), New Zealand (Ministry of Health, 2002, Ministry of Health, 2016), and Australia (National Mental Health Commission, 2017), with more recent developments also emerging in some Asian countries (Khanthavudh et al., 2023).

Recovery-oriented practices encompass various types of interventions, such as psychoeducation and peer support/peer-led programmes, and emphasise social inclusion and mental health literacy training (Winsper et al., 2020). Whilst many recovery interventions seem promising (Eiroa-Orosa and García-Mieres, 2019), often obstacles may prevent those interventions from being successful, such as unfamiliarity with personal recovery, and insufficient support for patients to engage with interventions successfully (Topor et al., 2022). Healthcare providers often lean towards a biomedical orientation in their care practices (Bee et al., 2015), and transforming services requires professionals to adopt recovery-oriented practice values, principles, attitudes, and behaviours to empower service users. Training and education programmes are considered key to improving practices, knowledge, expertise, abilities, and skills in healthcare organisations (Roberts and Boardman, 2014, Williams et al., 2016).

Previous reviews of recovery-oriented training interventions have identified their characteristics (Jackson-Blott et al., 2019), explored nurses’ learning experiences of recovery training (Hawsawi et al., 2021), and summarised their effectiveness (Eiroa-Orosa and García-Mieres, 2019). For example, a meta-analysis on the impact of recovery-oriented training on health professionals found that training activities moderately influenced their knowledge and attitudes, but the impact on practice was less well-established (Eiroa-Orosa and García-Mieres, 2019). However, no previous reviewers have expanded the population to explore training interventions for non-professional peer support workers nor broadened the context to include implementation in substance misuse services. Most importantly, for the purpose of implementation, adaption, and fidelity, previous reviewers have not provided a detailed understanding of the training components or sufficiently reported the content of recovery training interventions to strengthen our understanding of how they work to produce desired outcomes. In this integrative review, we focused on the content of training interventions using Template for Intervention Description and Replication (TIDieR) reporting checklist, their overall effects using Kirkpatrick’s training evaluation model, and the issues that influence the success of their implementation across a range of healthcare services.

## 2 Methods

An integrative review includes quantitative and qualitative research designs to fully understand the phenomenon of interest. The five-stage process of the integrative review set out by Whittemore and Knafl (2005) was applied: (1) problem identification, (2) literature search, (3) data evaluation, (4) data analysis, and (5) Interpretation and presentation of findings. The study was reported in line with the Preferred Reporting Items for Systematic review and Meta-Analyses (PRISMA) guidelines (Page et al., 2021), and the review protocol was registered with PROSPERO (CRD42023493803).

### 2.1 Search strategy

Searches were developed with three main concepts as per the PI(O) framework (Population-Intervention-Outcome) (Richardson et al., 1995): healthcare professionals/peer support workers in mental health/substance misuse service (Population) and recovery-oriented training intervention (Intervention). Boolean operators ‘OR’ and ‘AND’ were used to link search terms related to the three main concepts across six databases including ASSIA, CINAHL, Medline, PsycINFO, Web of Science, and Eric. Two Thai databases, Thai-Journal Citation Index (TCI) and ThaiJo, do not support Boolean operators; thus, hand search was employed. The searches were conducted in December 2023 and updated in February 2025. The full search strategy can be found in **Supplementary Material 1**. The search was limited to papers published after 1988 to ensure the inclusion of relevant studies but in recognition that the recovery concept is a relatively new development in the field of mental health (Farkas et al., 2005). The search was not restricted to any specific languages, and it was modified accordingly for each database. Open Grey, Google Scholar, and Google were used to investigate the grey literature to mitigate publication bias and ensure that pertinent unpublished literature was not overlooked. Direct forward and backward citation tracking was used to check the reference lists of the included papers.

### 2.2 Eligibility criteria

The SPIDER framework (Sample, Phenomenon of Interest, Design, Evaluation, and Research type) (Cooke et al., 2012) was used alongside the PIO framework to guide the eligibility criteria, as it enables the integration of primary research, including quantitative, qualitative, and mixed-methods research studies. Therefore, the framework is well-suited for integrative review purposes. Table 1 presents the inclusion and exclusion criteria.

**Table 1:** Inclusion and exclusion criteria.

	**Inclusion criteria**	**Exclusion criteria**
Sample (Population)	- Any studies that target recovery-oriented training programme for qualified healthcare professionals who work in health services (hospital or community) e.g., nurses, doctors, psychologists, social workers, or peer support workers who are part of the clinical team	- Any studies that do not feature healthcare professionals but include ONLY non-professionals (patients, family members, carers) or unqualified health workers (e.g., pre-registration students, support accommodation staff)
Phenomenon of Interest (Intervention)	- Recovery-oriented educational and training programmes for professional staff that target personal recovery from mental illness and substance-related mental illness for adults aged 18 and over	- Training focuses on clinical rather than personal recovery or non-mental illness or non-substance-related mental illness - Recovery colleges, learning centres that offer courses to help people improve their wellbeing by bringing together those with personal experience of mental health challenges and professionals as equal partners in learning - Training that is not a stand-alone recovery-oriented programme or is just a part of another training programme; e.g., cognitive behaviour therapy, professional training, aggression management training or similar - Intra-curricular clinical placement training for pre-registration professionals
Design	- Published articles of any empirical research design; e.g., effectiveness study, implementation study, feasibility/acceptability study - Published literature reviews, meta-analyses, and umbrella reviews - Grey literature and training manuals - Published in English and Thai	- Non peer reviewed sources; e.g., protocol, letters, dissertations/unpublished thesis, book chapters
Evaluation (Outcomes)	- All outcomes examined by the recovery-oriented educational and training programmes at any levels (staff, service users, institutional outcomes)	None
Research type	- All study types reporting or evaluating recovery-oriented training programmes e.g., quantitative, qualitative, mixed-methods, literature review	None

### 2.3 Study selection

All retrieved studies were imported to Covidence (www.covidence.org), and duplicates were removed. NI examined the title and abstract and evaluated full text for eligibility requirements. Any ambiguities or uncertainties were resolved through discussion with ML and AG.

### 2.4 Quality assessment

The final set of included articles were critically evaluated to investigate their quality, reliability, and importance (Buccheri and Sharifi, 2017). The Mixed Methods Appraisal Tool, version 2018, was used to appraise mixed-methods studies (Hong et al., 2018). The revised Cochrane risk-of-bias tool for randomised trials (RoB2), was used to assess risk of bias (Sterne et al., 2019). JBI’s critical appraisal tools were used to evaluate qualitative (Lockwood et al., 2015), quasi-experimental (Tufanaru et al., 2020), cross-sectional (Moola et al., 2020), and review studies (Aromataris et al., 2015). Due to lack of a formal risk of bias evaluation tool for grey literature, eligibility criteria were based upon whether they provided information that was sufficiently relevant and rigorous to answer our research questions (Adams et al., 2016).

The critical appraisal of all 33 included papers was independently conducted by NI, whilst ML and AG each independently assessed half of the included papers, with any disagreements discussed among authors. Instead of computing an overall score, quality ratings were evaluated across all study criteria as recommended by each critical appraisal tool guideline. We did not omit any study based on the quality assessment rating; however, ratings were taken into consideration during data analysis and synthesis.

### 2.5 Data extraction

The initial data extraction was conducted by NI, whilst ML and AG reviewed data accuracy. The data extraction table included columns to describe each of the key study characteristics, namely: author and year, country, study design, aims, participant characteristics, main findings, intervention characteristics, outcome measures, and barriers and facilitators to implementing the intervention.

The TIDieR checklist was used to map key recovery-oriented training intervention characteristics (Hoffmann et al., 2014). The characteristics of each recovery-oriented training programme were described as follows: foundation theory (why), material and procedure used in the intervention (what), providers of interventions (who), mode of delivery (how), place to deliver interventions (where), intervention frequency and duration (when and how much), intervention specificity (tailoring), whether the intervention was modified (modifications), and intervention adherence (how well). In addition, Kirkpatrick's four levels of a training evaluation model (Kirkpatrick and Craig, 1970), which demonstrated face and content validity, support its effectiveness in capturing the impact of training on both individual and organisational outcomes (Bahl et al., 2024), was used to guide our data extraction and to group findings into the four categories: trainee’s reactions, trainee’s learning outcomes, trainee’s behaviour changes, and patient outcomes (Kirkpatrick and Kirkpatrick, 2016), to support a more comprehensive and balanced evaluation of the overall effects of the training interventions.

### 2.6 Data analysis

Given the substantial methodological heterogeneity was identified among the studies, narrative synthesis techniques (textual description of studies, forming appropriate groupings and clusters, tabulation, and translating data) guided by Popay et al. (2006) were used.

The narrative synthesis was guided by the research questions. For research question 1, the key characteristics of the interventions were tabulated according to the TIDieR checklist, and these key characteristics were then used to categorise the interventions. For research question 2, the extracted data were then synthesised to explore the effects of the identified training interventions through the lens of the four categories of Kirkpatric's model. For research question 3, barriers and enablers were used to synthesise the factors influencing the implementation of the interventions.

## 3 Results

### 3.1 Search results

**Fig. 1:**
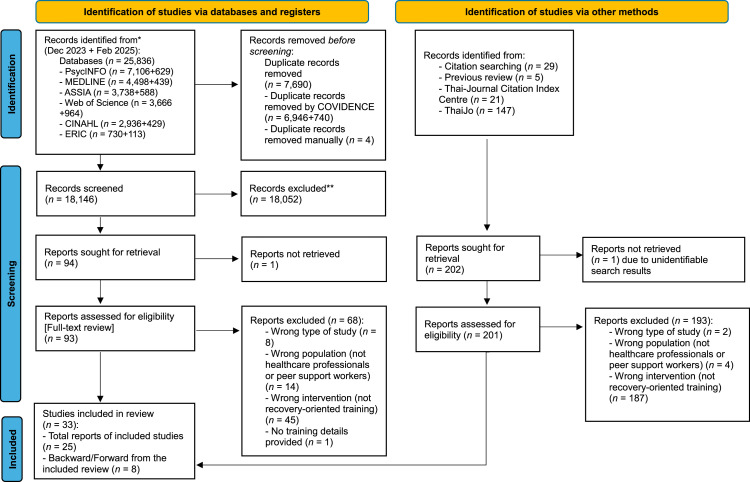
PRISMA flow diagram of study selection process.

The database search yielded a total of 25,836 articles and included 33 studies in the review. A summary of the systematic search strategy and screening assessment of eligibility criteria is summarised in Fig. 1.

### 3.2 Study characteristics

Study characteristics are summarised in Table 2. The studies were published between 2005 to 2023 and conducted across 10 countries and one Special Administrative Region: UK (*n*=7), Australia (*n*=12), USA (*n*=5), Netherlands (*n*=2), and one each in Ireland, Switzerland, Italy, Spain, Israel, Japan, and Hong Kong. Seventeen were quantitative in design, comprising randomised control trials (*n*=3); quasi-experimental studies (*n*=11); and cross-sectional studies (*n*=3). Six used a qualitative design, using data from interviews (*n*=5), focus groups (*n*=3), and observation/field notes (*n*=1). Other designs included mixed methods (*n*=5) and literature reviews (*n*=3).

**Table 2:** Characteristics of included studies according to study design, ordered alphabetically (*N*=33).

**Author (year)/ Country**	**Aim/objectives**	**Study design** **/** **Intervention**	**Participants’ characteristics**	**Outcome measures**	**Outcomes**	**Main findings**
**Randomised control trial (RCT) design**						
Mak et al. (2019) Hong Kong	To develop a culturally responsive and contextually appropriate brief psychoeducation program for mental health service providers and users in Hong Kong. To evaluate its efficacy in promoting the knowledge of and attitudes towards recovery.	RCT *- Brief Recovery Psychoeducation Program for Service Providers* *- Brief Recovery Psychoeducation Program for Service users*	**Study 1: HCPs aspect** ***Sample size:****n*=111 ***Participants characteristics:*** mental health service providers F (*n*=71, 65.4 %), M (*n*=40, 34.6 %), Mean age of 34.86 years (S.D. = 8.29 years, range 22–54), Social workers, nurses, and occupational therapists (57.7 %, *n*=64), programme workers, care assistants, and health workers) (42.3 %, *n*=47) ***Clinical/Recovery experience***: 7.05 years (S.D. = 6.13 years). **Study 2: Patients aspect** ***Sample size:****n*=93 ***Participants characteristics:*** people with mental illness Mean age of 41.02 ± 11.14 years (range 19–69), Secondary education as the highest level of education (71.4 %, *n*=65), Diagnosed with schizophrenia (*n*=50), dual diagnosis (*n*=3), major depressive disorder (*n*=18), bipolar disorder (*n*=9)	- The Recovery Knowledge Inventory (RKI) - The Attitudes towards Recovery Questionnaire (ARQ)	***Primary outcomes:*** **Recovery knowledge** RKI (Chinese version) (α 0.78–0.88) - Baseline mean score: I: 2.78 [SE 0.05], C: 2.77 [0.05] - Post-programme mean score: I: 2.98 [0.05], C: 2.76 [0.06] - One-month follow up mean score: I: 2.83 [0.05], C: 2.74 [0.06] **Recovery attitudes** 2. ARQ (Chinese version) (α 0.89–0.90) - Baseline mean score: I: 4.06 [0.08] C: 4.20 [0.09] - Post-programme mean score: I: 4.05 [0.09] C: 3.91 [4.01] - One-month follow up mean score: I: 4.10 [0.09] C: 4.01 [0.09]	Participants in psychoeducation group had significantly better recovery knowledge and more positive attitudes towards recovery after the intervention than the control counterparts. The effect of the recovery psychoeducation programme on recovery attitudes was fully mediated by the improvement in recovery knowledge.
Meadows et al. (2019) Australia	To establish whether individuals who access mental health services where staff have received the REFOCUS-PULSAR intervention, an adaptation of the UK’s REFOCUS recovery-oriented staff intervention for use in Australia, show increased recovery compared with people using non-intervention services.	RCT (step-wedge) *- REFOCUS-PULSAR*	***Sample size:*** HCPs: *n*=190 Patients: *n*=942 (T0: *n*=301, T1: *n*=334, T2: *n*=307) ***Participants characteristics:*** HCPs: Staff were working part-time or full-time in a direct service role at one of the 18 sites and had consumers. Patients: Patients were receiving care from a participating cluster, with contact in the 3 months before data collection; aged 18–75 years; and not imprisoned. Male: *n*=390 (41 %); Female: *n*=554 (58 %) Age of 17–29 years: *n*=229 (24 %) Age of 30–49 years: *n*=472 (50 %) Age of ≥ 50 years: *n*=230 (24 %) Non-indigenous Australian: *n*=460 (49 %) Indigenous Australian: *n*=80 (8 %) Other ethnicities: *n*=343 (36 %) ***Clinical/Recovery experience:*** N/R	- The Questionnaire about Processes of Recovery (QPR) - INSPIRE - Warwick-Edinburgh Mental Well-Being Scale (WEMWBS) - The Perceived Need for Care Questionnaire for assessing patients’ perceptions of mental health care and classifies perceived needs - The Client Satisfaction Questionnaire - The Coercion Ladder - The Global Assessment of Functioning (GAF) - The Social and Occupational Functioning Assessment Scale for measuring function, independent of psychological condition severity - The Social and Occupational Functioning Assessment Scale - Days out of role	***Primary outcomes:*** **Patient’s personal recovery** 1. QPR (I: 54·4 [SD 16·2], C: 53·6 [16·3]) ***Secondary outcomes:*** **Recovery support** 1. INSPIRE (Support: I: 62.2 [23.1], C: 62.4 [22.3]) (Relationship: I: 75.5 [20.1], C: 72.0 [22.3]) **Emotional and functional wellbeing** 2. WEMWBS (I: 42.2 [11.1], C: 41.1 [11.2]) **Patients’ satisfaction with services** 3. The Client Satisfaction Questionnaire (I: 24.5 [5.5], C 23.3 [5.3]) 4. The Mind Australia Satisfaction Survey (I: 8.2 [1.8], C: 8.0 [1.8]) **Patients’ perception of coercion in mental health service interactions** 5. The Coercion Ladder (I: 2.0 [1.5], C: 2.0 [1.5]) **Individual’s social, occupational, and psychological functioning** 6. GAF (I: 51.4 [13.3], C: 48.5 [14.7]) **Function, independent of psychological condition severity** 7. The Social and Occupational Functioning Assessment Scale (I 52.9 [14.3], C: 49.8 [15.5]) **The effect of mental health problems on usual daily activities over the past 30 days** 8. Days out of role (I: 6.0 [0.0 – 15.0], C: 6.5 [0.0 – 15.0])	The mean Process of Recovery (QPR) score was 53·6 (SD 16·3) in the control group and 54·4 (16·2) in the intervention group (adjusted difference 3∙7, 95 % CI 0·5–6·8; *p*=0·023). The Cohen’s d value for the intervention effect was small (*d*=0·23).
Slade et al. (2015a) UK	To test the hypothesis that the REFOCUS intervention would be associated with improved recovery compared with usual care.	RCT *- REFOCUS*	***Sample size:*** HCPs: I: *n*=14 teams, C: *n*=13 teams Patients: *n*=403 (I: 210, C: 193) ***Participants characteristics:*** *HCPs:* Multidisciplinary mental health teams in Community-based adult mental health (SlaM in London and 2gether in Gloucestershire) *Patients:* Male (I: *n*=131; 63 %, C: *n*=127; 66 %) Female (I: *n*=78; 37 %, C: *n*=66; 34 %) White (I: *n*=115; 56 %, C: *n*=95; 49 %) Non-white (I: *n*=92; 44 %, C: *n*=98; 88 %) Mean age (I: 44.8 years, C: 42.99 years) Mean of using service (I: 16.3 years, C: 15.52 years) ***Clinical/Recovery experience:*** N/R	- The Questionnaire about Processes of Recovery (QPR) - Manchester Short Assessment of Quality of Life (MANSA) - Mental Health Confidence Scale (MHCS) - Warwick-Edinburgh Mental Well-Being Scale (WEMWBS) - Camberwell Assessment of Needs Short Appraisal Schedule-Patient (CANSAS-P) - The Client Satisfaction Questionnaire (CSQ) - INSPIRE - CANSAS- Staff (CANSAS-S) - The Global Assessment of Functioning (GAF) - The Health of the Nation Outcome Scale (HoNOS) - The Recovery Knowledge Inventory (RKI) - Clinicians’ Attitudes (MICA) questionnaire - The Recovery Practice Scale	***Primary outcomes:*** **Patient’s personal recovery** 1. QPR - Baseline mean score (I: 38.5 [SD 9.8], C: 38.6 [SD 9.5]) - 1 year follow up mean score: (I: 40.6 [10.1], C: 40.2 [10.3]) ***Secondary outcomes:*** **Hope, quality of life, empowerment, wellbeing, and met and unmet needs in patients:** 1. MANSA (I: 4.88, C: 4.80; *p*=0.49), MHCS (I: 67.81, C: 67.06; *p*=0.64), WEMWBS (I: 48.09, C: 470.24; *p*=0.37), CANSAS-P (Met: I: 4.41, C: 4.13; *p*=0.54) (Unmet I: 3.69, C: 3.88; *p*=0.59). **Patient’s experience of satisfaction** 2. CSQ (I: 25.99, C: 25.31; *p*=0.16) **Recovery support** 3. INSPIRE (relationship: I: 78.34, C: 78.67; *p*=0.85) (Support: I: 61.53, I: 64.57; *p*=0.22) **Patients met and unmet needs** 4. CANSAS-S (Met: I: 5.79, C: 5.73; *p*=0.92) (Unmet: I: 2.26, C: 3.13; *p*=0.03, *d*=0.32) **Functioning** 5. GAF (I: 67.97, C: 31.84; *p*<0.001, *d*=0.41) **Social disability** 6. HoNOS (I: 9.17, C: 10.50; *p*=0.05) **Staff’s recovery-related knowledge and attitudes** 7. RKI (I: 2.95, C: 2.86) 8. MICA (I: 30.38, C: 30.48) **Self-rated skills** 9. The Recovery Practice Scale (I: 2.84, C: 2.91) 10. Behavioural intent (I: 1.62, C: 1.68)	No significant effect of the REFOCUS intervention on recovery in patients with psychosis compared with usual treatment. Mean QPR total scores did not differ between the two groups (REFOCUS group 40·6 [SD 10·1] vs control 40·0 [10·2], adjusted difference 0·68, 95 % CI –1·7 to 3·1, *p*=0·58).
**Quasi-experimental design**						
Deane et al. (2014) Australia	To compare the effect of two coaching conditions, skills-based and transformational coaching, on the implementation of a recovery-oriented model following training.	Quasi-experimental study *- Skills acquisition coaching and Transformational coaching*	***Sample size:****n*=91 ***Participants characteristics:*** Staff of four community-managed mental health organisations, representing 13 sites across four states of Australia Gender: M = 23, F = 60, Missing = 8 Clinical role: Case manager = 77 Peer support = 8 Missing = 6 ***Clinical/Recovery experience:*** - <1–2 years (*n*=27) - 2–5 years (*n*=24) - 5–10 years (*n*=15) - 10->15 years (*n*=9) - Missing (*n*=16)	- The Goal and Action Plan Instrument for Quality (GAP-IQ)	***Primary outcomes:*** **The quality of clinical goal setting and action planning** 1. GAP-IQ - Pre-intervention mean sore: Transformational 14.54 (SD 1.26); Skills 11.81 (1.39) - 6-month follow up mean sore: Transformational 16.48 (1.13); Skills 14.75 (1.19) - 12-month follow up mean score: Transformational 18.48 (1.23); Skills 14.74 (1.17)	Two days of the CRM training followed by coaching led to significant sustained improvements in the quality-of-care planning in accordance with the new model over the 12-month study period. However, no significant difference between the coaching conditions in the number of Coaching Record Sheets returned (*p* > 0.05).
Young et al. (2005) USA	To evaluate the effectiveness of an innovative, consumer-led intervention, Staff Supporting Skills for Self- Help, which was designed to improve provider quality, empower mental health consumers, and promote mutual support.	Quasi-experimental study *- Consumer-led intervention, Staff Supporting Skills for Self-Help*	***Sample size:*** I: *n*=151, C: *n*=118 ***Participants characteristics:*** Clinical staff providing care to people with severe and persistent mental illness; Ethnicity: White (I: *n*=95, C: *n*=83); Hispanic (I: *n*=28, C: *n*=10); African American (I: *n*=13, C: *n*=11); American Indian (I: *n*=5, C: *n*=2); F (I: *n*=102, C: *n*=69) Gender: M (I: *n*=46, C: *n*=44) F (I: *n*=102, C: *n*=69) ***Clinical/Recovery experience:*** Mean ± SD = 8.5 ± 7.9	- The Competency Assessment Instrument (CAI) consisting of General competencies, Assessment and treatment competencies, and Rehabilitation competencies Holistic approach.	***Primary outcomes:*** **Clinician’s competencies, care processes, and the formation of mutual support** 1. CAI - Overall competency (*r*=0.21, *p*=0.02) - Teamwork (*r*=0.28, *p*=0.003) - Holistic approach (*r*=0.17, *p*=0.06) - Education about care (*r*=0.22, *p*=0.03) - Rehabilitation methods (*r*=0.25, *p*=0.007) - Natural supports (*r*=0.24, *p*=0.02)	Compared with clinicians at the control organisations, clinicians at intervention organisations showed significantly greater improvement in education about care, rehabilitation methods, natural supports, holistic approaches, teamwork, overall competency, and recovery orientation.
Zuaboni et al. (2017) Switzerland	To assess the effect of an intervention utilising a recovery-oriented approach in acute practice.	Quasi-experimental study *- Recovery-oriented nursing training programme*	***Sample size:*** Intervention group: Patients (*n*=73) MHNs (*n*=43) Control group: Patients (*n*=29) MHNs (*n*=19) ***Participants characteristics:*** Intervention group: Mean age of the MHNs = 38.1 years (SD = 9.4), F = 52.4 % Control group: Mean age of the MHNs = 40.6 years (SD = 8.0), F = 44.4 % ***Clinical/Recovery experience:*** Intervention group: Mean length of work experience = 12.6 years (SD = 7.2) Control group: mean length of work experience = 14.8 years (SD = 8.4)	- The German version of the Recovery Self- Assessment Scale (RSA-D)	***Primary outcomes:*** **Perceptions of the degree to which mental health services implement recovery-oriented practices.** 1. RSA-D - Baseline mean score: I (3.86 [SD 0.72]), C (3.67 [0.68]) - Post-intervention mean sore: I (3.86 [0.78]), C (3.83 [0.52])	No statistically significant effects were found, between the intervention ward and control ward in regard to both patients and MHNs outcomes (*p*=0.319). Reasons for this result may be methodological, practical, or due an intervention that was not powerful enough. Recovery-oriented intervention studies in mental health nursing should be conducted with caution.
**Pre-test/post-test design**						
Crowe et al. (2006) Australia	To examine the impact of a recovery-based training program for mental health workers on knowledge, attitudes, and hopefulness related to the recovery prospects of people with enduring mental illness.	Pretest/posttest design *- Collaborative Recovery* *Training Programme (CRTP)*	***Sample size:*** MHW from government (*n*=147) MHW from non-government (*n*=101) ***Participants characteristics:*** Mental health workers from the community-based government health sector Female: *n*=174 (70 %) Age: 40.91±9.92 years ***Clinical/Recovery experience:*** 12.01±9.87 years	- The Recovery Attitudes Questionnaire (RAQ-7) (α 0.64–0.70) - The staff attitudes to recovery scale (STARS) (α 0.81) - The collaborative recovery knowledge scale	***Primary outcomes:*** **Recovery attitudes** 1. RAQ-7 First factor: - Pre-training mean score: Government (16.01 [SD 2.26]); Nongovernment (16.57 [2.18]) - Post-training mean score: Government (17.05 [2.19, Effect size 0.17^⁎⁎^); Nongovernment (17.11 [3.34, 0.02]) Second factor: - Pre-training mean score: Government (13.02 [1.50]); Nongovernment (13.55 [1.18]) - Post-training mean score: Government (13.32 [1.51, 0.03*); Nongovernment (13.22 [2.39, 0.02]) **Attitudes and hopefulness related to the goal striving and recovery possibilities for the consumers** 2. STARS - Pre-training mean score: Government (67.96 [8.07]); Nongovernment (66.83 [9.87]) - Post-training mean score: Government (73.80 [7.44, 0.48^⁎⁎^]); Nongovernment (71.01 [9.86, 0.38^⁎⁎^]) **Staff recovery-related knowledge** 3. The collaborative recovery knowledge scale - Pre-training mean score: Government (7.54 [1.99); Nongovernment (7.32 [1.98]) - Post-training mean score: Government (9.39 [1.72, 0.52^⁎⁎^]; Nongovernment (8.69 [2.33, 0.25^⁎⁎^])	Staff attitudes and hopefulness improved after training. Trainees significantly increased their knowledge regarding principles of recovery and belief in the effectiveness of collaboration and consumer autonomy support, motivation enhancement, needs assessment, goal striving, and homework use.
Enticott et al. (2021) Australia	To examined whether interventions promoting recovery-oriented practice in general practice settings can improve outcomes for patients.	Pretest/posttest design *- REFOCUS-PULSAR*	***Sample size:*** GP (*n*=23), Patient surveys (*n*=235) ***Participants characteristics:*** ***-*** Primary care site (clusters) *n*=18 (17 privately owned general practices and 1 community health centre) - Patient survey: ***Clinical/Recovery experience:*** N/R	- The Process of Recovery (QPR) - The INSPIRE questionnaire - The Warwick- Edinburgh Mental Well-being Scale (WEMWBS) - The Kessler Psychological Distress Scale (K10)	***Primary outcomes:*** **Patient’s personal recovery** 1. QPR - Pre-intervention mean score: 52.8 (SD 15.6) - Post-intervention mean score: 57.3 (14.3), *p*=0.01, d = 0.29 ***Secondary outcomes:*** **Recovery support** 1. The INSPIRE-S questionnaire - Pre-intervention mean score: 68.8 (22.2) - Post-intervention mean score: 65.9 (24.6), *p* = 0.39 2. The INSPIRE-R questionnaire - Pre-intervention mean score: 81.9 (18.4) - Post-intervention mean score: 82.7 (16.6), *p* = 0.36 **Emotional and functional wellbeing** 3. WEMWBS - Pre-intervention mean score: 40.0 (11.4) - Post-intervention mean score: 43.1 (10.8), *p*=0.02, *d*=0.28 **Distress based on questions about anxiety and depressive symptoms** 4. K10 - Pre-intervention mean score: 29.3 (8.9) - Post-intervention mean score: 26.6 (8.0), *p* = 0.01, *d*=0.32	Small positive significant effects indicated primary-outcome post-intervention improvements [*t*-test (233) = −2.23, *p* = 0.01], also improvement in two secondary outcomes (WEMWBS *t*(233) = −2.12, *p* = 0.02 and K10 *t*(233) = 2.44, *p* = 0.01).
Giusti et al. (2022) Italy	To assess the effectiveness of our short Personal Recovery Training Program (PRTP) for mental health professionals compared to the Family Psychoeducational Training Program (FPTP).	Pretest/posttest design (with comparison group) *- Personal Recovery Training Program (PRTP)* *- Family Psychoeducational Training Program (FPTP)*	***Sample size***: Healthcare professional (*n*=52) + Students in psychiatric rehabilitation (*n*=40) **PRTP** ***Participants characteristics:*** M (*n*=10), F (*n*=35) Age (mean = 30) Psychiatrists (*n*=11), RN (*n*=3), Psychologist (*n*=2), Psychiatric rehabilitation technicians (*n*=9), Student (*n*=20) **FPTP** ***Participants characteristics:*** M (*n*=7), F (*n*=40) Age (mean = 32.1) Psychologist (*n*=4), RN (*n*=5), Psychologist (*n*=4), Psychiatric rehabilitation technicians (*n*=14), Student (*n*=20)	- The Recovery Knowledge Inventory (RKI) in Italian version	***Primary outcomes:*** **Recovery knowledge** 1. RKI (Italian version) *PRTP intervention:* - Pre-training mean score: 3.35 [SD 0.35] - Post-training mean score: 3.74 [0.34] *FPTPintervention:* - Pre-training mean score: 3.16 [0.41] - Post-training mean score: 3.33 [0.37]	Participants’ understanding of personal recovery improved more significantly for those in the PRTP than for those in the FPTP group in two domains, “Roles and responsibilities” and “non-linearity of the recovery process”; the FPTP group showed a significant improvement in the “Role of self-definition and peers in recovery” domain. RKI total score, increased significantly more among those undertaking the PRTP than among those in the FPTP group [F (1,90), = 7.39; *p* < 0.001].
Salgado et al. (2010) Australia	To determine whether attitudinal improvements following formal recovery training vary depending on participants’ dispositional hope.	Pretest/posttest design: non-control group *- Collaborative Recovery* *Training Programme (CRTP)*	***Sample size:****n*=103 ***Participants characteristics:*** Mental health workers (government and non-government organisations) Nurse (*n*=14, 16.3 %) Social worker (*n*=13, 15.1 %) Psychologist (*n*=8, 9.3 %) Occupational therapist (*n*=7, 8.1 %) Welfare worker (*n*=5, 5.8 %) ***Clinical/Recovery experience:*** N/R	- The Recovery Attitudes Questionnaire (RAQ) - The Staff Attitudes to Recovery Scale (STARS) - The Recovery Knowledge Inventory (RKI) - The Therapeutic Optimism Scale (TOS)	***Primary outcomes:*** **Recovery attitudes** 1. RAQ - Pre-training mean score: 4.29 [SD 0.40, Effect size 0.28] - Post-training mean score: 4.41 [0.45, 0.28] **Hopeful attitudes regarding consumers’ recovery possibilities** 2. STARS - Pre-training mean score: 3.95 [0.41, 0.87] - Post-training mean score: 4.28 [0.35, 0.87] ***Secondary outcomes:*** **Staff recovery attitudes and knowledge** 1. RKI - Pre-training mean score: 3.51 [0.39, 0.41] - Post-training mean score: 3.69 [0.49, 0.41] **General and personal treatment expectancies** 2. TOS - Pre-training mean score: 3.98 [0.37, 0.78] - Post-training mean score: 4.26 [0.35, 0.78]	Training improved providers’ recovery knowledge, attitudes, hopefulness, and optimism. Providers with both high and low dispositional hope (dividing pre-training HS scores at the 50th percentile) achieved similar gains.
Williamson et al. (2023) Australia	To evaluate the impact of a staff development training program informed by the collaborative recovery model (CRM) on staff outcomes in the largest implementation of CRM undertaken by a public clinical mental health service.	Pretest/posttest design *- The CRM staff development program*	***Sample size:****n*=729 ***Participants characteristics:*** Mental health workforce Medical Nursing Allied health Lived experience Leadership staff ***Clinical/Recovery experience:N/R***	- Adapted measure of Crowe et al. (2006): Attitudes and skills (α 0.82 and 0.83), Knowledge was measured by using 10 true-or-false items (score range 0–10, with higher scores indicating greater knowledge)	***Primary outcomes:*** *Attitude:* - Pre-training median and IQR: 44 (41–47) - Post-training median and IQR: 47 (44–50) - Booster training median and IQR: 47 (43–49) χ2=13.5, *p*=0.001 *Skills:* - Pre-training median and IQR: 29 (27–31) - Post-training median and IQR: 31 (28–33) - Booster training median and IQR: 29 (27–31) χ2=5.32, *p*=0.070 *Knowledge:* - Pre-training median and IQR: 8 (7–8) - Post-training median and IQR: 8 (7–9) - Booster training median and IQR: 8 (6–9) χ2= 2.27, *p*=0.648	The staff development program significantly (*p*<0.001) improved self-rated knowledge, attitudes, and skills in applying CRM. At booster training, improvements in attitudes and self- confidence in implementing CRM were maintained. Ratings of the importance of CRM and confidence in the organization’s implementation did not change. Definitions of recovery illustrated development of shared language throughout the large mental health program.
**Longitudinal design**						
Walsh et al. (2017) Ireland	To describe the effects of recovery-based training on staff knowledge and attitudes to recovery.	Longitudinal study (before training and 6 months after training) *- Recovery-based training*	***Sample size:****n*=101 ***Participants characteristics:*** Mental health staff employed in two regional health care areas who participated in a mandatory training programme that focused on a recovery-oriented approach to mental health care ***Clinical/Recovery experience:*** - 1–5 years (*n*=26, 26.8 %) - 6–10 years (*n*=15, 15.46 % - 11–15 years (*n*=16, 16.1 %) - 16.20 years (*n*=12, 12.37 %) - 21.25 years (*n*=8, 8.25 %) - 26–30 years (*n*=8, 8.25) - 30+ years (*n*=12,12.37 %)	- The Recovery Knowledge Inventory (RKI-20) (α 0.84) - The Recovery Attitudes’ Questionnaire (RAQ-16) (α 0.95)	***Primary outcomes:*** **Recovery knowledge** 1. RKI-20: All domains showed significant changes between pre- and post-training scores (*p* < 0.01). **Recovery attitudes** 2. RAQ-16: Both (factor 1 and 2) factors showed significant changes between pre- and post- training scores in Factor 1 (*p* < 0.001) and in Factor 2 (*p* < 0.009).	Recovery-based training positively affected staff knowledge and attitudes to recovery. However, because the organisational culture played a role in determining if recovery training changed practice, pre-training sessions with staff to identify any potential barriers were considered by those who embarked on delivering recovery training for staff.
G.K. Wilrycx et al. (2012) Netherlands	To investigate the effectiveness of a recovery-oriented training program on knowledge and attitudes of mental health care professionals towards recovery of people with serious mental illness.	Longitudinal study: stepped wedge *- Recovery-oriented training program*	***Sample size:*** - Intervention A (April 2008): *n*=162 - Intervention B (2009) *n*=96 ***Participants characteristics:*** Mental healthcare workers of the department “Impact” (the department for long-term mentally ill people in Breda/Etten-Leur) including - Psychologists/Psychiatrists (*n*=6) - Psychiatric nurse (*n*=117) - Occupational therapist (*n*=32) - Placement supporter (*n*=11) - Case manager (*n*=10) - Care assistant (*n*=10) - General staff members of Impact (n=24) ***Clinical/Recovery experience:*** Mean working history 43.3 (SD 10.2)	- The Recovery Attitude Questionnaire (RAQ) (the Dutch versions) (α 0.61) - The Recovery Knowledge Inventory (RKI) (the Dutch versions) (α 0.80)	***Primary outcomes:*** **Recovery attitudes** 1. RAQ (Dutch versions): χ2 = 0.890 with 3 degrees of freedom (*p* = 0.828). **Recovery knowledge** 2. RKI (Dutch versions): χ2 = 1.641 with 3 degrees of freedom (*p*=0.650).	Professionals’ attitudes towards recovery from mental illness improved with training. After two intensive recovery-oriented training sessions, mental health care professionals had a more positive attitude towards recovery in clinical practice.
Wilrycx et al. (2015) Netherlands	To examine the effects of a recovery-oriented care training program for mental healthcare professionals on mental health consumer outcomes.	Longitudinal study: stepped wedge *- Recovery-oriented care training program*	***Sample size:****n*=142 ***Participants characteristics:*** Consumers with severe mental illness: - Schizophrenia, psychotic disorders - Mood disorders - Anxiety disorders - Substance-related disorder - Other (including ADHD and ASD) ***Clinical/Recovery experience:*** N/R	- The Mental Health Recovery Measure (MHRM) (The Dutch version) (α 0.86–0.94) - The Recovery-Promoting Relationship Scale (RPRS) (The Dutch version) (α 0.87–0.93)	***Primary outcomes:*** **Recovery process of persons with severe mental illness** 1. MHRM (Dutch version): scores showed a significant change over time for the subscale ‘Learning & new potentials’ **Generic components of mental health providers’ recovery-promoting professional competence** 2. RPRS (Dutch version): showed no significant change over time	Patients with serious mental illness were able to make a start with their recovery process while professionals were being trained in the recovery vision. However, the results also showed that the relationship with the professional was not experienced as a more recovery-oriented one during and after the recovery-oriented training programme for professionals.
**Cross-sectional design**						
Tsai et al. (2010) USA	To examine the types of recovery-oriented trainings that occurred at two state hospitals over one year and subsequent changes in staff recovery attitudes.	Cross-sectional study *- General/ inspirational training* - *Specific/practical skills training*	***Sample size:****n*=184 - Hospital A (*n*=98) - Hospital B (*n*=86) ***Participants characteristics:*** State hospital staff ***Clinical/Recovery experience:*** - Year in current position: 9.0 (SD 9.5) - Year in mental health field: 16.7 (SD 10.3)	- The Life Orientation Test- Revised (LOT-R) (8-items) (α 0.71) - The Recovery Self-Assessment (RSA) (α 0.95) - The Consumer Optimism scale (16-item) (α 0.91)	***Primary outcomes:*** **Optimism and pessimism** 1. The LOT-R - Hospital A: 3.6 (0.7) - Hospital B: 3.8 (0.6) *t*(182) = 1.74 **Degree of implement recovery-oriented practices** 2. RSA - Hospital A: 3.3 (0.4) - Hospital B: 3.2 (0.6) *t*(181) = 0.51 **Optimism regarding patient** 3. The Consumer Optimism scale - Hospital A: 2.7 (0.5) - Hospital B: 3.0 (0.5) *t*(181) = -4.40***	Most staff at the two state hospitals received some recovery-oriented training, mostly general/inspirational training. Staff who received specific/practical training had a greater increase in agency recovery attitudes than staff who received only general/inspirational training or no training. The more trainings staff had, the higher their consumer optimism.
Tsai et al. (2011) USA	To examine whether recovery-related trainings in community mental health centres is associated with differences in staff attitudes and reported organizational practices.	Cross-sectional study *- Recovery-related training*	***Sample size:****n*=318 ***Participants characteristics:*** Community mental health staff - Age: 40.4 [SD 11.9] years - Gender: F = 223 (70.1 %) - Race: White = 240 (75.5 %) - Social Work/Case Management: *n*=189 (70.0 %) - Nursing: *n*=32 (11.9 %) - Psychology/Psychiatry: *n*=13 (4.8 %) - Other: *n*=36 (13.3 %) ***Clinical/Recovery experience:*** - Year in current position: 4.5 (SD 5.1) - Year in mental health field: 10.2 (SD 8.6)	- The LOT-R (8-items) (α 0.78) - The Recovery Self-Assessment (RSA) (α 0.92) - The Consumer Optimism scale (α 0.91)	***Primary outcomes:*** **Optimism and pessimism** 1. The LOT-R - Site A: 3.94 (0.57); Site B: 4.02 (0.62) ; Site C: 3.90 (0.66); Site D: 3.93 (0.57) **Degree of implement recovery-oriented practices** 2. RSA - Site A: 3.74 (0.39) ; Site B: 3.33 (0.61); Site C: 3.69 (0.44); Site D: 3.54 (0.37) **Optimism regarding patient** 3. The Consumer Optimism scale - Site A: 3.38 (0.44); Site B: 3.30 (0.44); Site C: 3.30 (0.58); Site D: 3.04 (0.46)	Compared to staff who had no recovery-related training in the past year, staff who had at least one recovery-related training reported significantly higher consumer optimism and a greater agency recovery orientation towards consumers’ life goals. The number of recovery-related trainings was significantly correlated with scores on personal optimism, consumer optimism, and agency recovery orientation towards consumers’ life goals.
Uppal et al. (2010) Australia	To explore the variables influencing differences in transfer of training (ToT) across mental health settings.	Cross-sectional study *- Collaborative Recovery* *Training Program (CRTP)*	***Sample size:****n*=173 ***Participants characteristics:*** Mental health clinicians from community-based governmental and non-governmental mental health services in eastern Australia ***Clinical/Recovery experience:*** N/R	- The Dartmouth Assertive Community Treatment Scale (DACTS) comprised of three components 1) human resources 2) organizational boundaries 3) nature of services	***Primary outcomes:*** 1. DACTS: - Organizational boundaries: higher caseloads and more frequent client contact	Fewer than half of clinicians implemented collaborative recovery protocols within six months. Better integration, monitoring, incentives, workplace coaching, and strategies to promote clinician responsibility may improve training transfer.
**Qualitative design**						
Clarke et al. (2020) UK	To investigate the experiences of community mental health workers using the REFOCUS intervention to support personal recovery.	Qualitative study *- REFOCUS*	***Sample size:****n*=49 (28 interviews; 24 in focus groups; with 3 staff in both group) ***Participants characteristics:*** Nurse (*n*=14; 50 %) Psychiatrist (*n*=4; 14 %) Psychologist (*n*=2; 7 %) Social worker (*n*=2; 7 %) Occupational therapist (*n*=2; 7 %) Support, Time and Recovery Worker (*n*=3; 14 %) Other (2 Associate practitioner, 1 Physio technician) (*n*=1; 4 %) ***Clinical/Recovery experience:*** N/R	- Qualitative data from semi-structure interview and focus group	***Primary outcomes:*** **Staff’s experiences using REFOCUS** **1. Intervention** - Recovery-promoting relationships - Working practices **2. Implementation strategies** - Information sessions - Personal recovery training and reflection sessions - Coaching for Recovery training - Individual Supervision **3. Practice change** - Staff knowledge of Personal Recovery - Staff attitudes towards Personal Recovery - Staff perspectives on relationships **4. Outcomes** - Empowerment of staff and service users - Team based approach to recovery	Staff valued coaching training and applied the skills to both challenging and motivational conversations with service users. They responded positively to the resources in the ‘working practices’ intervention, and whole-team training and reflection sessions fostered team cultures, structures, and processes that supported recovery-oriented practice.
Edan et al. (2019) Australia	To explore the intersection between implementing ROP training for staff (PULSAR) and working with consumers on CTOs.	Qualitative study - *REFOCUS-PULSAR*	***Sample size:*** - Consumer (*n*=6) - Staff (*n*=3) ***Participants characteristics:*** - Consumers who had participated in the PULSAR trial and having been on a CTO in the previous 12 months: F = 5, M = 1 Mean age = 41.6 (36–58) Australian (*n*=3), Malaysian (*n*=2), Italian (*n*=1) - All staff of teams that participated in the PULSAR training intervention: F = 3, from clinical (*n*=2), from community (*n*=1) ***Clinical/Recovery experience:*** Mean work experience = 14 years (11–28)	- Qualitative data from in-depth interview	***Primary outcomes:*** **Consumer experiences being on CTO** 1. Lacking choice and control 2. Emphasis on medication 3. (Fear) The threat of hospitalisation 4. Absence of recovery-oriented practice 5. Staying supported **Staff working with people on CTO** 1. Recovery Oriented Practice in the presence of CTOs is challenging 2. CTOs are a way to manage risk 3. Lack of focus on recovery as a practice 4. Organisational ‘buy in’ lacking	People on CTOs experienced coercion, reflecting staff views that CTOs served as a risk management tool. Nevertheless, ROP training benefited staff by helping them rethink their practice and implement service-level changes. Although consumers noticed fewer changes, the findings suggested that ROP training and practice support were valuable in services aiming to reduce coercive interventions.
Felton et al. (2006) USA	To extend the field’s understanding of the outcomes of recovery implementation efforts by adding process information about providers’ experiences during classroom-based training.	Qualitative study - *Core Assertive Community Treatment (ACT) training*	***Sample size:****n*=212 ***Participants characteristics:*** ACT team members of all disciplines, including psychiatrists, nurses, social workers, substance abuse specialists, family psychoeducation specialists, and peer counsellors ***Clinical/Recovery experience:*** N/R	- Qualitative data from observation and field's note taking with interface comments analysis by trainers and trainees	***Primary outcomes:*** Endorsement of or difficulties with recovery-oriented practices - Theme 1: ACT is for dual diagnosis - Theme 2: Client won’t admit to being mentally ill - Theme 3: Crisis prevent us from using recovery practices - Theme 4: Client-centred goals - Theme 5: Developing the recipient’s goals - Theme 6: Whose goals? - Theme 7: Making good connection - Theme 8: Who (recipient or provider) should do what? - Theme 9: Symptom Dominant vs Holistic Views of the Recipient - Theme 10: Wellness works	Analysis of trainees’ comments revealed ten themes reflecting support for or challenges with recovery-oriented practice. Key dilemmas included reconciling system-centred and recipient goals, establishing collaborative relationships, and applying a recovery orientation with recipients in crisis or who do not acknowledge their mental illness.
Kehoe et al. (2023) Australia	To explore how consumers perceive their recovery following community mental health staff undertaking specific ROP training.	Qualitative study - *REFOCUS-PULSAR*	***Sample size:*** Consumers (*n*=21): ***Participants characteristics:*** Aged 18–63 years Australian born (*n*=19) East Timor (*n*=1) Malaysia (*n*=1) ***Clinical/Recovery experience:*** N/R	- Qualitative data from 1 to 1 interview	***Primary outcomes:*** **Consumers’ perception on recovery following community mental health staff undertaking specific ROP training** 1. Theme 1: Recovery Needs Connection 2. Theme 2: Relationships That Support Recovery 3. Theme 3: A Better Life 4. Theme 4: Barriers to Recovering	Although staff–consumer relationships were important, consumers’ own meaning of recovery was perhaps more significant. Strong relationships provided hope and helped consumers explore possibilities to build a meaningful life. Consumers emphasised factors supporting recovery, including appropriate medication, community connection, a sense of purpose, and a fulfilling life. Disrupted or poor-fitting staff relationships could lead to disengagement, but positive relationships also strengthened connections.
Leamy et al. (2014) UK	To investigate staff and trainer perspectives on the barriers and facilitators to implementing a complex intervention to help staff support the recovery of service users with a primary diagnosis of psychosis in community mental health teams.	Qualitative study - *REFOCUS*	***Sample size:*** - MH staff interview(*n*=28) - Trainer’s interview (*n*=3) - Focus group x 4 with intervention team (*n*=24) - Written trainer reports (*n*=28) ***Participants characteristics:*** *Professionals in interview and focus group:* Psychiatrist: I = 4 (14 %), F = 2 (8 %) Nurse: I = 14 (50 %), F = 12 (50 %) Psychologist: I = 2 (7 %), F = 1 (4 %) Social Worker: I = 2 (7 %), F = 4 (17 %) Occupational Therapist: I = 2 (7 %), F = 1 (4 %) Support worker: I = 3 (14 %), F = 2 (4 %) Associate Practitioner: I = 1 (4 %), F = 2 (8 % Physio technician: I = 0 (0 %), F = 1 (4 %) ***Clinical/Recovery experience:*** - Interview group: 213.11 (SD 110.228) months - Focus groups: 189.35 (SD 92.723) months	- Qualitative data from interview and focus group	***Primary outcomes:*** **Barriers and facilitators to implementing recovery-oriented complex intervention** 1. Organisational ready for change: (1) NHS Trust readiness; (2) Team readiness; and (3) Practitioner readiness 2. Training effectiveness: (1) Engagement strategies; (2) Delivery style; and (3) Modelling recovery principles	Three findings can inform future implementation and evaluation of complex interventions: (1) key areas for changing practice include staff skill development, intention to implement, and actual implementation behaviour; (2) practitioners infer organisational commitment by observing resource allocation, performance indicators, and service evaluation outcomes; (3) organisational readiness tools can be used as inclusion criteria for selecting organisations and teams in cluster RCTs.
Wallace et al. (2016) Australia	To explore the service user experience of receiving a complex, pro-recovery intervention (REFOCUS), which aimed to encourage the use of recovery-supporting tools and support recovery-promoting relationships.	Qualitative study *- REFOCUS*	***Sample size:*** - Interview (*n*=24) - 2 Focus groups (*n*=13) ***Participants characteristics:*** Service users who received care from teams in the intervention (REFOCUS) arm of the trial Diagnosis: - Schizophrenia: I = 6(25 %); F = 1 (8 %) - Bipolar Disorder: I = 5(21 %); F = 3(23 %) - Depression: I = 2 (8 %); F = 4(31 %) - Anxiety: I = 0 (0 %); F = 1 (8 %) ***Clinical/Recovery experience:*** - Individual interview: 14.3 (SD 11.3) years - Focus groups: 13.0 (SD 9.7) years	- Qualitative data from semi-structure interview and focus group	***Primary outcomes:*** **Service user experience of receiving a complex, pro-recovery intervention (REFOCUS)** - Participants reported that the intervention supported the development of an open and collaborative relationship with staff, with new conversations around values, strengths and goals.	Recovery-supporting tools can foster recovery-oriented relationships, contributing to positive outcomes. They should be used collaboratively and flexibly, with information on values, strengths, and goals incorporated into care planning. As some service users found it difficult to report their experiences, alternative evaluation methods are needed to fully capture their perspectives.
**Mixed method design**						
Daley et al. (2020) UK	To evaluate the feasibility of a staff-level recovery intervention in older people’s mental health services.	Mixed methods: questionnaire (pre-post design) and interview *- The Older Adults Recovery Intervention (OARI)*	***Sample size:****n*=204 - Qualitative interview: there are 12 staff participants ***Participants characteristics:*** Multi-disciplinary mental health team members Nurses (*n*=5) Occupational therapists (*n*=3) Social workers (*n*=2) Psychiatrist (*n*=1) Psychologist (*n*=1) Trainers (*n*=4) (2 nurses, 2 service user trainers) ***Clinical/Recovery experience:*** N/R	*Qualitative:* - The Recovery Knowledge Inventory (RKI) - the 7-item Recovery Attitudes Questionnaire (RAQ-7) Qualitative: - qualitative data from in-depth interview	***Primary outcomes:*** Quantitative: **Recovery Knowledge** 1. RKI mean change (SD): role -0.35 (0.62), non-linearity -0.27 (0.58), self-definition -0.21 (0.58), expectation -0.11 (0.93). **Recovery attitudes** 2. RAQ-7 mean change (SD): recovery is possible -0.48 (2.39), recovery is difficult -0.12 (1.53). Qualitative: 1. Reach: Over 70 % of eligible staff completed full recovery training, except psychiatrists, whose lower completion rate wasn't statistically significant (x^2^= 3.18, 5 *df, p* = 0.67). 2. Acceptability: - Enhancers: Service user involvement, trainer-team fit, and trainer skills. - Barriers: using a team approach to the training (due to varying training needs of different professions), a perceived lack of evidence to support recovery, and difficulties by the trainers in some instances in not being able to challenge existing assumptions (about the degree to which team practice was already recovery-oriented) without increasing defensiveness. 3. Feasibility: Only 9 of 15 teams (56 %) received full team training; implementation support and action planning were limited (60 %). 4. Implementation: - Barriers: Lack of recovery focus, minimal managerial support, time constraints, and service changes. - Facilitators: Team ownership, pro-recovery champions, professional identity alignment, and support tools.	A staff-level intervention was tested, providing a basis for further development for large-scale evaluation of its impact on staff and service users. The evaluation data informed the acceptability and utility of intervention elements, supporting the design of a more definitive study, such as a cluster randomised controlled trial of the next OARI iteration. Further research was needed to understand recovery experiences across different service user groups, explore practice implications for staff, and develop valid and reliable measures of recovery for older adults with functional illness and dementia.
Hornik‐Lurie et. al. (2018) Israel	To assess the knowledge, attitudes and practices developed following recovery‐oriented training of nurses and other staff and to identify the benefits and challenges involved in the implementation of recovery‐oriented intervention in psychiatric wards.	Mixed methods: questionnaires and interview *- Recovery‐oriented training interventions*	***Sample size:*** I: *n*=37 C: *n*=35 Interview: *n*=15 ***Participants characteristics:*** Mental health ward staff: I: RMN (*n*=16, 43.2 %), SW (*n*=13, 35.1 %), Other disciplines (*n*=8, 21.6 %) C: RMN (*n*=26, 74.3 %), SW (*n*=4, 11.4 %), Other disciplines (*n*=5, 14.3 %) ***Clinical/Recovery experience:*** N/R	*Quantitative:* - The Recovery Attitudes Questionnaire (RAQ‐7) (α 0.74) - The Recovery Knowledge Inventory (RKI) (α 0.34–0.81) - The Recovery Self‐Assessment‐Revised (RSA‐R) (α 0.95) *Qualitative:* - Qualitative data from qualitative interview	***Primary outcomes:*** *Quantitative:* **Recovery attitude** 1. RAQ‐7 - Intervention group mean score: 31.08 (SD 2.8) - Comparison group mean score: 27.77 (SD 3.39) **Recovery knowledge** 2. RKI - Intervention group mean score: Role and responsibility in recovery (3.84 [SD 0.57]); non-linearity of the recovery process (2.76 [0.93]); role of self-definition and peer support (3.91 [0.59]); expectations regarding recovery (3.11 [0.99]) - Comparison group mean score: Role and responsibility in recovery (3.26 [SD 0.77]); non-linearity of the recovery process (2.39 [0.73]); role of self-definition and peer support (3.66 [0.57]); expectations regarding recovery (2.9 [0.82]) **Recovery-oriented practices implementation** 3. RSA‐R - Intervention group mean score: 3.27 (SD 0.77) - Comparison group mean score: 3.57 (SD 0.60) *Qualitative:* 1. Illness management and recovery benefits: increased interest and understanding of patients and increased emphasis on person‐centred practice. 2. Illness management and recovery challenges: need for more training and supervision, lack of continuity post discharge, difficulty implementing recovery‐oriented interventions in acute states. 3. Peer specialist benefits: direct contribution in staff meetings, indirect contribution to staff. 4. Peer specialist challenges: negative views about peer specialists in psychiatric wards, lack of role clarity. 5. Psychiatric advance directives benefits: consciousness‐raiser. 6. Psychiatric advance directives challenges: resistance and limited practice.	The quantitative outcomes partially confirmed positive changes in attitudes and some practices. Qualitative interviews complemented these findings, revealing greater use of a person‐centred approach and support for patient autonomy. However, they did not find differences between groups in quantitative outcomes per‐training to personal goals or providing individually tailored services.
Nardella et al. (2021) Australia	To evaluate nurses’ knowledge of personal recovery-oriented practice following completion and implementation of the education programme and to evaluate nurses’ perceptions of the provision of personal recovery-oriented practice in the acute mental health service using the Recovery Self-Assessment-Provider scale.	Mixed methods: questionnaire and focus group *- Recovery training programme and the Mental Health Passport (MHP)*	***Sample size:*** *Training intervention:* Group 1: *n*=23 Group 2: *n*=22 *Focus group:* Group 1: *n*=10 Group 2: *n*=3 ***Participants characteristics:*** Nurses working in a mental health clinic in a private healthcare service in Melbourne, Australia *Focus group:* Group 1: *n*=10 (M=3, F=7) Group 2: *n*=3 (M=3, F=0) ***Clinical/Recovery experience***: experience ranged from less than one year to over 30 years.	*Quantitative:* - Recovery Knowledge Inventory (RKI) - Recovery Self-Assessment (RSA-Provider) *Qualitative:* - qualitative data from focus group	***Primary outcomes:*** *Quantitative:* **Recovery knowledge** 1. RKI - Group 1 mean score (SD): Role and responsibility in recovery (3.66 [0.73]); non-linearity of the recovery process (2.32 [0.59]); role of self-definition and peer support (3.90 [0.62]); expectations regarding recovery (2.67 [0.97]) - Group 2 mean score (SD): Role and responsibility in recovery (3.92 [0.68]); non-linearity of the recovery process (2.58 [0.75]); role of self-definition and peer support (3.51 [0.38]); expectations regarding recovery (3.30 [0.81]) **Recovery-oriented practice implementation** 2. Recovery Self-Assessment (RSA-Provider) - Group 1 mean score (SD): life goals 3.28 (0.78), involvement 2.31 (1.28), diversity of treatment options 2.88 (1.10), choice 3.21 (1.05), individually tailored services 3.41 (1.06), inviting factor 4.00 (0.86). - Group 2 mean score (SD): life goals 3.72 (0.52), involvement 2.85 (0.92), diversity of treatment options 3.16 (0.71), choice 3.94 (0.85), individually tailored services 3.78 (0.79), inviting factor 4.18 (0.96). *Qualitative:* 1. Understanding of recovery-oriented practice: - Most nurses felt knowledgeable about recovery-oriented practice, but some identified the need to enhance mental health assessment and communication skills 2. How to embed personal recovery- oriented care into clinical practice: (1) having sufficient knowledge but saw little change in practice post-introduction, (2) daily recovery goals and ward activities were key in acute care, the MHP was useful for initiating recovery discussions (some seen it as equivalent of the nursing care plan). 3. Barriers to consumer participation in recovery focused activities: (1) struggling to engage in recovery during acute admissions, (2) the MHP was difficult to implement in short stays or for electroconvulsive treatment, (3) its complexity was often seen as inappropriate.	This study found that following completion of the staff education programme, participants had a good understanding of the ‘Roles and responsibilities in recovery’ and this was still evident amongst the inpatient nursing team 12 months later. There was also an improvement in nurses’ responses on the ‘Expectation regarding recovery’ subscale at 12 months.
Okamoto et al. (2018) Japan	To implement an experience-based program to promote the understanding of the concept of recovery, which is defined as a meaningful life and valued sense of integrity based on subjective and individual viewpoints, among psychiatric nurses working in hospitals in Japan and to evaluate this program.	Mixed-methods study: pretest/posttest design and observation *- Experience-based program for understanding the concept of recovery*	***Sample size:****n*=9 ***Participants characteristics:*** Nurses with at least 3 years of clinical experience in psychiatric nursing F= 8, M = 1 Age mean = 40.56 ***Clinical/Recovery experience:*** 123.78 months	*Quantitative:* - The Japanese versions of the 7-item Recovery Attitudes Questionnaire (RAQ-7) - The Recovery Knowledge Inventory (RKI) *Qualitative:* - Qualitative data from observational practice experiences	***Primary outcomes:*** *Quantitative:* **Recovery attitudes** 1. RAQ-7 - Pre-training mean score: 28.00 (SD 3.81), z = −1.496 - Post-training mean score: 27.22 (SD 3.38) **Recovery knowledge** 2. RKI - Pre-training mean score: 3.41 (SD 0.28), z = −2.668, *p* = 0.004 - Post-training mean score: 3.69 (SD 0.24) *Qualitative:* 1. Continuing to attend to the need to live in one’s community/home regardless of how bad psychiatric symptoms become without the use of medicines. 2. Viewing the person living their life in a place where they belong and in their own individual style. 3. Valuing the patient’s wishes is the slow but sure way to a fruitful relationship. 4. Become familiar to the patient and their family’s lifestyle by carefully listening to the family’s feelings.	Recovery orientation significantly increased among the nine program participants. Qualitative analysis revealed multiple expressions of recovery in participants’ ACT support experiences. The program effectively enhanced nurses’ understanding of recovery, demonstrated practical methods for recovery-oriented care, and highlighted current limitations in hospital nursing support. Participants’ experiences may help establish recovery-oriented practice in hospitals that typically follow a medical model and promote strength-based daily nursing care.
Repique et al.(2016) USA	To reduce the use of mechanical restraints in a short-stay inpatient psychiatric setting by facilitating change in care delivery through recovery- oriented nursing practice To improve psychiatric–mental health registered nurses (PMH-RNs)'s knowledge of, and attitudes toward, recovery-focused mental health treatment principles.	Mixed methods: pre-posttest design and focus group - *The SAMHSA training program*	***Sample size:****n*=87 ***Participants characteristics:*** MHNs employed at the hospital The average age = 43 years ***Clinical/Recovery experience:*** 16 participants (38.1 %) had over 10 years of nursing experience and 14 (35.9 %) had 5–10 years of psychiatric nursing experience.	*Quantitative:* - The Recovery Knowledge Inventory (RKI) - Restrain rate *Qualitative* - Qualitative data from focus group	***Primary outcomes:*** *Quantitative:* **Staff recovery knowledge.** 1. RKI - Pre-training mean score: Role and responsibility in recovery (3.74 [SD 0.72]); non-linearity of the recovery process (2.49 [0.67]); role of self-definition and peer support (4.13 [0.54]); expectations regarding recovery (3.40 [0.95]) - Post-training mean score: Role and responsibility in recovery (3.90 [SD 0.64]); non-linearity of the recovery process (2.40 [0.57]); role of self-definition and peer support (4.25 [0.51]); expectations regarding recovery (3.32 [0.98]) **Restrain** 2. Restrain rate - pre-training (Q1 and Q2): 1.48 episodes per 1000 patient days - during training (Q3):.33 episodes per 1000 patient days - post-training (Q4): 2.29 episodes per 1000 patient days *A slight reduction was observed three months post-intervention (Q4 vs. Q3) *Qualitative:* 1. The clarity of the material and the strength of the evidence on mental health recovery: (1) described as “good”; (2) well-presented but felt they already knew; (3) felt material was nothing new for them; (4) preferred a different training more tailored and applicable to inpatient settings; (5) not specific enough; (6) want specific examples of how to put into practice in an acute care psychiatric hospital. 2. The environmental factors influencing the adaption of the recovery principle in practice: (1) practice settings doing a “decent job” with incorporating recovery principles, (2) motivation engagement and integration of recovery principles, (3) wanting to work with a peer support specialist, (4) buy in to the recovery principles starting from the top with leadership. 3. The implementation process of the training programme by using focus group: (1) implementation process need improvement, (2) prefer it being delivered by a live presenter for more interactive, (3) being able to ask questions (human touch), (4) If webinar conducted, having a content expert on-site is needed, (5) more inclusivity of mental health technicians.	Reducing and eliminating restraint in acute psychiatric settings required visionary leaders who recognised the key role of mental health nurses. As seen in this study, such leaders set clear agendas for organisational change through preventative interventions at both system and unit levels. Implementing recovery-oriented principles as an evidence-based restraint-reduction strategy through staff education was iterative, requiring sustained support and strong leadership commitment, as incremental improvements took time and repeated educational initiatives to become embedded and sustained in clinical practice.
**Review**						
Eiroa-Orosa et al. (2019) Spain	To systematically review and synthesize studies assessing awareness and training activities for mental health professionals covering aspects related to recovery, empowerment, and in general, rights-based care to achieve full citizenship of mental health services users.	Systematic review and meta-analysis: 26 papers included 14 quantitative for meta-analysis, and 12 qualitative - *Recovery Educational Interventions*	Age range = N/R Mean age = N/R Gender = N/R	- Change in Knowledge - Change in Recovery Attitudes - Change in Recovery‐Based Practice	***Primary outcomes:*** **Clinician outcomes:** Change in Knowledge; Change in Recovery Attitudes; Change in Recovery‐Based Practice	Recovery training activities appeared to have a moderate impact on mental health professionals’ beliefs and attitudes, but their effect on practice remained unclear. Qualitative evidence suggested that organisational obstacles may have hindered change. Mixed-methods approaches were essential to further explore the potential of recovery training. Future studies were recommended to involve service users not only as trainers or peer-support workers but also in the design and implementation of recovery interventions.
Hawsawi et al. (2021) Australia	To explore to what extent reported recovery-focused educational programmes, addressed nurses’ learning needs in relation to transforming their knowledge and attitudes into practice that supports consumer recovery	Integrative systematic review: (39 included studies; identified 35 programmes and 55 educational materials) - *Recovery-focused educational programmes*	Age range = N/R Mean age = N/R Gender = N/R	N/R	N/R	Findings highlighted the value of consumer involvement in all aspects of the recovery-focused educational programme, which produced positive outcomes. However, consumers’ self-rated outcomes were not associated with improvements in nurses’ performance.
Jackson-Blott et al. (2019) UK	To explore the quantitative literature regarding recovery-oriented training programmes for mental health professionals.	A narrative literature review: (17 included studies) *- Recovery-oriented training programmes*	Age range = N/R Mean age = N/R Gender = N/R	- STARS - TOS - CAI - RAQ - AQ-27 - RPRS - MHRM - QPR - VOTE - RSA-D	***Primary outcomes:*** **Clinician outcomes:** The Staff Attitudes to Recovery Scale (STARS); The Therapeutic Optimism Scale (TOS); The Competency Assessment Instrument (CAI); The Recovery Attitudinal Pre-Post Survey; The Recovery Attitudes Questionnaire (RAQ); The Attribution Questionnaire-27 (AQ-27) **Patient outcomes:** The Dutch version of the Recovery-Promoting Relationship Scale (RPRS); The Dutch version of the Mental Health Recovery Measure (MHRM); The Questionnaire about Process of Recovery (QPR) **Organisational level outcomes:** An audit of service-user care-plans; The Views of the Therapeutic Environment (VOTE); The German version of the Recovery Self-Assessment scale (RSA-D); The Client Service Receipt Inventory	The recovery-oriented training programmes had the potential to improve the recovery-consistent knowledge, attitudes, and competencies of MHPs. There was, however, limited evidence regarding sustained change. Moreover, there was limited evidence relating to service-user and service-level outcomes, suggesting that staff recovery training may have had limited utility in influencing clinical practice.
**Other design**						
Oades et al. (2005) Australia	To evaluate the effectiveness of CRM.	The development of Collaborative Recovery Model - *Collaborative Recovery* *Training Program (CRTP)*	***Sample size:*** N/R ***Participants characteristics:*** N/R ***Clinical/Recovery experience:*** N/R Predominantly for clinical staff, but consumers advocates are encouraged to attend	N/R	N/R	Collaborative recovery training led to immediate improvements in staff knowledge and attitudes regarding recovery for consumers; however, the formal evaluation was still pending.
Slade et al. (2015b) UK	To develop a theoretically based and empirically defensible new pro-recovery manualised intervention – called the REFOCUS intervention.	The development of REFOCUS training intervention *- REFOCUS*	***Sample size:*** N/R ***Participants characteristics:*** N/R ***Clinical/Recovery experience:*** N/R Multidisciplinary community mental health teams, providing case management primarily through patient (typically aged 18–65)	N/R	N/R	The REFOCUS intervention had two components: recovery-promoting relationships and working practices. Relationship support included staff coaching skills, developing shared team understanding of recovery, exploring staff values, a Partnership Project with service users, and raising patient expectations. Working practices focused on understanding values and treatment preferences, assessing strengths, and supporting goal-striving."

**Note:** α = Cronbach’s alpha, χ2 = Chi-square statistic, ACT = Assertive Community Treatment, ARQ = The Attitudes towards Recovery Questionnaire, C = control group, CI = Confident interval, CRM = The Collaborative Recovery model,

CRTP = Collaborative Recovery Training Programme, CTO = Community Treatment Order of a Mental Health Act, *d* = Cohen’s d, *df* = degrees of freedom, F = female, HCPs = healthcare professionals, I = intervention group,

IMR = Illness Management and Recovery, INSPIRE = A questionnaire designed to assess how well mental health services support personal recovery from the perspective of service users, IQR = Interquartile range. LOT-R = the Life Orientation Test- Revised, M = male, MHNs = mental health nurse, MHPs = Mental health professionals, N/R = not reported, NHS = National health service, *p* = probability value, Q1 = Quarter 1, Q2 = Quarter 2, Q3 = Quarter 3, Q4 = Quarter 4,

QPR = The Questionnaire about Processes of Recovery, *r* = correlation coefficient, RAQ = The Recovery Attitudes Questionnaire, RCT = Randomised control trial, REFOCUS = A recovery-oriented training intervention originated in the United Kingdom, REFOCUS/PULSAR = The adapted version of the REFOCUS for Australia, RFEP = Recovery-focused educational programme, RKI = The Recovery Knowledge Inventory, SD = Standard deviation, UK = United Kingdom, USA = United state of America, WRAP = Wellness Recovery Action Plans.

The included studies involved a total of 17,791 healthcare professionals, peer support workers and service user participants. These included mental health nurses (*n*=4) (Nardella et al., 2021, Okamoto and Tanigaki, 2018, Repique et al., 2016, Zuaboni et al., 2017) and general practitioners (*n*=2) (Enticott et al., 2021, Slade et al., 2015a), and the remaining studies included multidisciplinary teams comprised social workers, nurses, occupational therapists, psychologists, psychiatrists, substance abuse specialists (Felton et al., 2006), peer support roles (Deane et al., 2014), and mixed professional roles. Study sample sizes ranged from 9 to 4124 participants, aged between 18 and 75, with one study intervention developed for an older adult setting for those aged 65 and over (Daley et al., 2020). Ethnicity or nationality were reported in three studies (Edan et al., 2019, Kehoe et al., 2023, Young et al., 2005), with the majority of the sample identified as White (*n*=178), Australian (*n*=22), African American (*n*=24), Hispanic (*n*=38), American Indian (*n*=7), Malaysian (*n*=3), Indian (*n*=1), and East Timor (*n*=1). Health professionals’ clinical experience of working in mental healthcare settings ranged between 5.4 to 28 years.

Only four studies (Edan et al., 2019, Mak et al., 2019, Wallace et al., 2016, Wilrycx et al., 2015) reported on the inclusion of service user perspectives. Service users had various diagnoses, including schizophrenia, dual diagnosis, major depressive disorder, bipolar disorder, severe mental illness, and individuals under community treatment orders (a legal mandate for compulsory community-based mental health treatment where risk management is prioritised). Studies were conducted across a range of mental health settings, including adult community mental health services (Deane et al., 2014, Edan et al., 2019, Kehoe et al., 2023, Leamy et al., 2014, Mak et al., 2019, Meadows et al., 2019, Oades et al., 2005, Slade et al., 2015a, Tsai et al., 2011, Wallace et al., 2016, Young et al., 2005), inpatient mental health units/hospitals (Giusti et al., 2022, Okamoto and Tanigaki, 2018, Repique et al., 2016, Williamson et al., 2023, Zuaboni et al., 2017), a mental health rehabilitation service (Oades et al., 2005), an older people mental health service (Daley et al., 2020), a private mental health clinic (Nardella et al., 2021), and patient-supported housing (Oades et al., 2005). Whilst there was no study conducted in substance misuse service, one study included participants with substance-related disorder (Wilrycx et al., 2015).

Nine of the included quantitative studies reported on a range of primary outcomes, including patient personal recovery (Meadows et al., 2019, Slade et al., 2015a, Wilrycx et al., 2015), clinician competencies and care processes and the formation of mutual support (Wilrycx et al., 2015, Young et al., 2005), the quality of clinical goal setting and action planning (Deane et al., 2014), the recovery orientation of health services (Zuaboni et al., 2017), the restraint rate (Repique et al., 2016), and service user optimism (Tsai et al., 2010, Tsai et al., 2011). Most of the qualitative studies assessed primarily staff recovery-related knowledge and attitudes.

Three studies (Enticott et al., 2021, Meadows et al., 2019, Slade et al., 2015a) reported secondary outcomes focusing on service users’ hope, quality of life, empowerment, wellbeing, met and unmet needs, satisfaction and experience of recovery support, perceptions of mental health care and perceived needs, and the status of their mental health conditions (e.g., anxiety and depressive symptoms). Secondary staff outcomes included staff engagement with personal recovery training, staff recovery practice skills, and providers’ general and personal treatment expectations (Salgado et al., 2010).

### 3.3 Methodological quality

Twenty-seven of the studies (96.42 %) demonstrated good quality, surpassing 70 % of the quality appraisal checklist, whilst one study (3.57 %) received a rating of moderate quality **(Supplementary Material 2)**. Of the three randomised controlled trials, one was classified as "low risk of bias" (Meadows et al., 2019) and another as having "some concerns" (Slade et al., 2015a), primarily due to allocation status being poorly concealed from participants and assessors. The last trial was categorised as "high risk of bias" (Mak et al., 2019), due to the non-concealment of participant allocation status, compounded by their placement on a waitlist with no intervention provided during the trial.

### 3.4 Research question 1: what are the characteristics of recovery-oriented training programmes implemented in mental health and substance misuse services?

To retrieve training manuals, emails were sent to all authors. Five responded, indicating that their manuals were not available for sharing. However, two manuals were found to be available online (Meadows et al., 2019, Slade et al., 2015a).

Seven categories and 20 recovery-oriented training programmes were identified, according to TIDieR checklist, based on their primary attributes and underpinning theoretical frameworks, as presented in Table 3: These included 1) Collaborative Recovery Model programmes (*n*=2); 2) Substance Abuse and Mental Health Services Administration agency (SAMHSA) model (*n*=3); 3) Staff training interventions based on the CHIME framework, a model of personal recovery in mental health highlighting five key processes: Connectedness, Hope, Identity, Meaning, and Empowerment (*n*=4); 4) Illness Management and Recovery programme (*n*=1); 5) Bedregal personal recovery model (*n*=1) (Giusti et al., 2022); 6) Illness Management and Recovery and Wellness Recovery Action Plans self-management programmes (*n*=3); and 7) Other theories/principles (*n*=6). Further details are provided in Table 3 and **Supplementary Material 3**.

**Table 3:** The categories of recovery-oriented training intervention and their key components*.*

**Training categories**	**Recovery-oriented training programmes**	**Reported studies**	**Key components**
CRM-based	*Collaborative Recovery Training Programme (CRTP)*	Oades et al. (2005) Crowe et al. (2006) Salgado et al. (2010) Uppal et al. (2010)	The CRTP was developed and implemented in Australian community mental health and rehabilitation services to integrate motivational enhancement and collaborative goal setting, supporting consumers to set, pursue, and achieve personal goals. **Six modules:** 1. Recovery as an individual process emphasis on psychological recovery (i) hope; (ii) meaning; (iii) identity; and (iv) responsibility 2. Collaborative and autonomy support focuses on working alliance barriers to collaboration autonomy 3. Change enhancement focuses on motivational readiness, importance and confidence, and stage of health behaviour change cognitive capacity 4. Collaborative needs identification focuses on unmet needs and motivation and negotiated need 5. Collaborative goal striving focuses on personal recovery vision goal (identification, setting, striving), meaning (autonomous goals), prevention and promotion goals proximal and distal goals 6. Collaborative task striving and monitoring focuses on homework, generalization and reinforcement, self‐efficacy, self‐management, and responsibility
	*The CRM staff development programme*	Williamson et al. (2023)	The CRM staff development programme was designed and implemented for mental health professionals in Australian services, with a focus on well-being and resilience. ***Two main principles:*** 1. Recovery as an individual process 2. Collaboration and autonomy support ***Four key components:*** 1. Change enhancement 2. Collaborative strengths and values identification 3. Collaborative visioning and goal striving 4. Collaborative action planning and monitoring
CHIME-based	*REFOCUS*	Leamy et al. (2014)Slade et al. (2015b) Slade et al. (2015a) Wallace et al. (2016) Clarke et al. (2020)	REFOCUS is a team-level training intervention in the UK designed to enhance staff behaviour by emphasising the values, preferences, strengths, and goals of patients with psychosis, and by strengthening staff–patient relationships through coaching and partnership to improve recovery outcomes. ***Two components:*** 1. Recovery-promoting relationships: 1) Coaching skills training for staff 2) Developing a shared team understanding of recovery 3) Exploring staff values 4) A Partnership Project with people who use the service and raising patient expectations 2. Working practices: 1) Understanding values and treatment preferences 2) Strength’s assessment 3) Supporting goal-striving
	*REFOCUS-PULSAR*	Meadows et al. (2019) Edan et al. (2019) Enticott et al. (2021) Kehoe et al. (2023)	PULSAR is a staff training intervention adapted from REFOCUS for Australian clinical and community mental health services. **Part 1: modular mental health skill training (MHST) component** 1. *Module 1* (core module): operationalising recovery-oriented practice (ROP) in general practice; and enhanced understanding of the perspective of consumers and carers in the provision of mental healthcare 2. *Module 2* (clinical enhancement module): developing of skills in the detection and assessment of Schizophrenia; an ability to apply the principles of ROP to treatment planning and monitoring; the ability to develop recovery-focused mental health treatment plans; and an applied understanding of review processes and relapse prevention strategies for mental illness within a ROP framework **Part 2: Optional Active Learning Sessions** 3. *Module 3* (optional): monthly 1-h online sessions called “PALS (PULSAR Active Learning Sessions)” with a consultant specialist psychiatrist to review, reflect and share their experiences in the implementation of ROP
	*Recovery-oriented training program*	G.K. Wilrycx et al. (2012) Wilrycx et al. (2015)	The recovery-focused training programme developed in the Netherlands was led by an expert with experience in both peer support and professional rehabilitation, aimed at cultivating a recovery-oriented attitude among professionals. **Two main modules:** 1. The basics of recovery-oriented care to familiarise the professional with the concept of recovery 2. The recovery-oriented attitude of the professional
	*Recovery-oriented nursing training programme*	Zuaboni et al. (2017)	A recovery-oriented training programme for mental health nurses in Switzerland, focused on personal recovery and social inclusion using REFOCUS training materials. **Five training sessions:** 1. Session 1: personal recovery and social inclusion based upon the REFOCUS training material 2. Session 2 and 3: basics of Motivational Interviewing 3. Session 4: goal attainment strategies and goal attainment scaling 4. Session 5: implementation of the training contents into care process and documentation, as well as cooperation with other professional groups
SAMHSA-based	*The SAMHSA training programme*	Repique et al. (2016)	The SAMHSA training programme covers patient engagement models, trauma systems theory, and restraint reduction strategies. **Five main topics:** 1. Patient engagement models 2. Trauma systems theory 3. Restraint reduction strategies 4. Integration of peer-to-peer services in psychiatric treatment 5. Outcomes of randomised trial of consumer-managed alternative mental health treatment programmes
	*The Brief Recovery Psychoeducation Programme* for Service Providers	Mak et al. (2019)	The Brief Recovery Psychoeducation Programme covers recovery principles, contrasts medical and rehabilitation models with consumer-oriented approaches, considers the perspectives of people with mental illness and their carers, highlights good recovery practices, and addresses potential challenges and dilemmas. **1. Didactic teaching:** - Introduction to different aspects of recovery - Comparison of the medical and rehabilitation models of recovery with consumer-oriented recovery - Perspectives of people with mental illness and family carers - Action to be done by various parties - Existing good practices of recovery-oriented care - Possible challenges and dilemmas **2. Interactive games / videos Sharing:** - Debriefing after each activity to clarify the idea being introduced - Service users and family carers shared their pathways of recovery **3. Discussion:** - Reflection about the understanding and meaning of recovery - Discussion on how recovery elements can be applied in different scenarios - Identified the potential challenges and barriers to the implementation of recovery-oriented care. **4. Presentation (only for service providers):** - Presentation on the summary of group discussion regarding the adoption of recovery-oriented care for people with different backgrounds and needs - Feedback and suggestions to address the concerns about implementing recovery-oriented practices **5. Quiz:** - Short quiz to revisit recovery concepts being introduced
	*General/inspirational training*	Tsai et al. (2010)	The general/inspirational training aims to prompt staff reflection on their practices and encourages the use of planning rooms as calming spaces for clients to reduce stress and minimise the need for restraints and seclusion. 1. Roadmap to Seclusion and Restraint Free Mental Health Settings: 3-day workshop developed by SAMHSA which engages staff to think about their practices 2. ‘‘Comfort room’’ workgroups: staff are planning rooms to provide a calming environment for clients to relieve stress, which have been proposed to reduce the use of restraints and seclusion 3. ‘‘Bridge building’’: de-escalation techniques and emphasizes use of the least restrictive methods necessary 4. Respect seminars: 1-day presentation by a private consultant and former consumer, Joel Slack, who is a well- known speaker on recovery
IMR-based	*Recovery-related training*	Tsai et al. (2011)	A two-day recovery training for community health staff aimed to help consumers set and achieve personal recovery goals, gain knowledge, and manage their illnesses independently. 1. A two-day IMR training 2. A one-day IMR case consultation workshop
Bedregal’s personal recovery model based	*The Personal Recovery Training Programme (PRTP)*	Giusti et al. (2022)	The Personal Recovery Training Programme covers mental health outcomes, principles of personal recovery, and group activities designed to enhance knowledge and practice while empowering recovery implementation. 1. Concepts of mental health outcomes 2. Concepts and principles of the "personal recovery" process 3. Group works targeting mental health and recovery knowledge/practice improvement 4. Empowerment recovery translation to practice
Multiple recovery theories based	*Core Assertive Community Treatment (ACT)*	Felton et al. (2006)	The Core Assertive Community Treatment intervention, based on the Wellness Recovery Action Plans (WRAP) and IMR recovery frameworks, supports staff in engaging clients by helping them develop personalised recovery plans. **Three main components in recovery module:** 1. Models of recovery and described practices such as Deegan (2000) “key elements in supporting recovery” 2. “Recovery-centred service planning” describing methods of enacting, through service plans, the recovery principles 3. WRAP planning (Copeland, 2002).
	*Specific/practical skills training*	Tsai et al. (2010)	The Specific/practical skills training is grounded in IMR, WRAP, Integrated Dual Disorders Treatment, the Matrix model, and Motivational Interviewing. **Specific/practical skills training** 1. *Illness Management and Recovery:* a curriculum-based treatment approach focused on teaching consumers how to set and achieve personal recovery goals, acquire knowledge, and use skills to independently manage their illnesses 2. *Integrated Dual Disorders Treatment:* teaches staff to provide mental health and substance abuse interventions together based on clients’ stage of treatment and readiness to change 3. *Wellness Recovery and Action Planning:* teaches staff to engage clients in their own care and personal goals by helping them develop specific recovery plans 4. *The Matrix model:* a structured treatment approach for substance abuses that staff can use to provide information and relapse prevention techniques 5. *Motivational interviewing:* teaches staff how to use their clients’ motivations and resources to change their behaviour
	*Recovery-oriented training*	Hornik‐Lurie et. al. (2018)	A recovery-oriented training intervention encompassing IMR, peer support, and psychiatric advance directives. *1. Illness management and recovery training:* - A psychoeducational, evidence‐based intervention, which supports individuals in developing the requisite knowledge and tools for optimal self‐management of their mental illness and personal goals, according to one’s vision of their own recovery. - Staff are trained to develop personal relationships and to focus on positive aspects, creating trust and hope about the ultimate attainment of personal goals. 2. *Work with Peer support within the team:* - Ten peer support workers and a peer supervisor were introduced into the Beer‐Sheva Mental Health Centre in 2015, part of a special demonstrative project supported by the National Insurance Institute of Israel and Ministry of Health. - After initial training, peer support workers receive ongoing supervision. They work in different wards across the hospital, participate in multidisciplinary team activities and provide individual and group interventions. *3. A psychiatric advance directive training:* - To enables individuals receiving psychiatric care to declare, in advance, their preferences and directives (e.g., type of service, medication, key contacts) in the event of a mental crisis and subsequent loss of capacity to exercise independent judgement. - To protect the self‐dignity of the patient and safeguard one’s preferred treatment of choice. - To encourage proactive patient participation in their own treatment and well‐being.
Other theory based	*The Older Adults Recovery Intervention (OARI)*	Daley et al. (2020)	The Older Adults Recovery Intervention aims at enhancing staff pro-recovery practices. **Three modules:** 1. *Promoting Recovery:* what is recovery and what is recovery-oriented practice, as well as practical exercises whereby staff share relevant clinical experiences, and consider how the training content can be delivered within routine practice 2. Maintaining Identity 3. Enhancing Resilience *Each module is delivered sequentially, with homework from the previous module*
	*Consumer-led intervention/Staff Supporting Skills for Self-Help*	Young et al. (2005)	The Consumer-led Staff Supporting Skills for Self-Help intervention, grounded in an emerging national movement of consumers with severe mental illness. 1. Scientific presentation on self-help: - Assess clinicians’ previous support of self-help and empowerment - Present scientific material about recovery, rehabilitation, and self-help - Discuss ways to involve consumers in increasing self-help and mutual support 2. Structured dialogues: - Hold small groups with equal numbers of consumers and clinicians - Focus on barriers to self-help - Discuss hopelessness regarding severe and persistent mental illness and compare with experiences that create hope - Discuss factors that impede and promote recovery, how self-help complements traditional treatment, and resistance to self-help 3. Rehabilitation readiness: - Present clinicians with rehabilitation readiness concepts and skills to help consumers set goals and develop coping strategies - Present information about how clinicians can manage their own stressors, consumer demands, and the larger mental health system 4. Strategies for independence: - Focus on strategies for minimizing consumer dependence on mental health professionals - Discuss consumer responsibility for recovery, consumer and clinician behaviours that interfere with progress and reduce quality of life, and strategies to help clinicians tolerate their discomfort with consumers practicing new behaviours 5. Professional skills supporting self-help: - Use small groups and role-playing techniques - Focus on helping staff understand how to support self-help without being intrusive - Present theories about self-help success, the characteristics of common mutual support groups, and how traditional treatment can support consumers’ progress regarding self- help 6. Detailing: - Continue to meet as needed with clinicians. Provide individual advice, group presentations, and role-playing techniques for problems
	*The experience-based recovery-oriented training programme*	Okamoto et al. (2018)	The experience-based programme was designed to help nurses in Japanese psychiatric wards understand the principles of recovery. **Day 1:** 1. *Lecture Group work (90* min*):* Rethink how to understand families and their characteristics and how to offer family support. Use specific case examples to clarify emotional pain of the family and effects on recovery, establish a support hypothesis, and clarify goals. 2. *Lecture (90* min*):* Learn about goals and specific content of ACT support at private medical facilities (complete internal type) including attitudes on recovery, outreach/community care, and ACT characteristics, history, elements, effects, and issues. 3. *Survey report (30* min*):* Listen to presentations on the results of an interview survey regarding the thoughts of families who are users of ACT. **Day 2:** 4. *Observational practice (540* min*):* Experience of a single day of ACT. After participating in a pre-visit team meeting, accompany staff on providing visiting support to users and receive feedback from staff after completing the practical work. **Day 3:** 5. Group work (120 min): Group work and question and answer session on experience presentations and clinical cases after completing the observational practice. 6. Lecture (90 min): Learn about coordination between hospitals and communities, the fundamentals and elements of community lifestyle support (case management, recovery and strengths, outreach, multidisciplinary teams, social inclusion), mental health care centre support systems, public facility (network type) ACT implementation support goals, and the details and issues associated with specific support methods.
	*Recovery-based training*	Walsh et al. (2017)	A recovery-based training, guided by adult education principles and reflective practice, was delivered for health professionals in Ireland. **Training components** (4-hour training workshop)**:** 1. Defining the concept of recovery 2. Exploration of the recovery principles and how these recovery principles can be adopted into clinical practice
	*Recovery training programme*	Nardella et al. (2021)	**Staff (nurses) education workshop in personal recovery-orientated practice (60 min)** Topics included therapeutic facilitation, motivational interviewing, initiating recovery conversations, the principles of recovery, and trauma-informed care.
	*Skills acquisition coaching and Transformational coaching (after CRM training in first two days)*	Deane et al. (2014)	**Skills condition coaching:** - Skills condition coaches were trained to address problems with the implementation of the CRM that coachees brought to the session. - Focused on identifying and exploring solutions to organisational and personal barriers to implementation of the CRM with their clients. - Including clinical relationship issues, change enhancement strategies or the use of the LifeJET protocols for values clarification, goal setting or action planning. **Transformational coaching:** - Transformational coaches were trained to use the LifeJET protocols to assist in the coachees’ personal or professional development by continuing to explore their personal values, vision and important goals. - Transformational coaching paralleled the coaching-style approach participants were using with clients.

**Note:**

**Training intervention categories:**

**1. CRM-based:** Trainings based on the Collaborative Recovery Model support personal recovery by using motivational enhancement and collaborative goal setting to help service users identify strengths, set goals, and work with practitioners to achieve meaningful outcomes.

**2. CHIME-based:** Trainings based on the CHIME framework promote personal recovery by fostering **C**onnectedness, **H**ope, **I**dentity, **M**eaning, and **E**mpowerment in service users, enhancing staff focus on patients’ values, preferences, strengths, and goals, and strengthening staff–patient relationships through coaching and partnership.

**3. SAMHSA-based:** Trainings based on the SAMHSA (Substance Abuse and Mental Health Services Administration) model promote recovery through hope, person-driven care, holistic approaches, peer support, and strengths-based practices.

**4. IMR-based:** Trainings based on IMR (Illness Management and Recovery) model help service users manage their illness, reduce symptoms, prevent relapses, set personal goals, and develop skills for long-term recovery.

**5. Bedregal’s personal recovery model based**: Training based on Bedregal’s personal recovery model promotes recovery through consumer-directed, strengths-based care that supports self-determination, cultural competence, symptom management, meaningful activities, hope, overcoming stigma, and recognition of recovery as an individual.

**6. Multiple recovery theories based:** Trainings integrating multiple recovery frameworks or models from other categories.

**7. Other theory based:** Trainings based on other evidence or theories; e.g., findings from qualitative research and recovery literature, grounding from an emerging national movement of consumers with severe mental illness and is implemented in community mental health settings, applying assertive community treatment which is an intensive, team-based mental health service model that provides community-based, multidisciplinary support to people with severe mental illness, aiming to reduce hospitalisation, improve functioning, and support recovery in daily life.

ACT = Assertive Community Treatment, CRM = The Collaborative Recovery model, CRTP = Collaborative Recovery Training Programme, IMR = Illness Management and Recovery, LifeJET = Life Journey Enhancement Tools, OARI = The Older Adults Recovery Intervention, REFOCUS = A recovery-oriented training intervention originated in the United Kingdom (this is the intervention name, not an acronym), REFOCUS-PULSAR = An adapted version of RFOCUS for use in Australia (this is the intervention name, not an acronym), SAMHSA = Substance Abuse and Mental Health Services Administration, WRAP = Wellness Recovery Action Plans.

### 3.5 Research question 2: what are the effects of recovery-oriented training programmes on staff, service user and organisational outcomes?

Training outcomes included four categories: 1) participant reactions, 2) staff level learning outcomes, 3) staff behaviour change, and 4) result/outcomes (for service users, organisations, and intervention level outcomes) (Kirkpatrick and Kirkpatrick, 2016). Effectiveness included both staff outcomes (e.g., improvement in staff recovery knowledge and attitudes) and patient recovery outcomes (**Supplementary Material 4**).

#### 3.5.1 Staff’s views and reactions to the training programmes (category 1)

Four studies reported staff reactions to training programmes. Trainees found the REFOCUS programme, which was based on the CHIME approach, empowered them to have motivational conversations with service users and provoked reflection on their own recovery-oriented attitudes and practices as a team (Clarke et al., 2020). Some psychiatrists and team leaders viewed the REFOCUS intervention as supporting their identity and credibility as a recovery team and that it improved teamwork (Leamy et al., 2014). However, some clinicians perceived the intervention as an additional burden, a threat to their professional identity, a criticism of their current practice, or as providing no new learning for them (Leamy et al., 2014). Similarly, trainees undergoing the Substance Abuse and Mental Health Services Administration training (Repique et al., 2016) found the programme to be too basic and lacking in novelty. Staff wanted greater specificity and relevance of training to their own inpatient settings, including practical examples of the application of recovery principles within acute care psychiatric hospitals (Repique et al., 2016). Staff scepticism about the suitability of recovery for certain individuals, combined with misunderstandings about recovery itself, leads to the belief that recovery-oriented training was not appropriate for people with substance misuse issues (Felton et al., 2006).

#### 3.5.2 Staff post-training behaviour change (category 2)

Four studies reported a change in staff behaviour post-training (Clarke et al., 2020, Edan et al., 2019, Slade et al., 2015a, Williamson et al., 2023). Staff trained in REFOCUS (Slade et al., 2015a) reported that the training gave them permission to have more sensitive, previously off-limits conversations with service users about sexuality and spirituality (Clarke et al., 2020, Edan et al., 2019). An evaluation of a programme based on the Collaborative Recovery Model, analysed the language used by staff regarding definitions of recovery, both before and after training (Williamson et al., 2023). They observed a decrease of over 50 % in the use of medicalised language with a reduction in words like "illness" and "symptoms", whilst recovery-oriented language such as "wellbeing" and "resilience" showed a threefold increase.

#### 3.5.3 Staff post-training outcomes (category 3)

*Recovery Knowledge:*

Eleven studies examined staff recovery knowledge post-training (Crowe et al., 2006, Daley et al., 2020, Giusti et al., 2022, Hornik-Lurie et al., 2018, Mak et al., 2019, Nardella et al., 2021, Okamoto and Tanigaki, 2018, Repique et al., 2016, Walsh et al., 2017, Williamson et al., 2023, Wilrycx et al., 2012). An evaluation of the Brief Recovery Psychoeducation Programme (Mak et al., 2019), a culturally appropriate recovery training programme for providers and service users, showed a significant increase in the Recovery Knowledge Inventory (Bedregal et al., 2006) of staff post-training, with a large effect size in the intervention group (*d*=1.06) compared to the control group. Similarly, a modified stepped-wedge study of a staff training programme based on CHIME principles (Wilrycx et al., 2012) reported a significant improvement in the Recovery Knowledge Inventory (Dutch version) following training. Additionally, three quasi-experimental studies (Giusti et al., 2022, Walsh et al., 2017, Williamson et al., 2023) demonstrated enhanced post-training knowledge on the Recovery Knowledge Inventory (Giusti et al., 2022, Walsh et al., 2017) and on ten true-or-false items on recovery knowledge (Williamson et al., 2023). Likewise, quantitative data from mixed-method evaluations of programmes based on a range of different theories indicated significant improvements on the Recovery Knowledge Inventory post-training (Daley et al., 2020, Hornik-Lurie et al., 2018, Nardella et al., 2021, Okamoto and Tanigaki, 2018).

Conversely, one evaluation by Repique et al. (2016) of Substance Abuse and Mental Health Services Administration training, reported no improvement in the knowledge of mental health nurses across four domains of the Recovery Knowledge Inventory (Bedregal et al., 2006). However, Crowe et al.’s (2006) evaluation of a Collaborative Recovery Training Program reported that post-training scores on their developed Collaborative Recovery Knowledge Scale were positively correlated with scores on their Staff Attitudes to Recovery Scale and the Recovery Attitudes Questionnaire (Borkin et al., 2000), suggesting that recovery knowledge is positively linked with recovery attitudes. Therefore, the limited improvement in recovery knowledge observed by Repique et al. (2016) may be due to participants’ attitudes towards training, perceiving it to cover only basic concepts with content that presented no new knowledge.

*Recovery attitudes:*

Eleven studies evaluated attitudes towards the concept of recovery and recovery-based interventions (Daley et al., 2020, Hornik-Lurie et al., 2018, Mak et al., 2019, Nardella et al., 2021, Okamoto and Tanigaki, 2018, Salgado et al., 2010, Tsai et al., 2010, Tsai et al., 2011, Williamson et al., 2023, Wilrycx et al., 2012, Zuaboni et al., 2017). Mak et al. (2019) found that staff exhibited positive attitudes as measured by the Attitudes towards Recovery Questionnaire after engaging in a Brief Recovery Psychoeducation Programme. Mental health care professionals also showed a more positive score in the Recovery Attitude Questionnaire (Borkin et al., 2000) in clinical practice following two intensive recovery-oriented training sessions, based on CHIME framework (Wilrycx et al., 2012). Similarly an evaluation by Salgado et al. (2010) of a Collaborative Recovery Training Programme reported an increased score on the Recovery Attitudes Questionnaire. Another study focusing on Collaborative Recovery Model training (Williamson et al., 2023) showed significant enhancements in staff recovery attitudes post-training measured by 10 items (Crowe et al., 2006) to assess attitude. Furthermore, a supplementary booster training session of the Collaborative Recovery Model displayed further improvements in staff attitudes and self-confidence (Williamson et al., 2023). Two cross-sectional studies (Tsai et al., 2010, Tsai et al., 2011) also found a substantial increase score in the Recovery Self-Assessment (Salyers et al., 2007) in staff who underwent Illness Management and Recovery Training and general recovery training compared to those who did not. Additionally, three studies evaluating training based on a range of different theories (Daley et al., 2020, Hornik-Lurie et al., 2018, Nardella et al., 2021) demonstrated a favourable shift in recovery attitudes, as measured by the Recovery Attitudes Questionnaire and the Recovery Assessment Scale (O'Connell et al., 2005). Conversely, two studies (Okamoto and Tanigaki, 2018, Zuaboni et al., 2017) found no significant differences in staff recovery attitudes and beliefs between intervention and control wards.

*Recovery competency:*

Two studies assessed staff’s competency related to recovery (Wilrycx et al., 2015, Young et al., 2005). Young et al. (2005) evaluated staff’s competencies in assessment, treatment, and rehabilitation using the Competency Assessment Instrument and found greater improvement in education about recovery compared to the control group. However, a longitudinal study of recovery-promoting professional competence among mental health providers, measured by service users using the Dutch version of the Recovery-Promoting Relationship Scale (Russinova et al., 2006, Wilrycx et al., 2012), showed no significant change or improvement over time.

#### 3.5.4 Patient recovery outcomes (category 4.1)

Eight studies reported service user recovery outcomes (Edan et al., 2019, Enticott et al., 2021, Meadows et al., 2019, Repique et al., 2016, Slade et al., 2015a, Tsai et al., 2010, Tsai et al., 2011, Wilrycx et al., 2015). One trial of the REFOCUS-PULSAR programme (Meadows et al., 2019) noted a small enhancement in service user recovery in the intervention group (*d*=0.23) as measured by the Process of Recovery tool (Neil et al., 2009). Similarly, a pre-post study of REFOCUS-PULSAR (Enticott et al., 2021) also indicated a small increase in positive service user recovery outcomes (*d*=0.29) following recovery training for general practitioners. However, some service users reported no difference in the level of recovery support provided by staff who had undergone training compared with those who had not (Meadows et al., 2019). Repique et al. (2016) also reported a minor decrease in the use of restraint recorded at three months (from 2.33 to 2.29 episodes per 1000 patient days) following Substance Abuse and Mental Health Services Administration staff training.

In contrast, the REFOCUS intervention (Slade et al., 2015a) failed to demonstrate a significant impact on the recovery of people with psychosis compared to standard care. Similarly, the study by Wilrycx et al. (2015) reported no service user improvement on most domains of the Mental Health Recovery Measure. A cross-sectional study (Tsai et al., 2010, Tsai et al., 2011) using the Consumer Optimism Scale (Grusky et al., 1989) also found no differences in service user outcomes at follow-up for those treated in two sites undergoing specific/practical skills training (focussing on recovery-oriented principles) or general/inspirational training (focussing on de-escalation and least restrictive practice).

Two qualitative studies of service users perception on their recovery following community mental health staff participation in the REFOCUS (Wallace et al., 2016) and REFOCUS-PULSAR programmes (Kehoe et al., 2023). Service users found the intervention hope-inspiring and empowering, reporting that it fostered an open and collaborative relationship with staff and encouraged new discussions around values, strengths, and goals (Wallace et al., 2016). However, Kehoe et al. (2023) found that the majority of service users were unable to recall recovery-related language or concepts, even when prompted, with only a small number recognising terms such as "working on a strength".

#### 3.5.5 Service/organisation-level outcome (category 4.2)

Six studies reported service/organisation-level outcomes (Clarke et al., 2020, Leamy et al., 2014, Slade et al., 2015a, Uppal et al., 2010, Williamson et al., 2023, Young et al., 2005). One study of a cost analysis of the REFOCUS intervention (Slade et al., 2015a) reported that the training was associated with an overall reduction in total service costs per user. Whilst qualitative findings of REFOCUS (Clarke et al., 2020) indicated that the group work and reflection had fostered team cultures and relationships, structures and processes that promoted the active development of recovery practice. Trained organisations offered more recovery-oriented services than control sites, with clinicians receiving greater managerial support for implementing the intervention (Young et al., 2005). However, Uppal et al. (2010) found that only 37 % of the trained clinicians were actively implementing the training protocols in their clinical practice. Additionally, an evaluation of training based on the Collaborative Recovery Model, reported no changes in staff confidence of their organisation's implementation of recovery-oriented practice (Williamson et al., 2023). In a qualitative study by Leamy et al. (2014), some clinicians believed that certain tasks in the REFOCUS intervention should be delegated on to care coordinators or support workers with lower pay grades than themselves.

#### 3.5.6 Intervention-level outcomes (category 4.3)

Three studies mentioned intervention-level outcomes (Clarke et al., 2020, Daley et al., 2020, Repique et al., 2016). Qualitative findings from a REFOCUS intervention programme (Clarke et al., 2020) highlighted how clinicians were supported to move beyond the medical-model approach and reconsider clinical decisions where they might have been excessively cautious and protective. Additionally, the training stimulated some staff to reevaluate their belief that recovery from severe mental illness was unattainable and to reassess their assumptions about service users' motivations, capabilities, goals and personal attributes (Clarke et al., 2020). An evaluation of the Older Adults Recovery Intervention reported that, whilst staff acknowledged the applicability of training to a diverse range of service users, this broader approach provided limited specific recommendations for their own practice (Daley et al., 2020).

### 3.6 Research question 3: what the enabling and hindering factors that influence the implementation of recovery-oriented training programmes?

Twelve studies identified barriers to implementing recovery-oriented training interventions (Clarke et al., 2020, Eiroa-Orosa and García-Mieres, 2019, Enticott et al., 2021, Hawsawi et al., 2021, Kehoe et al., 2023, Leamy et al., 2014, Mak et al., 2019, Meadows et al., 2019, Nardella et al., 2021, Oades et al., 2005, Walsh et al., 2017, Zuaboni et al., 2017), with more than half of the identified barriers being organisational constraints. Notably, only two studies (Daley et al., 2020, Edan et al., 2019) highlighted enablers as well as barriers.

#### 3.6.1 Barriers

Three main types of barriers to implementing recovery-oriented training programmes were identified: organisational structure changes and workload, professional barriers (e.g., time constraints, philosophical opposition, disengagement), and service user barriers (e.g., severity of health problems).

##### 3.6.1.1 Organisational structure changes and workload

*Organisational structural changes:*

Researchers have suggested that the implementation of recovery-based services can be hampered, particularly in hospital-based services, by the failure to adopt a system-wide approach (Eiroa-Orosa and García-Mieres, 2019, Hawsawi et al., 2021). During an evaluation of a REFOCUS programme, organisational changes, such as the restructuring of services, contributed to an increase in staff turnover, hindering the implementation of recovery-oriented training (Leamy et al., 2014). A further study evaluating the REFOCUS-PULSAR trial, identified that staff motivation to engage in recovery-oriented practices could be impeded by disruptions, challenging staff relationships, and alterations in staff allocation (Kehoe et al., 2023).

*Workload:*

Increased caseloads within the community mental health service placed additional strain on staff members undergoing REFOCUS training, leading them to question their ability to prioritise recovery care and the well-being of patients (Leamy et al., 2014). Two further studies (Mak et al., 2019, Zuaboni et al., 2017) also highlighted the impact of heavy workloads and stressful environments on the implementation of recovery-oriented practice. For instance, Mak et al. (2019) found that healthcare workers in Hong Kong faced extended work hours and substantial workloads, with a significantly higher user-provider ratio compared to Western countries. Another study found that despite initially high motivation among mental health nurses to actively participate in the training, its implementation proved overly ambitious due to their demanding daily ward routines (Zuaboni et al., 2017).

##### 3.6.1.2 Professional barriers

*Time constraints:*

In two studies, researchers reported that staff members were concerned about having sufficient time to engage with consumers with less acute but more long-term needs (Eiroa-Orosa and García-Mieres, 2019, Oades et al., 2005). Similarly, in the community setting, staff faced challenges in maintaining regular contact with each individual on their caseload, even when following the minimum requirements outlined in their clinical protocols (Oades et al., 2005). The researchers evaluating the REFOCUS-PULSAR programme also noted the limited time available for general practitioners to participate in recovery-based training (Enticott et al., 2021).

*Philosophical opposition:*

The predominant focus on clinical recovery of many services remains a common barrier for implementing personal recovery-oriented practice, resulting in limited treatment options for patients (Kehoe et al., 2023, Walsh et al., 2017). Researchers also highlighted a weaker emphasis on personal recovery across teams and service-wide (Daley et al., 2020) and reported the continued use of assessments that prioritised clinical recovery (Hawsawi et al., 2021). Leamy et al. (2014) reported that some staff aiming to safeguard service users perceived their recovery goals as unrealistic or that they lacked sufficient motivation to pursue these goals. In Australia, there were challenges in engaging senior staff in team-based recovery-oriented programmes, with physicians tending to favour profession-specific training (Meadows et al., 2019). This lack of engagement from some psychiatrists appeared to undermine the implementation of recovery practices within teams and hindered subsequent practice changes (Daley et al., 2020).

##### 3.6.1.3 Service users’ barriers

*Service users’ condition:*

In two studies, researchers mentioned that severely ill service users often found it challenging to focus on their recovery during acute care admissions (Edan et al., 2019, Nardella et al., 2021). Sometimes the severity of their illness prevented them from engaging in discussions about recovery or actively participating in their care (Nardella et al., 2021). Staff found it especially challenging to remain focused on implementing recovery-oriented practices when dealing with patients on community treatment orders (Edan et al., 2019).

#### 3.6.2 Enablers

Daley et al. (2020) identified several facilitators to implementing the Older Adults Recovery Intervention training programme in practice. This included having team ownership and a sustained team focus, the presence of pro-recovery champions, training that aligned with staff professional identity, and the utilisation of practice support tools. Edan et al. (2019) emphasised the importance of supporting staff who view recovery-oriented principles as the cornerstone of their practice to motivate and encourage them to prioritise recovery in their work. An emphasis on concepts like the dignity of risk may ultimately foster a sense of continual support for service users (Edan et al., 2019).

## 4 Discussion

This review is the first to expand the eligibility criteria to include non-professional peer support workers and substance misuse services. As an original contribution, the findings categorised recovery-oriented training interventions by their underlying theoretical basis and identified gaps in the evidence, particularly regarding service users’ perspectives and long-term impacts, to guide future research. The importance of these findings lies in providing practical insights for staff training, supporting the adoption of recovery-oriented practices in clinical and community settings, and informing organisational planning and policy by clarifying factors that facilitate or hinder successful implementation.

We have conceptualised key components and evaluated the effects of personal recovery-oriented training interventions, thereby building a solid foundation of knowledge about these interventions and providing insights for enhancing recovery-oriented research and practice.

### 4.1 Characteristics and categories of recovery-oriented trainings

Despite the range of different recovery-oriented training interventions that have been developed for staff, shared commonalities were identified across programmes in terms of their aims, content, and components. Interventions commonly focused upon improving staff understanding of recovery concepts, enhancing staff and team attitudes towards recovery, and fostering supportive and collaborative staff relationships. These findings support those of Alex Mabe et al. (2016), who underscored the importance of promoting recovery-oriented attitudes among staff to reduce stigmatisation and to encourage viewing service users as equal partners in their care. Many also trained staff in techniques to better engage service users in recovery programmes, including effective goal setting, motivational interviewing, coaching, and ways of supporting patients' autonomy, hope, and motivation. Many interventions aligned with widely used recovery frameworks and principles, such as CHIME, the Illness Management and Recovery model, Substance Abuse and Mental Health Services Administration, and Wellness Recovery Action Plan, which enhanced their validity and increased the likelihood of achieving positive recovery outcomes (Bird et al., 2014, Penas et al., 2020).

Most recovery-oriented training interventions included in this review were implemented in secondary care mental health service settings. Only the REFOCUS-PULSAR intervention has been delivered in both secondary and primary care settings, where mental health care is offered by general practitioners. We also failed to find any recovery-oriented training interventions designed for or implemented within substance misuse services. This may reflect the primary emphasis of these types of services on substance cessation, addiction recovery, and addressing underlying conditions (Green et al., 2015), rather than focusing on personal recovery. However, the importance of embedding recovery principles within the substance misuse field is being increasingly recognised (Inanlou et al., 2020).

Most of the training programmes identified were delivered to trained healthcare professionals, with only one study (Williamson et al., 2023) including the use of peer support workers with direct experience of mental illness. Peer support workers are a valuable addition to recovery-oriented programmes, allowing them to share their own lived experiences and provide a positive role model of recovery for service users. Indeed, the recent replication of the REFOCUS trial in France (known as the RETAFORM study) adopted a train-the-trainers implementation strategy, which involved training of both mental health practitioners and peer support workers with lived experience (Dubreucq et al., 2025).

### 4.2 The effects of recovery-oriented trainings

The evidence of the effects of interventions across the four levels of the Kirkpatrick model found in this review revealed a limited focus on long-term behaviour changes and service user outcomes, whilst most emphasis was placed on professional outcomes, such as recovery knowledge and attitudes (Table 4 and **Supplementary Material 4)**. Service users’ perceptions on receiving services from staff who had undergone training interventions were mixed, as only two studies addressed this aspect, highlighting the need for further exploration. Although many studies reported positive outcomes for training programmes under evaluation, a failure to establish effectiveness can also be attributed to inadequate implementation of the intervention rather than to the content of training. For example, in the REFOCUS intervention (Slade et al., 2015a), inadequate implementation by some teams led to no significant impact on patient recovery. This is consistent with the findings of Jackson-Blott et al. (2019), who emphasised the importance of embedding recovery-oriented training within broader organisational changes to ensure its effective translation into clinical practice.

**Table 4:** Summary of recovery-oriented training intervention characteristics and outcomes according to the TIDieR checklist and Kirkpatrick’s model (N=30)*.*

Study	Ix	Training outcomes				Why	Who				How			Where		When and How much						Modification	How well		Total
		Reaction	Learning	Behaviour	Results	Theory	From		To		Deliver			MH	SS	Duration			Intensity				Attrition	Adherence	
							HCPs	PwL	HCP	PSW	FTF	On	Tel			Short ≤1D	Med 1W	Long >1W	Low ≤1S	Mod 2–4S	Hi >4S				
Oades et al. (2005)	CRTP	**—**	**—**	**—**	**—**	●	∅	∅	●	∅	∅	∅	∅	●	∅	—	●	—	—	●	—	∅	∅	∅	5
Crowe et al. (2006)	CRTP	**—**	±	**—**	**—**	●	∅	∅	∅	∅	∅	∅	∅	∅	∅	∅	∅	∅	∅	∅	∅	∅	∅	∅	1
Salgado et al. (2010)	CRTP	**—**	√	**—**	**—**	●	∅	∅	∅	∅	∅	∅	∅	∅	∅	—	●	—	—	●	—	∅	∅	∅	3
Uppal et al. (2010)	CRTP	**—**	**—**	**—**	×	●	∅	∅	∅	∅	∅	∅	∅	∅	∅	—	●	—	—	●	—	∅	∅	∅	3
Williamson et al. (2023)	CRM	**—**	√	√	×	●	●	∅	●	●	∅	∅	∅	●	∅	—	●	—	—	●	—	∅	∅	∅	7
Slade et al. (2015b)	REFOCUS	**—**	**—**	**—**	**—**	●	∅	∅	∅	∅	∅	∅	∅	●	∅	∅	∅	∅	∅	∅	∅	∅	∅	∅	2
Slade et al. (2015a)	REFOCUS	**—**	**—**	√	×	●	●	●	●	∅	●	∅	●	●	∅	—	—	●	—	—	●	●	∅	∅	10
Wallace et al. (2016)	REFOCUS	**—**	**—**	**—**	Q	●	∅	∅	∅	∅	∅	∅	∅	●	∅	∅	∅	∅	∅	∅	∅	∅	∅	∅	2
Clarke et al. (2020)	REFOCUS	±	**—**	√	√(Q)	●	●	●	∅	∅	∅	∅	∅	●	∅	—	—	●	—	—	●	∅	∅	∅	6
Leamy et al. (2014)	REFOCUS	±	**—**	√	±(Q)	●	∅	∅	∅	∅	●	∅	∅	●	∅	—	—	●	—	—	●	∅	∅	∅	5
Wilrycx et al. (2012)	ROTP	**—**	√	**—**	**—**	●	●	●	∅	∅	∅	∅	∅	∅	∅	—	●	—	—	●	—	∅	∅	∅	5
Wilrycx et al. (2015)	ROTP	**—**	**—**	**—**	±	●	∅	∅	∅	∅	●	∅	∅	∅	∅	—	●	—	—	●	—	∅	∅	∅	4
Zuaboni et al. (2017)	ROTNP	**—**	**—**	**—**	×	●	∅	∅	●	∅	∅	∅	∅	●	∅	—	●	—	—	—	●	∅	●	∅	6
Meadows et al. (2019)	REFOCUS-PULSAR	**—**	**—**	**—**	±	●	●	●	●	∅	●	∅	∅	●	∅	—	—	●	—	—	●	●	∅	∅	9
Edan et al. (2019)	REFOCUS-PULSAR	**—**	**—**	√	Q	●	●	●	●	∅	●	∅	∅	●	∅	—	●	—	∅	∅	∅	∅	∅	∅	7
Enticott et al. (2021)	REFOCUS-PULSAR	**—**	**—**	**—**	√	●	●	●	●	∅	●	●	∅	∅	∅	—	—	●	—	—	●	∅	∅	∅	8
Kehoe et al. (2023)	REFOCUS-PULSAR	**—**	**—**	**—**	±(Q)	●	∅	∅	∅	∅	∅	∅	∅	●	∅	∅	∅	∅	∅	∅	∅	∅	∅	∅	2
Repique et al.(2016)	SAMHSA	×	×	**—**	√	●	●	∅	●	∅	∅	●	∅	●	∅	●	—	—	●	—	—	∅	∅	∅	7
Mak et al. (2019)	BRPP	**—**	√	**—**	±	●	●	∅	●	∅	●	∅	∅	●	∅	—	●	—	—	●	—	∅	∅	∅	7
Tsai et al. (2011)	RRT	**—**	√	**—**	√	●	∅	∅	∅	∅	●	∅	∅	●	∅	—	●	—	∅	∅	∅	∅	∅	∅	4
Felton et al. (2006)	ACT	×	**—**	**—**	**—**	●	●	●	●	∅	●	∅	∅	●	∅	∅	∅	∅	∅	∅	∅	∅	∅	∅	6
Tsai et al. (2010)	GIT + SPST	**—**	√	**—**	×	●	●	●	●	∅	●	∅	∅	∅	∅	—	●	—	∅	∅	∅	∅	∅	∅	6
Hornik‐Lurie et. al. (2018)	ROTP	**—**	±	**—**	**—**	●	∅	∅	●	∅	●	∅	∅	●	∅	—	—	●	—	—	●	∅	∅	∅	6
Daley et al. (2020)	OARI	**—**	√	**—**	Q	●	●	●	●	∅	●	∅	∅	●	∅	—	●	—	—	●	—	∅	∅	∅	8
Giusti et al. (2022)	PRTP + FPTP	**—**	√	**—**	**—**	●	●	●	●	∅	●	∅	∅	●	∅	●	—	—	∅	∅	∅	∅	∅	∅	7
Walsh et al. (2017)	RBT	**—**	√	**—**	**—**	●	∅	∅	●	∅	●	∅	∅	∅	∅	●	—	—	∅	∅	∅	∅	∅	∅	4
Nardella et al. (2021)	RTP + MHP	**—**	√	**—**	**—**	●	∅	∅	●	∅	●	●	∅	●	∅	—	—	●	∅	∅	∅	●	●	∅	8
Young et al. (2005)	CISSS	**—**	√	**—**	Q	●	∅	●	∅	∅	●	∅	∅	●	∅	—	—	●	—	—	●	∅	∅	∅	6
Okamoto et al. (2018)	EBP	**—**	±	**—**	Q	●	∅	∅	●	∅	●	∅	∅	●	∅	—	●	—	—	—	●	∅	●	∅	7
Deane et al. (2014)	SAC + TC	**—**	**—**	**—**	√	●	●	∅	∅	∅	●	∅	∅	●	∅	—	—	●	—	—	●	∅	∅	∅	6
Total		0(2*)	11(3*)	5	5(4*)	30	14	11	17	1	18	3	1	22	0	3	13	9	1	8	10	3	3	0	

**Note**: √ = effective, × = not effective, ● = reported, ∅ = not reported, — = not applicable, ± = partial effect*, ACT = Assertive Community Treatment, BRPP = Brief Recovery Psychoeducation Programme,

CISSS = Consumer-led intervention/Staff Supporting Skills for Self-Help, CRTP = Collaborative Recovery Training Programme, D = day, EBP = Experience-based programme for understanding the concept of recovery,

FPTP = Family Psychoeducational Training Programme, FTF = face to face, GIT = General/inspirational training, HCP = healthcare professionals, Ix = intervention, MH = mental health service, MHP = Mental Health Passport, OARI = The Older Adults Recovery Intervention, On = online/webinar/archive, PRTP = Personal Recovery Training Programme, PSW = peer support worker, PwL = people with lived experience of mental illness,

Q = qualitative data, RBT = Recovery-based Training, ROTNP = Recovery-oriented Training Nursing Programme, ROTP = Recovery-oriented Training Programme, RRT = Recovery-related Training,

RTP = Recovery Training Programme, S = session, SAC = Skills acquisition coaching, SPST = Specific/practical skills training, SS = substance service, TC = Transformational Coaching, Tel = telephone, W = week.

### 4.3 Barriers and enablers to implementing recovery-oriented trainings

Indeed, the implementation of recovery-oriented training interventions in mental health services is challenging. Staff lack of time and high caseloads can hinder their motivation to attend training sessions and implement recovery-oriented practices in their work. With most programmes lasting between 2–7 days and of high intensity (more than 4 sessions) (Table 4), there are significant challenges to releasing clinical staff from their other responsibilities to attend training. Implementing recovery-oriented training for the healthcare workforce, including professionals and peer support workers, is part of a broader programme of organisational change. 'Organisational commitment’ is key and involves establishing a recovery vision, creating workplace support structures, implementing quality improvement initiatives, developing care pathways, and planning the workforce to support recovery (Le Boutillier et al., 2011).

In addition to organisational buy-in, the success of implementation will vary across different healthcare settings. For example, researchers in our review suggested patients with severe and acute mental illness may struggle to engage effectively in discussions about recovery with staff. It is important to address this issue by developing training that equips practitioners with the knowledge and skills to effectively engage with severely unwell patients and to find ways to support their recovery. This can be done by consulting with health professionals, service users, and other stakeholders to determine the most suitable content, format, duration, teaching methods, and innovative training techniques prior to implementation, to ensure the best fit for their service.

### 4.4 Strengths and limitations

We are the first to identify a range of categories for recovery-oriented training interventions, supporting both mental health research and practice. We have offered a detailed analysis of quantitative, qualitative, and mixed method evaluations of recovery-oriented training interventions, utilising the TIDieR checklist and the Kirkpatrick training evaluation model to assess their effects. Additionally, we have addressed gaps in current evidence by examining the field of substance misuse services and by placing a greater emphasis on peer support workers as intervention users, alongside healthcare professionals in mental health settings. The inclusion of diverse research designs (qualitative, quantitative, mixed-methods, and review studies) and examination of both English and Thai databases, as well as grey literature archives, make this review more robust. Furthermore, the use of forward and backward citation searching in our strategy reinforced our confidence that no relevant studies were overlooked.

Although the diverse study designs included provide a comprehensive understanding of recovery-oriented training interventions, the heterogeneity of these studies may limit the generalisability of the findings. The use of non-specific mental health condition terms may also limit the identification of training programmes targeting specific conditions that have been described as recovery-focused. Additionally, most of the quantitative studies evaluating the interventions' effectiveness were non-randomised controlled trials, which do not represent the gold standard for effectiveness evaluation. Of the three randomised controlled trials, only one was assessed as having a low risk of bias, whilst the other two were rated as having some concerns and a high risk of bias, due to issues related to allocation concealment. Therefore, the effectiveness findings from this review should be considered with caution.

### 4.5 Implications for future research and practice

To strengthen the evidence on the effectiveness of recovery-oriented training, future researchers should focus on conducting randomised controlled trials that ensure proper allocation concealment to minimise risk of bias. Additionally, stepped-wedge designs should be considered to ensure that all participant groups eventually receive the recovery training intervention. This might help to increase participants’ awareness, motivation, and ability to implement recovery-oriented practices in their settings. Since most evidence focuses on the immediate effects of training, there is a clear need for future researchers to assess the long-term sustainability of recovery-oriented training outcomes on professional practice and service delivery.

Given the organisational barriers to implementing recovery-oriented training, it is important to consider how interventions can be tailored to meet the needs of different services internationally. Indeed, REFOCUS is the only intervention identified that has been successfully adapted and implemented in other countries; e.g., REFOCUS-PULSAR in Australia and RETAFORM in France. The dominance of research from the USA, UK, and Australia, alongside the lack of data on non-white participants, also highlights the need to understand how to optimise recovery interventions for under-researched populations and for those in low to middle income countries.

Recovery-oriented programmes need to significantly enhance and extend professionals understanding in order to ensure that they perceive training as worthwhile. Interventions should include practical examples and interactive exercises to increase salience and strengthen connections between theory and practice. Demonstrating the possible applications of recovery-oriented principles in real-world scenarios, such as within acute or emergency services, will encourage a holistic approach to recovery among staff. At an organisational level, a better understanding of the local factors that facilitate or obstruct training implementation across settings will help to increase the feasibility of embedding a successful intervention into practice. The Structured Assessment of FEasibility (SAFE) tool (Bird et al., 2014), which evaluates the feasibility of implementing complex interventions in mental health settings through identifying barriers and enablers, can be used to support the selection of appropriate interventions that fit the needs of a particular service setting.

## 5 Conclusion

We suggest that recovery-oriented training interventions have the potential to improve recovery knowledge and attitudes among healthcare professionals and thus improve patients’ recovery outcomes. To address implementation challenges, the content and format of the training interventions need to be tailored to the context and audience by adjusting the intensity and duration to better suit the needs of the participants or organisations.

## Funding sources

No external funding

## CRediT authorship contribution statement

**Natthapon Inta:** Writing – review & editing, Writing – original draft, Project administration, Methodology, Investigation, Formal analysis, Data curation, Conceptualization. **Mary Leamy:** Writing – review & editing, Validation, Supervision, Methodology, Formal analysis, Conceptualization. **Annmarie Grealish:** Writing – review & editing, Writing – original draft, Supervision, Methodology, Investigation, Formal analysis, Conceptualization.

## Declaration of competing interest

The authors declare that they have no known competing financial interests or personal relationships that could have appeared to influence the work reported in this paper.

## Appendix Supplementary materials

Image, application 1

Image, application 2

Image, application 3

Image, application 3
