# Supplementary material for: Recovery-oriented mental health training interventions: An integrative review
Source: Int J Nurs Stud Adv. 2026 Feb 15;10:100510. doi: 10.1016/j.ijnsa.2026.100510 (PMC13080650; doi:10.1016/j.ijnsa.2026.100510)
Supplement: Supplementary file 1 [file mmc1.docx]

**Supplementary material file 1: Search terms**

| **Concept 1: Population 1 (Healthcare professionals and peer support workers)** |
| --- |
| Health Care Professionals OR Healthcare professional* OR Healthcare provider* OR health p* OR health personnel OR health care personnel OR healthcare practitioner OR healthcare p* OR Mental health professional* OR mental health personnel OR Nurse* OR general practi* OR doctor* OR physician* Or gp* OR physician* OR allied health p* OR Medical practitioner OR Peer support worker* OR peer group OR social support OR psychiatrist* OR psychologist* OR social work* OR primary healthcare p* OR DE "Psychiatric Social Workers" OR DE "Therapists" OR DE "Mental Health Personnel" OR DE "Physicians" OR DE "Clinicians" OR DE "Psychiatric Hospital Staff" OR DE "Psychologists" OR DE "Psychotherapists" OR MM "Psychiatric Nurses" |

**AND**

| **Concept 2: Population 2 (Mental and substance misuse service)** |
| --- |
| mental health* OR mental* ill* OR mental disorder* OR mental disease* OR mental problem* OR psychiatr* OR DE "Mental Disorders" OR DE "Mental Health" OR DE "Serious Mental Illness" |

**AND**

| **Concept 3: Intervention (Recovery-oriented)** |
| --- |
| recover* OR recover$ OR wellbeing OR “personal recovery” OR mental health recovery OR psychiatric adj1 recovery OR recovery-focused OR recovery focus* OR recovery-oriented OR recovery orient* OR recovery program* OR psychiatric rehabilitation OR mental health rehabilitation* OR psychological rehabilitation* OR CHIME OR CHIME framework OR empowerment* OR DE "Psychosocial Rehabilitation" OR DE "Recovery (Disorders)" OR (MM "Mental Health Recovery") |
