# Supplementary material for: Recovery-oriented mental health training interventions: An integrative review
Source: Int J Nurs Stud Adv. 2026 Feb 15;10:100510. doi: 10.1016/j.ijnsa.2026.100510 (PMC13080650; doi:10.1016/j.ijnsa.2026.100510)
Supplement: Supplementary file 2 [file mmc2.docx]

**Supplementary material file 2: Quality assessment**


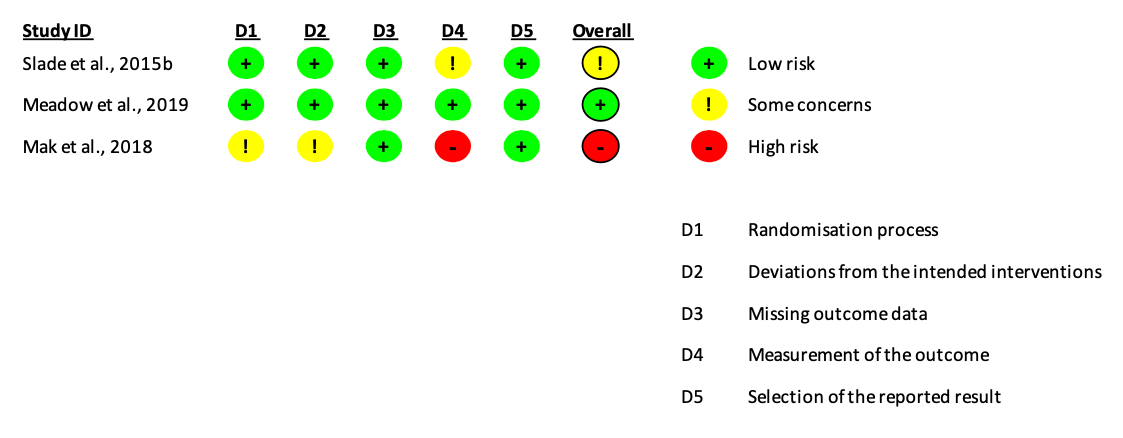
1.1. Randomised controlled trial study using Version 2 of the Cochrane Risk of Bias (ROB2) tool for assessing the risk of bias

1.2. Quasi-experimental study using JBI checklist

| **Joanna Briggs Institute: Quasi-Experimental Studies** | | Young et al, 2005 | Crowe et al., 2006 | Salgado et al., 2010 | Wilrycx et al., 2012 | Deane et al., 2014 | Wilrycx et al., 2015 | Walsh et al., 2017 | Zuaboni et al., 2017 | Enticott et al., 2021 | Giusti et al., 2022 | Williamson et al, 2023 |
| --- | --- | --- | --- | --- | --- | --- | --- | --- | --- | --- | --- | --- |
| 1 | Is it clear in the study what is the ‘cause’ and what is the ‘effect’ (i.e. there is no confusion about which variable comes first)? | Yes | Yes | Yes | Yes | Yes | Yes | Yes | Yes | Yes | Yes | Yes |
| 2 | Were the participants included in any comparisons similar? | Yes | Yes | Yes | Yes | Yes | Yes | Yes | Yes | Yes | Yes | N/A |
| 3 | Were the participants included in any comparisons receiving similar treatment/care, other than the exposure or intervention of interest? | Yes | Yes | Yes | Yes | Yes | Yes | Yes | Yes | Yes | Yes | Yes |
| 4 | Was there a control group? | Yes | Yes | N/A | Unclear | Yes | No | No | Yes | No | Yes | NO |
| 5 | Were there multiple measurements of the outcome both pre and post the intervention/exposure? | Yes | Yes | Yes | Yes | Yes | Yes | Yes | Yes | Yes | Yes | Yes |
| 6 | Was follow up complete and if not, were differences between groups in terms of their follow up adequately described and analyzed? | Yes | Yes | Yes | N/A | Yes | Yes | Yes | Yes | Yes | Yes | Yes |
| 7 | Were the outcomes of participants included in any comparisons measured in the same way? | Yes | Yes | Yes | Yes | Yes | Yes | Yes | Yes | Yes | Yes | Yes |
| 8 | Were outcomes measured in a reliable way? | Yes | Yes | Yes | Yes | Yes | Yes | Yes | Yes | Yes | Yes | Yes |
| 9 | Was appropriate statistical analysis used? | Yes | Yes | Yes | Yes | Yes | Yes | Yes | Yes | Yes | Yes | Yes |

1.3. Cross-sectional study using JBI checklist

| **Joanna Briggs Institute: Analytical Cross Sectional Studies** | | Uppal et al., 2010 | Tsai et al., 2010 | Tsai et al., 2011 |
| --- | --- | --- | --- | --- |
| 1 | Were the criteria for inclusion in the sample clearly defined? | Yes | Yes | Yes |
| 2 | Were the study subjects and the setting described in detail? | Yes | Yes | Yes |
| 3 | Was the exposure measured in a valid and reliable way? | Yes | Yes | Yes |
| 4 | Were objective, standard criteria used for measurement of the condition? | Yes | Yes | Yes |
| 5 | Were confounding factors identified? | Yes | Yes | Yes |
| 6 | Were strategies to deal with confounding factors stated? | Yes | Yes | Yes |
| 7 | Were the outcomes measured in a valid and reliable way? | Yes | Yes | Yes |
| 8 | Was appropriate statistical analysis used? | Yes | Yes | Yes |

1.4. Mixed Methods study using Mixed Methods Appraisal Tool (MMAT) checklist

| **Mixed Methods Appraisal Tool (MMAT, 2018)** | | | Repique et al., 2016 | Hornik‐Lurie et. al., 2018 | Okamoto et al., 2018 | Daley et al., 2020 | Nardella et al., 2021 |
| --- | --- | --- | --- | --- | --- | --- | --- |
| Screening Questions  (for all types) | S1 | Are there clear research questions? | Yes | Yes | Yes | Yes | Yes |
|  | S2 | Do the collected data allow to address the research questions? | Yes | Yes | Yes | Yes | Yes |
| 1. Qualitative | 1.1 | Is the qualitative approach appropriate to answer the research question? | Yes | Yes | Yes | Yes | Yes |
|  | 1.2 | Are the qualitative data collection methods adequate to address the research question? | Yes | Yes | Yes | Yes | Yes |
|  | 1.3 | Are the findings adequately derived from the data? | Yes | Yes | Yes | Yes | Yes |
|  | 1.4 | Is the interpretation of results sufficiently substantiated by data? | Yes | Yes | Yes | Yes | Yes |
|  | 1.5 | Is there coherence between qualitative data sources, collection, analysis and interpretation? | Yes | Yes | Yes | Yes | Yes |
| 2. Quantitative randomized controlled trials | 2.1 | Is randomization appropriately performed? |  |  |  |  |  |
|  | 2.2 | Are the groups comparable at baseline? |  |  |  |  |  |
|  | 2.3 | Are there complete outcome data? |  |  |  |  |  |
|  | 2.4 | Are outcome assessors blinded to the intervention provided? |  |  |  |  |  |
|  | 2.5 | Did the participants adhere to the assigned interevntion? |  |  |  |  |  |
| 3. Quantitative non- randomized | 3.1 | Are the participants representative of the target population? | Yes | Yes | Yes | Yes | Yes |
|  | 3.2 | Are measurements appropriate regarding both the outcome and intervention (or exposure)? | Yes | Yes | Yes | Yes | Yes |
|  | 3.3 | Are there complete outcome data? | Yes | Yes | Yes | Yes | Yes |
|  | 3.4 | Are the confounders accounted for in the design and analysis? | Yes | Yes | Yes | Yes | Yes |
|  | 3.5 | During the study period, is the intervention administered (or exposure occurred) as intended? | Yes | Yes | Yes | Yes | Yes |
| 4. Quantitative descriptive | 4.1 | Is the sampling strategy relevant to address the research question? |  |  |  |  |  |
|  | 4.2 | Is the sample representative of the target population? |  |  |  |  |  |
|  | 4.3 | Are the measurements appropriate? |  |  |  |  |  |
|  | 4.4 | Is the risk of nonresponse bias low? |  |  |  |  |  |
|  | 4.5 | Is the statistical analysis appropriate to answer the research question? |  |  |  |  |  |
| 5. Mixed methods | 5.1 | Is there an adequate rationale for using a mixed methods design to address the research question? | Yes | Yes | Yes | Yes | Yes |
|  | 5.2 | Are the different components of the study effectively integrated to answer the research question? | Yes | Yes | Yes | Yes | Yes |
|  | 5.3 | Are the outputs of the integration of qualitative and quantitative components adequately interpreted? | Yes | Yes | Yes | Yes | Yes |
|  | 5.4 | Are divergences and inconsistencies between quantitative and qualitative results adequately addressed? | Yes | Yes | Yes | Yes | Yes |
|  | 5.5 | Do the different components of the study adhere to the quality criteria of each tradition of the methods involved? | Yes | Yes | Yes | Yes | Yes |

1.5. Qualitative study using JBI checklist

| **Joanna Briggs Institute: Qualitative Research Checklist** | | Wallace et al., 2016 | Clarke et al., 2020 | Leamy et al., 2014 | Edan et al., 2019 | Kehoe et al., 2023 | Felton et al., 2006 |
| --- | --- | --- | --- | --- | --- | --- | --- |
| 1 | Is there congruity between the stated philosophical perspective and the research methodology? | Unclear | Unclear | Unclear | Unclear | Unclear | Unclear |
| 2 | Is there congruity between the research methodology and the research question or objectives? | Yes | Yes | Yes | Yes | Yes | Unclear |
| 3 | Is there congruity between the research methodology and the methods used to collect data? | Yes | Yes | Yes | Yes | Yes | Yes |
| 4 | Is there congruity between the research methodology and the representation and analysis of data? | Yes | Yes | Yes | Yes | Yes | Yes |
| 5 | Is there congruity between the research methodology and the interpretation of results? | Yes | Yes | Yes | Yes | Yes | Yes |
| 6 | Is there a statement locating the researcher culturally or theoretically? | No | No | No | No | No | No |
| 7 | Is the influence of the researcher on the research, and vice- versa, addressed? | Yes | No | No | No | No | No |
| 8 | Are participants, and their voices, adequately represented? | Yes | Yes | Yes | Yes | Yes | Yes |
| 9 | Is the research ethical according to current criteria or, for recent studies, and is there evidence of ethical approval by an appropriate body? | Yes | Yes | Yes | Yes | Yes | Unclear |
| 10 | Do the conclusions drawn in the research report flow from the analysis, or interpretation, of the data? | Yes | Yes | Yes | Yes | Yes | Yes |

1.6. Systematic review study using JBI checklist

| **Joanna Briggs Institute: Systematic Review and Research Synthesis** | | Jackson-Blott et al, 2019 | Eiroa-Orosa et al., 2019 | Hawsawi et al., 2021 |
| --- | --- | --- | --- | --- |
| 1 | Is the review question clearly and explicitly stated? | No | Yes | Yes |
| 2 | Were the inclusion criteria appropriate for the review question? | Yes | Yes | Yes |
| 3 | Was the search strategy appropriate? | Yes | Yes | Yes |
| 4 | Were the sources and resources used to search for studies adequate? | Yes | Yes | Yes |
| 5 | Were the criteria for appraising studies appropriate? | Yes | Yes | Yes |
| 6 | Was critical appraisal conducted by two or more reviewers independently? | Yes | Yes | Yes |
| 7 | Were there methods to minimize errors in data extraction? | Yes | Yes | Yes |
| 8 | Were the methods used to combine studies appropriate? | Yes | Yes | Yes |
| 9 | Was the likelihood of publication bias assessed? | Yes | Yes | Yes |
| 10 | Were recommendations for policy and/or practice supported by the reported data? | Yes | Yes | Yes |
| 11 | Were the specific directives for new research appropriate? | Yes | Yes | Yes |
