# Supplementary material for: Recovery-oriented mental health training interventions: An integrative review
Source: Int J Nurs Stud Adv. 2026 Feb 15;10:100510. doi: 10.1016/j.ijnsa.2026.100510 (PMC13080650; doi:10.1016/j.ijnsa.2026.100510)
Supplement: Supplementary file 3 [file mmc3.docx]

**Supplementary material file 3:**

**Recovery-oriented training intervention categories**

The synthesis of characteristics and nature of recovery-oriented training interventions described in this study was categorised utilising the TIDieR checklist. Seven categories of recovery-oriented training programmes were identified based on their primary attributes and underpinning theoretical frameworks: CRM-based; CHIME-based; SAMHSA-based; IMR-based; Bedregal’s personal recovery model based; multiple recovery theories based; and other theory based.

# **CRM-based (Collaborative Recovery Model)**

## Collaborative Recovery Training Programme (CRTP)

Four studies (Crowe et al., 2006, Oades et al., 2005, Salgado et al., 2010, Uppal et al., 2010) examined the Collaborative Recovery Training Programme (CRTP), developed and implemented in community mental health and rehabilitation services in Australia. The CRTP is designed to integrate motivational enhancement with collaborative goal setting, emphasising autonomy, hope, and individual experience as core principles of the recovery movement in mental health (Crowe et al., 2006, Salgado et al., 2010). The training equips professionals with concepts and practical skills to support consumers in setting, pursuing, and achieving their personal recovery goals. The programme consists of a two-day workshop, followed by one-day booster sessions at six and twelve months (Oades et al., 2005, Uppal et al., 2010).

## The CRM staff development programme

One study (Williamson et al., 2023) evaluated the Collaborative Recovery Model (CRM) staff development programme, which was designed and implemented for mental health professionals in Australian mental health services. The CRM training emphasises well-being and resilience, guided by two core principles: recognising recovery as an individual journey and promoting collaboration and autonomy. The programme comprises four main components: change enhancement, collaborative strengths and values identification, collaborative visioning and goal striving, and collaborative action planning and monitoring. Training is delivered over two days by certified CRM trainers, who complete a comprehensive credentialing process that includes a five-day initial training, a three-day train-the-trainer programme, three days of practice facilitation, and ongoing booster sessions and coaching at six and twelve months after the initial training.

# **CHIME-based (Connectedness-Hope-Identity-Meaning-Empowerment)**

## REFOCUS

Five studies (Clarke et al., 2020, Leamy et al., 2011, Slade et al., 2015b, Slade et al., 2015a, Wallace et al., 2016) examined the REFOCUS programme, a team-level intervention designed to enhance staff behaviour by increasing attention to the values, preferences, strengths, and goals of people with psychosis, as well as by strengthening staff–patient relationships through coaching and partnership. The programme was developed and implemented within UK community mental health services for community-based personnel. REFOCUS is grounded in the CHIME framework, which is widely recognised as one of the most robust models of personal recovery (Leamy et al., 2011).

The intervention included both in-person and telephone consultations as needed and comprised two main components: (1) fostering recovery-oriented relationships, which involved building a shared team understanding of personal recovery, exploring individual and team values, providing coaching skills training, engaging teams in collaborative projects with service users, and enhancing service users’ expectations that their values, strengths, and goals would be prioritised; and (2) adapting working practices to align with service users’ treatment preferences, strengths, and personally valued goals. Staff received structured training and support, including 12 hours of instruction in personal recovery (delivered in three 4-hour sessions) by two trainers, and 16 hours of recovery coaching (one 8-hour session and two 4-hour sessions) by a coaching trainer, supplemented by telephone assistance and optional booster sessions.

## REFOCUS-PULSAR

Four studies (Edan et al., 2019, Enticott et al., 2021, Kehoe et al., 2023, Meadows et al., 2019) examined REFOCUS-PULSAR, a staff training intervention adapted for Australian clinical and community mental health service contexts. The programme comprises two parts. Part 1 includes: (1) a core module (3.5 hours) focused on implementing recovery-oriented training in general practices and deepening understanding of consumer and carer perspectives in mental healthcare, and (2) a clinical enhancement module (4 hours) aimed at developing skills in detecting and assessing schizophrenia, applying recovery principles to treatment planning and monitoring, creating recovery-focused treatment plans, and understanding review processes and relapse prevention strategies. Part 2 provides an optional active learning component through “PALS,” a monthly one-hour online session facilitated by a consultant specialist psychiatrist, where participants review, reflect on, and share their experiences in implementing the programme while receiving support from the training team.

The face-to-face training sessions were co-delivered using both didactic teaching and experiential learning, supported by a comprehensive manual and other resources. Training was led by professional staff and trainers with personal experience of mental health challenges, together with the project’s consumer researcher. It consisted of two separate two-day sessions—one for clinical services and another for community services—delivered within the same week.

## Other Recovery-oriented Training Programme and Recovery-oriented Nursing Training Programme

Two studies (Wilrycx et al., 2015, Wilrycx et al., 2012) investigated a recovery-focused training programme in the Netherlands. This intervention, delivered by an expert with experience in both peer support and professional rehabilitation teaching, consisted of two seminars held every six months over a two-day period. The seminars were divided into two modules: the first introduced professionals to the fundamentals of recovery-oriented care, while the second emphasised cultivating a recovery-oriented attitude among participants.

Another study (Zuaboni et al., 2017) examined a recovery-oriented nursing training programme for mental health nurses at two psychiatric hospitals in Switzerland. The programme comprised five half-day sessions: the first focused on personal recovery and social inclusion using REFOCUS training material; the second and third covered the basics of motivational interviewing; the fourth addressed goal attainment strategies and goal attainment scaling; and the fifth concentrated on integrating training content into care processes, documentation, and interprofessional collaboration. Although mental health nurses initially demonstrated strong motivation to participate, over time the procedures were regarded as overly ambitious and difficult to manage alongside demanding daily ward routines.

# **SAMHSA-based (Substance Abuse and Mental Health Services Administration)**

## The SAMHSA training programme

One study (Repique et al., 2016) examined the SAMHSA training programme, which includes patient engagement models, trauma systems theory, and strategies for reducing restraint. The intervention consisted of a one-hour training delivered via an archived online webinar for all hospital psychiatric and mental health nurses across shifts in the hospital auditorium in the USA. It was conducted by an interdisciplinary team of experts in mental health recovery research and practice.

## The Brief Recovery Psychoeducation Programme

One study (Mak et al., 2019) assessed the Brief Recovery Psychoeducation Programme, which addresses key aspects of recovery, compares medical and rehabilitation models with consumer-oriented recovery, explores the perspectives of people with mental illness and family carers, discusses existing good practices in recovery-oriented care, and considers potential challenges and dilemmas. The programme consists of a two-day psychoeducation course (three hours per day) for service providers in community-based psychiatric rehabilitation services in Hong Kong. It is facilitated by senior social workers with extensive experience supporting individuals with mental illness. The training employs a variety of methods, including didactic teaching, interactive games and videos, discussions, presentations, and quizzes, to achieve its learning objectives.

## General/inspirational training

One study (Tsai et al., 2010) evaluated a general/inspirational training programme designed to prompt staff reflection on their practices. The training encouraged staff to use planning rooms as calming spaces for clients to alleviate stress, thereby reducing the need for restraint and seclusion. Key components included de-escalation techniques and the use of the least restrictive methods. The programme was delivered as a three-day workshop, with an additional one-day presentation, led by a former service user and a renowned speaker on recovery in healthcare.

# **IMR-based (Illness Management and Recovery) and Bedregal’s personal recovery model based**

One study (Tsai et al., 2011) explored recovery-related training for community health staff, consisting of a two-day training session on Illness Management and Recovery (IMR). IMR is a recovery framework that helps service users set and achieve personal recovery goals, gain knowledge, and independently manage their illnesses. This was followed by a one-day IMR case consultation workshop.

Another study (Giusti et al., 2022) investigated the Personal Recovery Training Programme, based on Bedregal et al.’s (2006) personal recovery model. The training covered mental health outcomes, principles of personal recovery, and group activities designed to strengthen knowledge, enhance recovery-oriented practice, and support implementation. This one-day, eight-hour programme was delivered by mental health university staff and National Health System experts, with two service users contributing as teachers and tutors. It targeted all mental health professionals in the Italian Psychiatric University Unit and used a mix of lectures, discussions, and group work facilitated by a tutor.

# **Multiple recovery theories based**

## Core Assertive Community Treatment (ACT)

One study (Felton et al., 2006) explored the Core Assertive Community Treatment (ACT) intervention, which incorporated Wellness Recovery Action Plans (WRAP) to support staff in engaging clients by helping them develop individualised recovery plans, alongside the Illness Management and Recovery (IMR) framework. A dedicated recovery module was introduced, covering models of recovery and practices such as Deegan’s (2000) Key Elements in Supporting Recovery, recovery-centred service planning, and WRAP planning (Copeland, 2002). The programme was delivered in a classroom setting and included face-to-face presentations by nationally recognised experts in recovery and illness self-management, as well as local peer and non-peer specialists, for ACT team members across all disciplines at the New York State ACT Institute.

## Specific/practical skills training and Recovery-oriented training

One study (Tsai et al., 2010) examined a specific/practical skills training programme grounded in Illness Management and Recovery (IMR), Wellness Recovery Action Plans (WRAP), Integrated Dual Disorders Treatment, the Matrix model, and Motivational Interviewing. IMR helps service users set and achieve personal recovery goals, acquire knowledge, and independently manage their illnesses. WRAP supports staff in engaging clients by assisting them in developing individualised recovery plans. Integrated Dual Disorders Treatment equips staff to provide combined mental health and substance use interventions tailored to clients’ treatment stage and readiness to change. The Matrix model offers a structured approach to substance use treatment, including relapse prevention techniques, while Motivational Interviewing encourages staff to use clients’ motivations and resources to facilitate behaviour change.

Hornik-Lurie et al. (2018) introduced a recovery-oriented training intervention comprising Illness Management and Recovery (IMR), peer support, and psychiatric advance directives training. IMR focused on staff training to foster personal relationships, emphasise positive aspects, and instil trust and hope in achieving personal goals. The peer support component involved ten peer support workers and a supervisor across different wards, participating in multidisciplinary team activities and delivering individual and group interventions. Psychiatric advance directives training enabled individuals receiving psychiatric care to state their preferences and directives in advance, safeguarding their dignity and treatment choices. These one-hour sessions, delivered individually or in groups, were conducted weekly for mental health personnel in Israel over periods ranging from one to eleven months.

# **Other theory based**

## The Older Adults Recovery Intervention (OARI)

One study (Daley et al., 2020) evaluated the Older Adults Recovery Intervention (OARI), a manualised educational programme designed to enhance staff pro-recovery practices. Developed from previous qualitative research and recovery literature, the intervention targets clinical teams in older people’s mental health services in London. It consists of three one-day modules—Promoting Recovery, Maintaining Identity, and Enhancing Resilience—delivered sequentially with homework between modules. Each module combines didactic teaching on recovery-oriented practice with practical exercises, allowing staff to share clinical experiences and discuss how to integrate training content into routine practice. Following the modules, an action planning day supports the development of a team recovery action plan with specific objectives. Implementation support, provided by the OARI trainer for up to six months after the action planning day, includes guidance on introducing new pro-recovery team processes and educational supervision at both team and individual levels.

## Consumer-led intervention, Staff Supporting Skills for Self-Help

One study (Young et al., 2005) evaluated the Consumer-led Staff Supporting Skills for Self-Help intervention, grounded in an emerging national movement of consumers with severe mental illness and implemented in community mental health settings in Arizona and Colorado, United States. The intervention comprises five group components delivered over one year, with staff attending 16-hour meetings at various points during the study. Training methods include didactic education, small group discussions, role play, clinician-service user dialogues, and individual advice.

The programme begins with scientific presentations on self-help, assessing clinicians’ prior support of self-help, followed by structured small-group dialogues with equal numbers of consumers and clinicians to discuss barriers and share experiences that foster hope. Rehabilitation readiness sessions equip clinicians with concepts and skills to help consumers set goals, develop coping strategies, and manage their own stressors, consumer demands, and the broader mental health system. Strategies for independence sessions focus on reducing consumer dependence on mental health professionals, while professional skills supporting self-help sessions use small groups and role-playing techniques to teach staff how to support self-help without being intrusive.

## The experience-based recovery-oriented training programme

One study (Okamoto and Tanigaki, 2018) established an experience-based programme to help nurses in Japanese psychiatric wards understand the concept of recovery. The intervention utilised ACT observational practice over a three-day training period, comprising five hours of lectures, nine hours of ACT observational practice, and two hours of group work.

On the first day, a 90-minute lecture and group work session focused on rethinking understanding of families, providing family support, and clarifying goals. Another 90-minute lecture addressed ACT support in private medical facilities, including attitudes towards recovery and outreach/community care. A 30-minute survey report presentation discussed insights from families who are ACT service users. On the second day, 540 minutes of observational practice allowed participants to experience ACT and provide visiting support to service users, followed by feedback from staff. The final day included a 120-minute group work session, a question-and-answer session on experience presentations and clinical cases, and a 90-minute lecture on hospital–community coordination and the fundamentals of community living.

Walsh et al. (2017) conducted a recovery-oriented training programme for health professionals in Ireland, guided by adult education principles and reflective practice. The four-hour workshop covered defining recovery, exploring its principles, and applying them in clinical settings. Facilitators—including service users, family members, and providers—shared their experiences during reflective sessions.

**Table 1: Summary of recovery-oriented trainings according to the TIDieR checklist (n = 30)**

| **Author (year)** | **Country** | **Brief name** | **Why** | **What** | **Who** | | **How** | **Where** | **When and how much** | **Modification** | **How well** |
| --- | --- | --- | --- | --- | --- | --- | --- | --- | --- | --- | --- |
|  |  |  |  |  | **Trainees** | **Trainers** |  |  |  |  |  |
| **Collaborative recovery model (CRM)** | | | | | | | | | | | |
| Oades et al. (2005) | Australia | Collaborative Recovery Training Program (CRTP) | CRM | 6 modules: 1) Recovery as an individual process, 2) Collaborative and autonomy support, 3) Change enhancement, 4) Collaborative needs identification, 5) Collaborative goal striving, 6) Collaborative task striving | Predominantly for clinical staff but consumer advocates are encouraged to attend | N/R | N/R | Community mental health teams, rehabilitation services and supported housing contexts | 2 days training; (1 day booster sessions at 6 and 12 months after the initial training) | N/R | N/R |
| Crowe et al. (2006) | Australia | Collaborative Recovery Training Program (CRTP) | CRM | Integrated principles and skill for example, motivational enhancement and collaborative goal setting. The program also emphasizes issues of autonomy, hope, and individual experience that are central to the recovery movement of mental health consumers. | N/R | N/R | N/R | N/R | N/R | N/R | N/R |
| Salgado et al. (2010) | Australia | Collaborative Recovery Training Program (CRTP) | CRM | Training in recovery concepts and skills supporting consumers’ abilities to set, pursue and attain personal goals. | N/R | N/R | N/R | N/R | 2 days of training | N/R | N/R |
| Uppal et al. (2010) | Australia | Collaborative Recovery Training Program (CRTP) | CRM | Training clinicians in the use of the two protocols: Collaborative Goal Technology (CGT) and Homework (HW). The training emphasizes motivation enhancement, collaborative goal setting and collaborative homework assignment, within a framework of broader recovery principles. | N/R | N/R | N/R | N/R | 2 days training workshop:  (1-day booster sessions 6 months and 12 months) | N/R | N/R |
| Williamson et al. (2023) | Australia | The CRM staff development program | CRM | Two main principles (recovery as an individual process and collaboration and autonomy support) with four key components: change enhancement, collaborative strengths and values identification, collaborative visioning and goal striving, and collaborative action planning and monitoring. | Psychiatrists, other medical staff, nurses, occupational therapists, social workers, psychologists, neuropsychologists, speech pathologists, lived-experience workforce (peer worker roles), and senior leadership and service development positions | CRM trainer credentialing included attendance at an initial training course (5 days), train-the-trainer program (3 days), practice facilitation (3 days), a booster training session at 6 months (1 day), and then annually (1 day) thereafter | N/R | Mental health services for infants, children, youths, adults, and older persons, community care teams, inpatient units, assertive outreach and crisis assessment teams, consultation-liaison services, subacute and residential services | 2 days training; (1 day booster sessions and coaching at 6 and 12 months after the initial training) | N/R | N/R |
| **REFOCUS** | | | | | | | | | | | |
| Slade et al. (2015a) | UK | REFOCUS | CHIME framework | Comprising of two components:  (i) Recovery-promoting relationships  1) coaching skills training for staff  2) developing a shared team understanding of recovery  3) exploring staff values  4) a Partnership Project with people who use the service and raising patient expectations  (ii) Working practices  1) Understanding values and treatment preferences  2) Strengths assessment  3) Supporting goal-striving  The REFOCUS programme placed a particular emphasis on supporting recovery for Black people, who in England are a minority ethnic group with high psychosis prevalence and problematic pathways to care | N/R | N/R | N/R | N/R | N/R | N/R | N/R |
| Slade et al. (2015b) | UK | REFOCUS | CHIME framework | The intervention has behavioural and interpersonal components:  1) The behavioural components comprising of 3 desired behaviours by staff called “working practices"  i) working practice 1: understanding patients’ values and identity beyond being a patient and placing patients’ preferences at the centre of planning care  ii) working practice 2: assessing patients’ personal and social strengths with a standard approach to identify existing and potential resources on which the patient can build  iii) working practice 3: supporting patients in striving for goals by orienting clinical care around goals valued by the patient  2) Interpersonal components called recovery-promoting relationships | GP, Care coordinator, Psychiatrist, other doctor, Psychologist, Social worker, Nurse, OT, Support worker, Vocational worker, Drug and alcohol, adviser, other therapist Specialist team, Day care, | Two trainers (one with a professional background and one with a service-use background) | In-person and telephone consultation | Community-based adult mental health (SlaM in London and 2gether in Gloucestershire) | Training and support meetings were offered to staff: 12 h (three 4 h sessions) of training in personal recovery provided by two trainers. 16 h (one 8 h and two 4 h sessions) of training in recovery coaching from a coaching trainer, with telephone support and optional booster sessions | Originally 18 months training. Due to difficulty in recruiting patients, the 1-year time frame was implemented. | N/R |
| Wallace et al. (2016) | UK | REFOCUS | CHIME framework | The intervention consisted of two components:  1) Pro-recovery Working Practices: provided staffs with materials to support: Understanding values and treatment preferences (WP1), Assessing strengths (WP2), and Supporting goal-striving (WP3), and were designed to be used collaboratively with service users  2) Recovery-promoting relationships | N/R | N/R | N/R | Adult community mental health teams in two Trusts in England: South London and Maudsley NHS Foundation Trust (SLaM) and 2gether NHS Foundation Trust (2gether) in Gloucestershire | N/R | N/R | N/R |
| Clarke et al. (2020) | UK | REFOCUS | CHIME framework | The intervention consisted of two components:   (i) Recovery-promoting relationships (Developing a shared team understanding of personal recovery; Exploring individual and team values; Skills training in coaching; Teams carrying out partnership project with service users; Raising the expectations held by service users that their values, strengths and goals will be prioritised)  (ii) Working practices (Values and treatment preferences; Strengths; Personally- valued goals) | N/R | A professional coach and trainer from SLaM partners and person with lived experience of mental illness from the charity RETHINK mental illness | N/R | South London and Maudsley NHS trust and 2gether NHS trust | Information sessions for staff and service users: 1 hour Personal recovery training: 3 x half days Coaching conversations for Recovery training: 1 full and 2 half days Team reflection sessions: 1 hour Team leader reflection sessions: 1 hour | N/R | N/R |
| Leamy et al. (2014) | UK | REFOCUS | CHIME framework | Component 1: Recovery-promoting relationships  - Developing a shared team understanding of personal recovery  - Exploring individual and team values  - Skills training in coaching  - Teams carrying out partnership project with service users  - Raising the expectations held by service users that their values, strengths and goals will be prioritised Component 2: Working practices  - Values and treatment preferences  - Strengths  - Personally- valued goals | N/R | N/R | Personal recovery training (10.5 hours); coaching and working practice training (14.5 hours); team manager reflection sessions focussed on team culture (3 hours externally facilitated by the Personal Recovery trainer) and whole team reflection sessions (3 hours externally facilitated, 3 hours internally facilitated by team) | Community-based mental health teams in two UK sites (one urban, one semi-rural) | Personal recovery training (10.5 hours); coaching and working practice training (14.5 hours); team manager reflection sessions focussed on team culture (3 hours externally facilitated by the Personal Recovery trainer) and whole team reflection sessions (3 hours externally facilitated, 3 hours internally facilitated by team) | N/R | N/R |
| Wilrycx et al. (2012) | Netherlands | Recovery-oriented training program | REFOCUS and CHIME framework | 2 modules: The first module was focused on the basics of recovery-oriented care to familiarise the professional with the concept of recovery.  The second module was focused on the recovery-oriented attitude of the professional | N/R | Both courses were presented by an expert by experience from a peer support centre and a professional rehabilitation teacher | The educational program was given in two separate intensive training sessions, one in 2008 and a second one in 2009 | N/R | 2 days session every six months | N/R | N/R |
| Wilrycx et al. (2015) | Netherlands | Recovery-oriented care training programme | REFOCUS and CHIME framework | N/R | N/R | N/R | Seminar | N/R | Two seminars given in 2-day session every 6 months | N/R | N/R |
| Zuaboni et al. (2017) | Switzerland | Recovery-oriented nursing training programme | REFOCUS and CHIME framework | Training session 1:   Personal recovery and social inclusion based upon the REFOCUS training material Training session 2 & 3:   Basics of Motivational Interviewing Training session 4:   Goal attainment strategies and goal attainment scaling  Training session 5:   Implementation of the training contents into care process and documentation, as well as cooperation with other professional groups | Mental Health Nurses | N/R | N/R | Two psychiatric hospitals, which are contracted by the canton of Zurich | 5 half-day training sessions | N/R | At the beginning of the study, the MHNs were highly motivated to take an active part. However, over the longer term, the study procedures were seen as being too ambitious to be coordinated within the stressful daily routines on the wards. |
| **REFOCUS-PULSAR** | | | | | | | | | | | |
| Meadows et al. (2019) | Australia | REFOCUS-PULSAR | REFOCUS and CHIME framework | Step 1: intervention for clinical services was designed as a 2-day session, with the community services training planned as a separate 2-day session during the same week Step 2: training was modified according to analyses of participant and trainer feedback from step one | Multiple discipline apart from senior medical staff as they would be more likely to attend service-wide, profession-specific training | Professional staff and trainers with lived experience of mental health problems, and the project’s consumer researcher | Slide presentations, a manual, session plans, active learning session, and videos | Clinical services and community services | A 2-day session for the clinical services and a separate 2-day session for the community services during the same week. PULSAR active learning sessions, offered monthly as 1 h sessions to staff. | No PULSAR active learning sessions occurred for logistical and engagement reason in two public mental health service teams. PULSAR active learning sessions were integrated into monthly staff support session in mental health community. | N/R |
| Edan et al. (2019) | Australia | REFOCUS-PULSAR | REFOCUS and CHIME framework | Trainees were given opportunities within the training to explore the application of these principles (CHIME framework) and working practices to their own practice. All staff were provided with an opportunity to participate in team-based monthly sessions called PALS (PULSAR active learning sessions), designed to support staff implementation of the recovery-oriented practice training. | Staff of community mental health services | All training was co-facilitated by a clinician from the sector and an experienced consumer trainer | The training included didactic teaching and experiential learning, supported with a manual, tools and resources | Community mental health services | 2 days of training | N/R | N/R |
| Enticott et al. (2021) | Australia | REFOCUS-PULSAR | REFOCUS and CHIME framework | Part 1: Modular MHST Component  Module 1: Core Module (3.5 h) focusing on operationalizing ROP in general practice; and enhanced understanding of the perspective of consumers and carers in the provision of mental healthcare.  Module 2: Clinical Enhancement Module (CEM; 4 h) developing of skills in the detection and assessment of Schizophrenia; an ability to apply the principles of ROP to treatment planning and monitoring; the ability to develop recovery-focused mental health treatment plans; and an applied understanding of review processes and relapse prevention strategies for mental illness within a ROP framework.  Part 2: Optional Active Learning Sessions  Module 3: PALS (optional) is monthly 1-h online sessions called “PALS (PULSAR Active Learning Sessions)” with a consultant specialist psychiatrist to review, reflect and share their experiences in the implementation of ROP | General practitioners (GPs) | Mental health clinicians, including experienced trainers from the study team, with co-delivery by consumer trainers | Co-delivered fact-to-face training, multimedia, mnemonics, and targeted interview schedules to encourage ROP—with availability of support sessions for 1 year  Active learning sessions (optional) = monthly 1-h online sessions called “PALS (PULSAR Active Learning Sessions)” with a consultant specialist psychiatrist to review, reflect and share their experiences in the implementation of ROP | N/R | Monthly 1-h online sessions called “PALS (PULSAR Active Learning Sessions)” for 13 months | N/R | N/R |
| Kehoe et al. (2023) | Australia | REFOCUS-PULSAR | REFOCUS and CHIME framework | N/R | N/R | N/R | N/R | Publicly funded community mental health service | N/R | N/R | N/R |
| **SAMHSA recovery principles** | | | | | | | | | | | |
| Repique et al.(2016) | USA | The SAMHSA training program | SAMHSA recovery principles | The training program synthesizes the body of research in the field, which includes the following topics:  (1) patient engagement models (2) trauma systems theory (3) restraint reduction strategies (4) integration of peer-to-peer services in psychiatric treatment (5) outcomes of randomized trial of consumer-managed alternative mental health treatment programs | Psychiatric and mental health nurses | Interdisciplinary experts in mental health recovery research and practice | Archived online webinar | The trainings were held in the hospital's auditorium using a large projector screen with speakers | 1 hour-long training via archived online webinar for all hospital PMH-RNs across all shifts | N/R | N/R |
| Mak et al. (2019) | Hong Kong | Study 1: Brief Recovery Psychoeducation Program for Service Providers  Study 2: Brief Recovery Psychoeducation Program for Service users | SAMHSA recovery principles and the findings of the previous focus groups | 1) Didactic teaching:  Introduction to different aspects of recovery  Comparison of the medical and rehabilitation models of recovery with consumer-oriented recovery  Perspectives of people with mental illness and family carers  Action to be done by various parties.  Existing good practices of recovery-oriented care  Possible challenges and dilemmas  2) Interactive games / videos Sharing:  Debriefing after each activity to clarify the idea being introduced.  Service users and family carers shared their pathways of recovery.  3) Discussion:  Reflection about the understanding and meaning of recovery.  Discussion on how recovery elements can be applied in different scenarios.  Identified the potential challenges and barriers to the implementation of recovery-oriented care.  4) Presentation (only for service providers):  Presentation on the summary of group discussion regarding the adoption of recovery-oriented care for people with different backgrounds and needs  Feedback and suggestions to address the concerns about implementing recovery-oriented practices.  5) Quiz:  Short quiz to revisit recovery concepts being introduced | Service providers and service users | Senior social workers who had extensive experience working with people with mental illness at the organization | Didactic teaching, Interactive games/videos Sharing, Discussion, Presentation (only for service providers), Quiz | Community-based psychiatric rehabilitation services in Hong Kong | Study 1: - 2-day psychoeducation (3 hour/day).  - A two-session intervention with 3 hours for each session.  Study 2: - 2-week psychoeducation (2 hour/day).  - A two-session intervention with 2 hours for each session modified to a 2-week programme. | N/R | N/R |
| **Illness Management and Recovery (IMR)** | | | | | | | | | | | |
| Tsai et al. (2011) | USA | Recovery-related training | IMR | N/R | N/R | N/R | IMR case consultation workshop was provided to community health staff during the year of the study | Four community mental health centres | 2 days IMR training and 1-day IMR case consultation workshop was provided to community health staff during the year of the study | N/R | N/R |
| **Bedregal’s personal recovery model** | | | | | | | | | | | |
| \| Giusti et al. (2022) \| Italy \| Personal Recovery Training Program (PRTP)  Family Psychoeducational Training Program (FPTP) \| Personal recovery model according to Bedregal et al. (2006)  Family cognitive-behavioural psychoeducational treatment (Falloon, 1994) \| **PRTP:** Concepts of mental health outcomes, concepts and principles of the "personal recovery" process, and all group works targeting mental health and recovery knowledge/practice improvement, and empowerment recovery translation to practice  **FPTP:** The approach included the following strategies: individual evaluation of each member of the family, assessment of the communication skills and problem-solving capacity of the family as a whole, personal and family objective setting, education regarding the nature of the disorder and its bio- medical and psychosocial treatment, identification of early warning signs, improvement of communication skills, structured problem solving and social skills training \| Mental health professionals (psychiatrists, psychologists, nurses, and psychiatric rehabilitation technicians) and students of psychiatric rehabilitation techniques \| PRTP: leading mental health university and National Health System (NHS) experts. Two consumers were involved as teachers and tutors  FPTP: one of the Author R.R. \| PRTP: Lecture, discussion, and small/large groups work assisted by a tutor. \| PRTP: L’Aquila Psychiatric University Unit of the Department of Life, Health and Environmental Sciences, Italy  FPTP: L’Aquila (Italy) \| PRTP: lasting one day for 8 hours  FPTP: lasting 6 days (8 hours per day) \| N/R \| N/R \| \| --- \| --- \| --- \| --- \| --- \| --- \| --- \| --- \| --- \| --- \| --- \| --- \| | | | | | | | | | | | |
| **Multiple recovery theories** | | | | | | | | | | | |
| Felton et al. (2006) | USA | Core Assertive Community Treatment (ACT) | WRAP and IMR | A “recovery module” which introduced  1) models of recovery and described practices such as Deegan’s (1990) “key elements in supporting recovery.” 2) “recovery- centred service planning,” describing methods of enacting, through service plans, the recovery principles 3) WRAP planning (Copeland, 1997) | ACT team members of all disciplines, including psychiatrists, nurses, social workers, substance abuse specialists, family psychoeducation specialists, and peer counsellors | Nationally recognized experts in recovery and illness self-management and local peer and non- peer experts | Classroom-based training with face-to-face and presentation | The New York State ACT Institute | N/R | N/R | N/R |
| Tsai et al. (2010) | USA | General/inspirational training  Specific/practical skills training | SAMHSA recovery principles  IMR, WRAP, Integrated Dual Disorders Treatment, the Matrix model, motivational interviewing | **General/inspirational training**  - **Roadmap to Seclusion and Restraint Free Mental Health Settings:** 3-day workshop developed by SAMHSA which engages staff to think about their practices - **‘‘Comfort room’’ workgroups:** staff are planning rooms to provide a calming environment for clients to relieve stress, which have been proposed to reduce the use of restraints and seclusion - ‘**Bridge building’’:** de-escalation techniques and emphasizes use of the least restrictive methods necessary - **Respect seminars:** 1-day presentation by a private consultant and former consumer, Joel Slack, who is a well- known speaker on recovery  **Specific/practical skills training**  - **Illness Management and Recovery:** a curriculum-based treatment approach focused on teaching consumers how to set and achieve personal recovery goals, acquire knowledge, and use skills to independently manage their illnesses - **Integrated Dual Disorders Treatment:** teaches staff to provide mental health and substance abuse interventions together based on clients’ stage of treatment and readiness to change - **Wellness Recovery and Action Planning:** teaches staff to engage clients in their own care and personal goals by helping them develop specific recovery plans - **The Matrix model:** a structured treatment approach for substance abuse that staff can use to provide information and relapse prevention techniques - **Motivational interviewing:** teaches staff how to use their clients’ motivations and resources to change their behaviour | Supervisor/department supervisor Nurse Behavioural clinician/psychologist social worker | A private consultant and former consumer, Joel Slack, who is a well- known speaker on recovery | General/inspirational training: workshop and presentation  Specific/practical skills training: N/R | N/R | General/inspirational training: 3-day workshop (with 1 day presentation)  Specific/practical skills training: N/R | N/R | N/R |
| Hornik‐Lurie et. al. (2018) | Israel | Recovery‐oriented training interventions | IMR, peer-support, and psychiatric advance directives | **1) Illness management and recovery training:**  A psychoeducational, evidence‐based intervention, which supports individuals in developing the requisite knowledge and tools for optimal self‐management of their mental illness and personal goals, according to one’s vision of their own recovery.  Staff are trained to develop personal relationships and to focus on positive aspects, creating trust and hope about the ultimate attainment of personal goals.  **2) Work with Peer support within the team:**  Ten peer support workers and a peer supervisor were introduced into the Beer‐Sheva Mental Health Centre in 2015, part of a special demonstrative project supported by the National Insurance Institute of Israel and Ministry of Health.  After initial training, peer support workers receive ongoing supervision. They work in different wards across the hospital, participate in multidisciplinary team activities and provide individual and group interventions.  **3) A psychiatric advance directive training:**  To enables individuals receiving psychiatric care to declare, in advance, their preferences and directives (e.g., type of service, medication, key contacts) in the event of a mental crisis and subsequent loss of capacity to exercise independent judgement.  To protect the self‐dignity of the patient and safeguard one’s preferred treatment of choice  To encourage proactive patient participation in their own treatment and well‐being | Social workers, psychologists, psychiatric nurses, occupational therapists, psychiatrists | N/R | Individual and/or groups sessions | Beer‐Sheva Mental Health Centre, Ministry of Health, Israel | 1 hour individual and/or groups sessions per week are conducted over periods ranging from one to 11 months. | N/R | N/R |
| **Other recovery-oriented training** | | | | | | | | | | | |
| Daley et al. (2020) | UK | The Older Adults Recovery Intervention (OARI) | Based on the findings from the previous qualitative work and existing recovery literature to develop a team-based staff level recovery intervention | **The intervention has three components; *(i) team recovery training, (ii) action planning and (iii) implementation support***  There is didactic teaching on each module topic, for example, what is recovery and what is recovery-oriented practice, as well as practical exercises whereby staff share relevant clinical experiences, and consider how the training content can be delivered within routine practice.  The action planning (one day) component follows the recovery training, and involves the development of a team recovery action plan with specific objectives.  Implementation support includes support to developing new pro-recovery team processes (such as service user-facing documentation, or service user involvement in care planning), reviewing the team recovery action plan or educational supervision at a team or individual level delivered by the OARI trainer up to six months post action planning day.  The specific implementation support differs between teams, and is agreed with each team individually at their action planning day. The OARI is delivered by a mental health professional with training experience along with a service user trainer.  There are three modules each lasting one day: (1) Promoting Recovery, (2) Maintaining Identity and (3) Enhancing Resilience. Each module is delivered sequentially, with homework from the previous module. There is didactic teaching on each module topic. | Staff within the clinical team (nurse, OT, psychiatrist, psychologist, social worker, support worker) | OARI trainers: 2 nurses, 2 service users | OARI trainers: 2 nurses, 2 service users | In the older people’s mental health services in South-East London in the UK | 3 modules each lasting 1 day (totally 3 days training) | N/R | 176 participants (71%) completed all training modules (3 days), 7 (11%) receiving part of it (1–2 days) |
| Walsh et al. (2017) | Ireland | Recovery-based training | Guided by the principles of adult education and uses group work, individual work, conversations, and reflective practice | The content of the workshop includes defining the concept of recovery, exploration of the recovery principles and how these recovery principles can be adopted into clinical practice. | Nurses, clinical nurse managers, clinical nurse specialists, psychologist, social worker, occupational therapist | Not reported who is the main intervention providers, but service users, family members and service providers are involved in the reflective session to share their lived experience | Reflective session | N/R | 4 hours training workshop | N/R | N/R |
| Nardella et al. (2021) | Australia | Recovery training programme  The Mental Health Passport (MHP) | N/R  WRAP, daily planner, recovery action steps, an activity & mood diary and appointment calendar, and a section ‘My Story’ that describes the person’s history in their own words | **Stage 1: Staff education in personal recovery-oriented practice**  Topics included therapeutic facilitation, motivational interviewing, initiating recovery conversations, the principles of recovery, and trauma- informed care. **Stage 2: Co-design of the mental health passport** A process of co-design with consumers and allied health and nursing staff to develop a consumer resource tool, the Mental Health Passport (MHP), founded on the principles of recovery-oriented practice and implemented within the Clinic’s existing service delivery model  The MHP is designed to empower consumers by giving them access to their mental health information.  **Stage 3: Implementation of recovery-oriented practice on the acute mental health units**  Staff were encouraged to provide care to con- summers admitted to the acute mental health unit using a recovery-oriented approach.  To support this change in practice, on admission consumers were allocated a staff member as their ‘recovery coach’.  The MHP was introduced to consumers once their condition had stabilized sufficiently for them to begin focusing on their recovery.  During their acute care admission, consumers were encouraged to complete the section outlining ‘My Story’ with the intention that this would be used as a resource to aid communication with clinician on an ongoing basis.  The nurse recovery coaches then encouraged consumers to review and use other resources included in the MHP based on their personal preferences. | Acute care-based mental health nurses | N/R | Face to face workshop: Utilising multimedia resources, group activities, and simulation exercises (a self-directed education pack provided for nurses who could not attend workshops) | A private mental health clinic in Melbourne, Australia: Mental health units caring for acute and sub-acute patients and older adults and adults with general psychiatric conditions ward. | Consisting of a series of four interactive 60-minute workshops run over a four-month period (*workshops were held between morning and afternoon shifts)*  Being held between morning and afternoon shift | Nurses who missed a workshop workshops (i.e. those rostered to night shift) were provided self-directed learning packs to complete with follow-up provided by education facilitators | 44 (77.2%) of the 57 nurses working on the acute mental health wards had completed all four workshops |
| Young et al. (2005) | USA | Consumer-led intervention, Staff Supporting Skills for Self-Help | Grounded in an emerging national movement of consumers with severe mental illness | **Scientific presentation on self-help:**  Assess clinicians’ previous support of self- help and empowerment. Present scientific material about recovery, rehabilitation, and self-help. Discuss ways to involve consumers in increasing self-help and mutual support. **Structured dialogues:**  Hold small groups with equal numbers of consumers and clinicians. Focus on barriers to self-help. Discuss hopelessness regarding severe and persistent mental illness and compare with experiences that create hope. Discuss factors that impede and promote recovery, how self-help complements traditional treatment, and resistance to self-help. **Rehabilitation readiness:**  Present clinicians with rehabilitation readiness concepts and skills to help consumers set goals and develop coping strategies. Present information about how clinicians can manage their own stressors, consumer demands, and the larger mental health system. **Strategies for independence:**  Focus on strategies for minimizing consumer dependence on mental health professionals. Discuss consumer responsibility for recovery, consumer and clinician behaviours that interfere with progress and reduce quality of life, and strategies to help clinicians tolerate their discomfort with consumers practicing new behaviours. **Professional skills supporting self-help:**  Use small groups and role-playing techniques. Focus on helping staff understand how to support self-help without being intrusive. Present theories about self-help success, the characteristics of common mutual support groups, and how traditional treatment can support consumers’ progress regarding self- help. **Detailing:**  Continue to meet as needed with clinicians. Provide individual advice, group presentations, and role-playing techniques for problems. | N/R | Two of the authors, Knight (vice-president for recovery, rehabilitation, and mutual support for Value Options Healthcare) and Vogel (executive director of Double Trouble in Recovery, a national self-help organization) | Didactic education, small group discussions, role- playing techniques, clinician-SU dialogues, individual advice | Five large community mental health provider organizations in two western states (Arizona and Colorado) | 5 group components delivered over a 1- year period & 16 hours meeting with staff at a various time during the study | N/R | N/R |
| Okamoto et al. (2018) | Japan | Experience-based program for understanding the concept of recovery | ACT and observational practice to understand recovery | **Day 1:**   ***Lecture Group work (90 min):*** Rethink how to understand families and their characteristics and how to offer family support. Use specific case examples to clarify emotional pain of the family and effects on recovery, establish a support hypothesis, and clarify goals.   ***Lecture (90 min):*** Learn about goals and specific content of ACT support at private medical facilities (complete internal type) including attitudes on recovery, outreach/community care, and ACT characteristics, history, elements, effects, and issues.  ***Survey report (30 min):*** Listen to presentations on the results of an interview survey regarding the thoughts of families who are users of ACT. **Day 2:**  ***Observational practice (540 min):*** Experience of a single day of ACT. After participating in a pre-visit team meeting, accompany staff on providing visiting support to users and receive feedback from staff after completing the practical work. **Day 3:**   ***Group work (120 min):*** Group work and question and answer session on experience presentations and clinical cases after completing the observational practice.  ***Lecture (90 min):*** Learn about coordination between hospitals and communities, the fundamentals and elements of community lifestyle support (case management, recovery and strengths, outreach, multidisciplinary teams, social inclusion), mental health care centre support systems, public facility (network type) ACT implementation support goals, and the details and issues associated with specific support methods. | Nurses | N/R | Lectures, assertive community treatment (ACT) staff visits for recovery orientation, and group work | Psychiatric wards in Okayama City | 3 days training including 5 h of lectures, 9 h of ACT observational practice, and 2 h of group work | N/R | These 12 nurses participated in this program from the introductory lecture to the concluding observational practice on days 1 and 2. However, three did not participate in the group work from day 3 onward. Eventually, nine participants who completed the entire program were enrolled. |
| Deane et al. (2014) | Australia | Skills acquisition coaching and Transformational coaching (after CRM training in first two days) | Life Journey Enhancement Tools (LifeJET) | **Skills condition coaches** were trained to address problems with the implementation of the CRM that coachees brought to the session. Skills condition then focused on identifying and exploring solutions to organisational and personal barriers to implementation of the CRM with their clients. This could include clinical relationship issues, change enhancement strategies or the use of the LifeJET protocols for values clarification, goal setting or action planning **Transformational coaches** were trained to use the LifeJET protocols to assist in the coachees’ personal and/or professional development by continuing to explore their personal values, vision and important goals. Transformational coaching paralleled the coaching-style approach participants were using with clients. | N/R | Senior practitioners who were experienced in the CRM and were trained in the use of the GROW model: Goals—setting goals for each coaching session; Reality— exploring the coachees’ current situation; Options—examining their options; and Wrap-Up—evaluating options, creating a plan, and problem solving any foreseen difficulties | (i) Skills acquisition coaching generally focuses on the goals of the employer, using a didactic approach to teach the coachee new skills and techniques to improve their work performance (ii) Transformational coaching explores the coachee’s personal values and goals to promote personal growth and/or professional development | Four community-managed mental health organisations, representing 13 sites across four states of Australia | All staff received standard CRM training for the first 2 days following by 1 day of training specific to each condition (Skills vs transformational coaching)  12 months coaching  (1 hour coaching sessions once per month for 12 months) | N/R | N/R |

**Note:**

ACT = Assertive Community Treatment, CRM = The Collaborative Recovery model, CRTP = Collaborative Recovery Training Programme, IMR = Illness Management and Recovery, N/R = not reported, ROP = recovery-oriented practice, SAMHSA = Substance Abuse and Mental Health Services Administration, WRAP = Wellness Recovery Action Plans
