# Supplementary material for: Recovery-oriented mental health training interventions: An integrative review
Source: Int J Nurs Stud Adv. 2026 Feb 15;10:100510. doi: 10.1016/j.ijnsa.2026.100510 (PMC13080650; doi:10.1016/j.ijnsa.2026.100510)
Supplement: Supplementary file 4 [file mmc4.docx]

**Supplementary material file 4: Kirkpatrick’s model**

Summary of recovery-oriented training programmes according to the Kirkpatrick’s model (n = 30)

| **Study** | **ROTP** | **Four levels of Kirkpatrick’s model** | | | | | |
| --- | --- | --- | --- | --- | --- | --- | --- |
|  |  | **Reaction** | **Learning** | **Behaviour** | **Results** | | |
|  |  |  | **Staff outcome** |  | **Patient outcome** | **Service/organisation outcome** | **Intervention outcome** |
| Oades et al. (2005) | CRTP | N/R | N/R | N/R | N/R | N/R | N/R |
| Crowe et al. (2006) | CRTP | N/R | Staff attitudes and hopefulness improved after training. Trainees significantly increased their knowledge regarding principles of recovery and belief in the effectiveness of collaboration and consumer autonomy support, motivation enhancement, needs assessment, goal striving, and homework use.  No significant differences in pretraining scores between the two groups (government and nongovernment employees) on the knowledge scale, STARS, and the first factor of RAQ-7. However, on the second factor of RAQ-7, ratings were significantly higher for nongovernment employees than for government employees (t = –3.05, do = 246, p < .01).  Correlational analysis of post training ratings of the collaborative recovery knowledge scale with attitudes as reflected by STARS and the two factors of RAQ-7 indicated that the collaborative recovery knowledge scale is positively associated with recovery attitudes (first factor of RAQ-7, r = .41; second factor of RAQ-7, r = .39; and STARS, r = .13; p < .05 for all). | N/R | N/R | N/R | N/R |
| Salgado et al. (2010) | CRTP | N/R | Training improved providers’ recovery knowledge, attitudes, hopefulness, and optimism. Providers with both high and low dispositional hope (dividing pre-training HS scores at the 50th percentile) achieved similar gains.  The MANOVA (2 (pre/post training) x 2 (high/low hope)) indicated a main effect across both measures (the STARS and TOS) showing that recovery attitudes and optimism significantly improved over the course of training, F (2, 72) =58.10, p < .001, Z^2^ = .617.  A Wilcoxon Signed Ranks test showed significant improvements of RAQ scores from pre-test (MN 1= 4.29) to post- test (MSDN = 4.43), z = -3.35, p<.001, d=.284. To further explore the potential of an interaction, a Mann Whitney test of pre-post scores for the low-hope versus the high-hope group was conducted. The low hope group achieved significantly lower scores than the high hope group at pre-test (low hope Mdn = 4.14 versus high hope Mdn = 4.43, U = 481.50, p <.01) and post- test (low hope Mdn = 4.36 versus high hope Mdn = 4.71, U = 473.00, p < .01). | N/R | N/R | N/R | N/R |
| Uppal et al. (2010) | CRTP | N/R | N/R | N/R | N/R | Approximately 37% of the trained clinicians participating in the study were found to be implementing training protocols in clinical practice.  In addition, the average time taken to implement the protocols was 5.6 months following training. | N/R |
| Williamson et al. (2023) | CRM | N/R | Attitude pre and post-test: z score 8.19, p < 0.001  Skill pre and post-test: z score 6.89, p < 0.001  Knowledge was measured by using 10 true-or-false items (score range 0–10, with higher scores indicating greater knowledge): pre and post-test z score 8.44, p < 0.001  The staff development program significantly (p<0.001) improved self-rated knowledge, attitudes, and skills in applying CRM. At booster training, improvements in attitudes and self- confidence in implementing CRM were maintained. | The language used analysis in staff about definitions of recovery pre- and post-training, in total, 7,790 words from 613 definitions of recovery (pre- training = 376, post training = 237) were entered. The highest frequency of use for a given word was 178 uses, and the lowest was 15. Use of medicalized language was reduced by > 50%; the frequency of the word “illness” decreased from 55 to 19 uses, and “symptoms” from 25 to 10 uses. Recovery language showed a corresponding threefold increase in frequency, as indicated by “wellbeing” increasing from 25 to 75 uses and “resilience” from 16 to 56 uses.  Perceived importance of CRM implementation, confidence in implementing CRM, and confidence in the organization were measured with one item each on a visual analogue scale (range 0%–100%, with higher scores indicating greater importance or confidence):  - Important pre and post-test z score 1.87, p = 0.062  - Self-confidence pre and post-test z score 4.82, p < 0.01  - Organizational confidence pre and post-test z score 1.07, p = 0.286 | N/R | Ratings of the importance of CRM and confidence in the organization’s implementation did not change. Definitions of recovery illustrated development of shared language throughout the large mental health program. | N/R |
| Slade et al. (2015a) | REFOCUS | N/A | N/A | N/A | N/A | N/A | N/A |
| Slade et al. (2015b) | REFOCUS | N/R | N/R | Conversations between staff and patients about values, treatment preferences, and strengths might translate over time into changes in functioning and assessed need. The differences we found in this study between groups do not seem to have been mediated through changes in the recovery variables studied, which suggests a complex relation between these variables.  Recovery Practice Scale (RPS):  Skills: Intervention group = 2·79 (0·64); control group = 2·73 (0·66)  Behavioural intent: Intervention group = 1·66 (0·34); control group = 1·68 (0·37)  Behaviour: Intervention group = 1·78 (0·78); control group = 1·74 (0·77) | In 27 community-based adult mental health teams in two NHS Trusts in England, we found no significant effect of the REFOCUS intervention on recovery in patients with psychosis compared with usual treatment.  Most secondary outcomes (hope, quality of life, empowerment, wellbeing) did not differ, with the exceptions of improved functioning (which remained significant after adjustment for multiple testing) and staff-rated unmet needs (which became non-significant after adjustment) in the REFOCUS group.  High team participation was associated with higher staff-rated scores for recovery-promotion behaviour change (adjusted difference –0·4, 95% CI –0·7 to –0·2, p=0·001) and patient-rated QPR interpersonal scores (–1·6, –2·7 to –0·5, p=0·005) at follow-up than low participation. | The cost difference between the REFOCUS and control groups was £1062 (95% CI –1103 to 3017) in favour of the intervention, but the difference was not significant. Service costs were on average £657 less for patients receiving care from high-participation teams in the REFOCUS group than those for patients receiving care from low-participation teams in the REFOCUS group (95% CI –1555 to 4783), but this difference was also not significant. | N/A |
| Wallace et al. (2016) | REFOCUS | N/R | N/R | N/R | Participants reported that the intervention supported the development of an open and collaborative relationship with staff, with new conversations around values, strengths and goals. This was experienced as hope-inspiring and empowering.  **Category (service users' experiences of intervention)** **1. Pro-recovery tasks and activities**   **- Understanding values and treatment preferences** Being able to have conversation in some specific topics (sexuality and spirituality), participants feel that staff were ‘genuinely’ interested in them and wanted to get to know them as a person. Participants felt better understood, which helped to strengthen the working relationship.   **- Assessing strengths** Participants reported having a greater awareness of their strengths following these discussions e.g. resilience and kindness   **- Supporting goal-striving** Participants found it particularly useful when staff worked collaboratively with them on their goals, breaking them down into manageable steps, helping with motivation and identifying possible opportunities to aid goal-striving.   **- Reservations about the working practices** The recovery activities and resources were not always positively experienced, particularly where they were delivered in a formulaic and generic way.  **- Partnership projects** The partnership projects gave opportunities for social interaction with other service users and staff leading to new or stronger connections with others. **2. The working relationship**   **- Recovery-supporting changes in the relationship**  Participants have permission to discuss new topics, often neglected within traditional problem-focused conversations. This supported the development of a relationship in which service users felt staffs were genuinely interested in getting to know them as individuals.  **- Pre-existing recovery-supporting relationships**  In general, participants valued staff being supportive, ‘genuinely’ caring, open and honest in a constructive but not dismissive way.  **- Lack of noticeable change in the relationship** Some participants reported that their relationships with staff did not become more recovery-orientated during the REFOCUS intervention because decision-making power remaining with staff **3. Impact of the pro-recovery intervention**  **- Empowerment** Individuals felt empowered by the intervention in relation to both their mental health and other areas of life. Being given increased independence and choice in their care indicated that staff believed they were capable of managing increased responsibility. This in turn made individuals feel more confident in their ability to cope.   **- Identity** REFOCUS intervention facilitated greater self-awareness, prompting thoughts about a wide range of life areas that they otherwise rarely focused on, including their goals and values. Participants have a more positive self-image.   **- Hope and optimism** Staff members were encouraged to actively communicate their hope and belief in the person. Hope was seen by many participants as essential to recovery-promoting efforts, underpinning actions such as goal-striving or building relationships. Therefore, increasing hope was felt to be very powerful. | N/R | N/R |
| Clarke et al. (2020) | REFOCUS | Staff valued coaching training and used coaching skills to have tough as well as empowering, motivational conversations with service users.  They were positive about the resources within the ‘working practices’ intervention component.  The majority of staff were very receptive to the idea of incorporating the coaching skills and approach within their clinical practice and found the training prepared them for having tough as well as motivating, empowering conversations with service users.  Staff found the concept of three styles demonstrated in training (mentoring, directing and coaching) a helpful way of thinking about interactions.  Being confused between the different types of sessions and generally there was a mixed response to them. Some staff described the personal recovery training as thought-provoking and used it to reflect upon their own personal and team recovery-oriented values and practice. Not everyone agreed that the less structured reflection sessions, were an effective use of time. | N/R | Staff who successfully used the Values and Treatment Preferences (VTP) guide with their service users shared their success and became powerful advocates for the approach within their team. It gave staff the confidence and permission to initiate new conversations, particularly around sexuality and spirituality, which they felt had previously been off limits.  Participants felt that using the intervention helped refresh long-term clinician-service user relationships and helped them develop their knowledge and understanding of service users’ lives though noticing other, positive aspects of their individual identity. It helped some staff strike the difficult balance in clinical practice between knowing when to take a step back and allow a service user to manage a task or situation alone and when to step in and support. | N/R | The whole team training and reflection sessions helped create team cultures, structures and processes which were conducive to supporting recovery practice.  Providing reflection sessions and recovery training to the whole clinical team was viewed very favourably. They reported it improved team relationships and dynamics by providing precious, dedicated time for everyone to be a team and created a safe place for sharing work pressures and challenging one another’s practice.  The intervention also provided a space for problem-solving how to overcome obstacles to recovery practice and developing team plans for taking the recovery agenda forward. They reported the intervention had enabled them to have the time and space to identify many obstacles that needed to be overcome when working within ethical and legal frameworks, statutory requirements and within a complex organizational system.  In particular, they recognized the conflict that can be present between an individual’s goals, organizational goals and statutory responsibilities and the need to discuss strategies for marrying these different expectations and requirements. | The intervention reminded some clinicians to broaden their focus beyond a medical model-driven style of questioning and revisit decisions where they may have been risk-averse and over-protective. It also led some staff to revise their belief that no one recovers from severe mental illness and to question their own assumptions about their service user motivations, capabilities, goals, aspirations, strengths and personal attributes. |
| Leamy et al. (2014) | REFOCUS | **Individual practitioner readiness**: Some individuals expressed varying degrees of resentment and frustration that their team was required by their Trust to participate in the trial. At an individual level, people were able to refuse to consent to participate in the trial but may still have felt undue pressure and the situation certainly created mixed messages.  It was very common for clinicians to report that they felt they were already working in a recovery-oriented way and that the intervention did not offer them anything new.  Workers with less experience in the mental health field also reported benefits.  **Team readiness:**  Some team leaders and psychiatrists saw the REFOCUS intervention as an opportunity to establish the team’s identity and credentials as a Recovery team, and/or provide the vehicle to enhance team working. Others saw it as an extra burden, a threat to their professional identity, or resented it as an implied criticism of existing practice. At times, senior clinicians and team leaders actively blocked their team’s efforts to become more recovery focussed.  Participants reported a lack of time for reading the intervention manual, reflection, practicing new skills, using the individual recovery supervision guide, and embedding the intervention into their existing practice with all their clients. | N/R | Some clinicians were able to quickly absorb the skills training and focus upon considering how and when to incorporate the approach into their routine practice. | N/R | Given the resource constraints, several clinicians felt that certain tasks within the intervention did not fall within their remit and should be carried out by care co-ordinators or support workers employed on lower pay grades. | N/R |
| Wilrycx et al. (2012) | ROTP | N/R | This study shows that professionals’ attitudes towards recovery from mental illness can improve with training. After two intensive recovery-oriented training sessions, mental health care professionals have a more positive attitude towards recovery in clinical practice.  RAQ:  For the RAQ, the null hypothesis that there were no systematic differences between the means of the two subsamples could not be rejected with a χ2 = 0.890 with 3 degrees of freedom (P = 0.828).  Intervention A has a significant effect since the null hypothesis μA = μO has to be rejected with a χ2 = 8.097 with 1 degree of freedom (P = 0.004). Also, the null hypothesis μB = μO has to be rejected (χ2 = 29.603, df = 1, P = 0.000), indicating that intervention B has an effect. Finally, also the hypothesis μB = μA is rejected (χ2 = 5.783, df = 2, P = 0.016), and intervention B is seen to have a larger effect than intervention A.  RKI:  For the RKI, the null hypothesis that there were no systematic differences between the means of the two subsamples could not be rejected with a χ2 = 1.641 with 3 degrees of freedom (P = 0.650).  Intervention A has a significant effect since the null hypothesis μA = μO has to be rejected with a χ2 = 17.888 with 1 degree of freedom (P = 0.000). However, the null hypothesis μB = μO cannot be rejected (χ2 = 2.939, df = 1, P = 0.086), and intervention B fails to have an effect. | N/R | N/R | N/R | N/R |
| Wilrycx et al. (2015) | ROTP | N/R | N/R | N/R | Scores showed a significant change over time for the subscale ‘Learning & new potentials’ of the MHRM. Significant effects were also found for gender, with men scoring higher than women on the subscales ‘Self-empowerment’ and ‘Learning & new potentials.’  The hypothesis of no mean change over time could only be rejected for one scale: the second subscale of the Mental Health Recovery Measure (MHRM), that is, Learning & new potentials. This was confirmed by a significance test of the hypothesis that only the means over the first five time points remained constant, while the mean at T = 6 was left unconstrained. This hypothesis could not be rejected: χ2 = 1.914 for df = 4 and p = .752. | N/R | N/R |
| Zuaboni et al. (2017) | ROTNP | N/R | N/R | N/R | N/R | No statistically significant effects were found, between the intervention ward and control ward in regard to both patients and MHNs outcomes.  The German version of the RSA Scale (RSA-D) (provider scale): No significant differences could be observed when comparing the outcomes of the intervention wards and the control wards. | N/R |
| Meadows et al. (2019) | REFOCUS-PULSAR | N/R | N/R | N/R | The mean Process of Recovery (QPR) score was 53·6 (SD 16·3) in the control group and 54·4 (16·2) in the intervention group (adjusted difference 3∙7, 95% CI 0·5–6·8; p=0·023). The Cohen’s d value for the intervention effect was small (d=0·23).  QPR scores improved from before to after intervention delivery for consumers of public mental health services in the step-two group (mean difference 4·9; Z score=3·0; p=0·003; d=0·30) and for consumers of mental health community support services in the step- one group (1·1; Z score=2·7; p=0·006; d=0·07)  **INSPIRE and the Perceived Need for Care Questionnaire:**  None of the outcomes were significantly different between the intervention and control groups. | N/R | N/R |
| Edan et al. (2019) | REFOCUS-PULSAR | N/R | N/R | Support for recovery as a practice: for staff who already felt that the principles of ROP were the ‘cornerstone’ of their practice, striving to practice in a way that supports dignity of risk and having different kinds of conversations with consumers were both important and rewarding elements of working with consumers in a recovery-oriented way. | For consumers, being on a CTO meant lacking choice and control, an emphasis on medication, fear of the threat of hospitalisation, an absence of recovery-oriented practice, and staying supported.  - Lack of choice and control: being on CTOs makes consumers feeling lack of choice and control and being experienced as burdensome. This lack of control contributed not only to a reliance on services or other people, but also to a sense of being imposed upon by services.  - Absence of recovery-oriented practice: most consumer participants had not had a conversation with clinical staff that they could identify as recovery focussed | N/R | **Put in the Barriers:** For staff, recovery-oriented practice in the presence of CTOs is challenging, with CTOs being seen to be a primary way to manage risk.  Staff participants reported the importance of recovery in their practice and believed that the PULSAR training augmented their knowledge in the area, with one staff participant indicating that the training had strengthened a move towards co-design in their service. |
| Enticott et al. (2021) | REFOCUS-PULSAR | N/R | N/R | N/R | These exploratory findings suggest better patient outcomes followed introducing GPs to ROP in routine practice conditions.  Small positive significant effects indicated primary-outcome (QPR) post-intervention improvements [t-test (233) = −2.23, p = 0.01], also improvement in two secondary outcomes (WEMWBS t (233) = −2.12, p = 0.02 and K10 t (233) = 2.44, p = 0.01). | N/R | N/R |
| Kehoe et al. (2023) | REFOCUS-PULSAR | N/R | N/R | N/R | **‘Recovery needs connection’:** connection can come through relationships. Those in residential settings focused on forming connections within the group setting whilst looking ahead to being a part of the broader community and building connections in the future. For those receiving treatment in the community, a connection to others within the community was highly valued as supporting their recovery. **‘a better life’:** For many consumers, the process of recovery was how they made meaning of their experience, which included aspects such as developing a sense of purpose, fostering hope and a new future, or developing relationships that can support their wellbeing through the recovery journey  For some participants there was an overarching theme around a strong sense of uncertainty for the future. This expression of uncertainty, doubt, and “fear” suggested that participants’ sense of recovery and what their future would look like was unclear to them.  Throughout this study, it was evident that only a minority of consumers could identify particular ROP language, for example, working on a strength; the vast majority being unable to recall any aspects of recovery despite being prompted about their personal experiences. | N/R | N/R |
| Repique et al.(2016) | SAMHSA | Question 1: the clarity of the material and the strength  Participants (in focus group) described the clarity and strength of the material presented in the training program as “good.”  The recovery principles were presented well but participants felt that they were basic concepts that they already knew, and the content of the material was nothing new for them.  The participants would have preferred a different recovery-oriented training program more tailored and applicable to their practice in their respective inpatient settings.  The material presented was “not specific enough.”  The participants would have liked to learn more regarding “specific examples” of how recovery principles are actually put into practice in an acute care psychiatric hospital. | **The Recovery Knowledge Inventory (RKI)**  **Result:** To examine participants' change in knowledge about recovery from pre to post intervention, a series of independent samples t tests were conducted on each of the four RKI domains and no significant difference between pre and post was found for RKI domain scores (all ps > .05).  Findings suggest that although there was no significant difference in the results from the pre and post RKI surveys, recovery-oriented training programs for PMH-RNs can be a potentially useful hospital strategy for restraint reduction. | N/R | Restrain rate  - During the pre-intervention phase (Q1 and Q2 of 2015), restrain rate 1.48 episodes/1000 patients  - During the intervention period (Q3) restrain rate 2.23/1000 patients  - At the completion of the post-intervention phase (end of Q4) restrain rate 2.29/1000 patients  This showed a slight reduction in restraint rates three months after post-intervention | N/R | If the training program is delivered via a webinar in the future, they suggested supplementing the presentation by having a content expert on-site that knows the training material to respond to any questions or need for clarification.  The participants would have preferred a training program delivered by a live presenter instead of a webinar format so it could have been more interactive. |
| Mak et al. (2019) | BRPP | N/R | Participants in psychoeducation group had significantly better recovery knowledge and more positive attitudes towards recovery after the intervention than the control counterparts. The effect of the recovery psychoeducation program on recovery attitudes was fully mediated by the improvement in recovery knowledge.  RKI:  Result: Participants who received the psychoeducation program showed a significant increase in recovery-oriented knowledge (∆ = 0.42, p < 0.001, Cohen’s d = 1.06) whereas control group participants did not have any changes in recovery-oriented knowledge between the two time points (∆ = 0.01, p > 0.05, Cohen’s d = 0.03).  ARQ:  Result: The participants in intervention group had a significant improvement in recovery attitudes (∆ = 0.14, p = 0.001, Cohen’s d = 0.38). No significant changes on recovery attitudes were observed in the participants in control group (∆ = − 0.04, p > .05, Cohen’s d = 0.11). | N/R | Participants in psychoeducation group had significantly better recovery knowledge and more positive attitudes towards recovery after the intervention than the control counterparts. The effect of the recovery psychoeducation program on recovery attitudes was fully mediated by the improvement in recovery knowledge.  RKI:  Result: participants in psychoeducation group had a significantly higher level of recovery-oriented knowledge after the program than at baseline (∆ = 0.20, p < 0.001, Cohen’s d = 0.55), but no significant difference between scores at baseline and one-month follow-up was found (∆ = 0.05, p > 0.05, Cohen’s d = 0.13).  ARQ:  Result: No significant time differences on recovery attitudes from baseline to post-program were found in psychoeducation group (p > 0.05). The results also showed non-significant changes between scores at baseline and one-month follow-up in the two groups (ps > 0.05). | N/R | N/R |
| Tsai et al. (2011) | RRT | N/R | Compared to staff who had no recovery-related training in the past year, staff who had at least one recovery-related training reported significantly higher consumer optimism and a greater agency recovery orientation towards consumers’ life goals. The number of recovery-related trainings was significantly correlated with scores on personal optimism, consumer optimism, and agency recovery orientation towards consumers’ life goals.  The Recovery Self-Assessment (RSA) (an overall scale alpha of .92): from assessing recovery attitudes of staff across sites found significant differences between sites on the Choice factor in Agency Recovery Orientation (F= 5.24, p<.001) | N/R | The Consumer Optimism scale consists of 16 items (alpha= .91): from assessing recovery attitudes of consumers by staff across sites found significant differences between sites on Consumer Optimism (F= 4.99, p<.001). | N/R | N/R |
| Felton et al. (2006) | ACT | Trainees’ disbelief about the appropriateness of recovery for certain recipients and misunderstandings of it e.g. they do not belief this training is appropriate for people with substance abuse | N/R | N/R | N/R | N/R | N/R |
| Tsai et al. (2010) | GIT + SPST | N/R | Staff who received specific/practical training had a greater increase in agency recovery attitudes than staff who received only general/inspirational training or no training. However, the more trainings staff had, the higher their consumer optimism. | N/R | **The Consumer Optimism scale** consists of 16 items; alpha =0.91 (staff-rated questionnaires): no significant differences between training types | N/R | N/R |
| Hornik‐Lurie et. al. (2018) | ROTP | N/R | The quantitative outcomes partially confirmed positive changes in attitudes and some practices.  RAQ: There were statistically significant differences, at the one‐tail 0.05 level of significance, between the study group (staff trained in recovery‐oriented intervention) and the comparison group (not trained).  RKI: Statistically significant differences were also recorded by three of the four subscales: increased attribution of roles and responsibilities to patients; nonlinearity of recovery processes; and the roles of self‐definition and peers in recovery.  RSA-R: There were statistically significant differences between the two study groups: in three of six factors: consumer involvement, diversity of treatment options and client choice. No statistically significant differences were recorded in the other three subscales.  Main findings from qualitative data  1.1) Illness management and recovery benefits:  Increased interest and understanding of patients: (1) Change in attitudes, (2) Greater appreciation, (3) More person‐focused  Increased emphasis on person‐centred practice: (1) Enhanced emphasis on capabilities, (2) Enhanced emphasis on autonomy, (3) More emphasis on hope  1.2) Illness management and recovery challenges  Need for more training and supervision: (1) Lack of recovery‐oriented interventions training for all dept. personnel, (2) Need for ongoing supervision on recovery‐oriented interventions.  Lack of continuity post discharge: (1) Lack of connection with post hospitalization services to individually tailor community services  Difficulty implementing recovery‐oriented interventions in acute states: (1) Conflicting values of choice and care, (2) Limited time and resources, (3) Doubts about partnering with patients | N/R | N/R | N/R | N/R |
| Daley et al. (2020) | OARI | N/R | There was a statistically significant change towards improvement in four of the six recovery attitude and knowledge sub-scales. There were positive findings in change in practice at individual level, but not at team level.  **RKI & RAQ-7** There was positive changes in all scales, with statistically significant change towards improvement in four of the six subscales: RAQ-7 sub-scale 1 (Recovery is possible), and RKI sub-scales 1 (Roles), 2 (non-linearity) and 3 (Self-definition). | N/R | N/R | N/R | First the scope of OARI was very broad and sought to address recovery-oriented practice in relation to a wide range of service users. This may have made the specific practice implications insufficiently defined for staff.  Whilst the training component of the intervention was delivered successfully overall, fidelity was more problematic. OARI was developed as a team-based intervention and not all team members participated. Implementation support was also patchy. Lack of implementation support was identified by staff as reducing the team focus on recovery.  There was a lower level of reach to psychiatrists. The consequent impact of psychiatrists not engaging in the intervention was identified in the qualitative interviews by non-psychiatrists as a factor which undermined the implementation of recovery-practice within teams. This is consistent with other studies of recovery training, where lack of engagement by psychiatrists in recovery training has acted as a barrier to subsequent practice change.  The lack of available evidence supporting about recovery for users of older people’s mental services affected the acceptability of the intervention. |
| Giusti et al. (2022) | PRTP + FPTP | N/R | Participants’ understanding of personal recovery improved more significantly for those in the PRTP than for those in the FPTP group in two domains, “Roles and responsibilities” and “non-linearity of the recovery process”; the FPTP group showed a significant improvement in the “Role of self-definition and peers in recovery” domain.   The findings indicate that a brief PRTP supported by consumers can improve staff and students' recovery orientation.  RKI:  RKI total score, increased significantly more among those undertaking the PRTP than among those in the FPTP group [F (1,90), = 7.39; p < 0.001]. | N/R | N/R | N/R | N/R |
| Walsh et al. (2017) | RBT | N/R | The results indicate a significant difference in confidence using a recovery model of care following training suggesting that recovery-based training positively affects staff knowledge and attitudes to recovery overall.  RKI: All domains showed significant changes between pre- and post-training scores (p < 0.01).  RAQ: Both (factor 1 and 2) factors showed significant changes between pre- and post- training scores in Factor 1 (p < 0.001) and in Factor 2 (p < 0.009). | N/R | N/R | N/R | N/R |
| Nardella et al. (2021) | RTP + MHP | N/R | This study found that following completion of the staff education programme, participants had a good understanding of the ‘Roles and responsibilities in recovery’ and this was still evident amongst the inpatient nursing team 12 months later. There was also an improvement in nurses’ responses on the ‘Expectation regarding recovery’ subscale at 12 months. These findings indicate that some of the understandings around recovery- orientated practice that nurses had gained from the education programme were sustained over time and were being integrated into usual practice at the study site.  RKI: Group 2 scores for the ‘Role of self-definition and peers in recovery’ subscale was lower than those reported by Group 1 (mean difference 0.391, P = 0.019). In contrast, Group 2 participants scored higher on the ‘Expectations regarding recovery’ subscale (mean difference +.636, P = 0.035) than Group 1 participants.  RSA-Provider: scores for the ‘Life Goals’ factor (Group 1 M = 3.28, SD = 0.78 versus Group 2M=3.72, SD=0.52, P=0.025) and the ‘Choice’ factor (Group 1 M = 3.21, SD = 1.05 versus Group 2 M = 3.94, SD = 0.85, P = 0.010) were higher at the second time point.  Emergent themes from focus group  Theme-1 Understanding of recovery-oriented practice.  The majority of nurses believed themselves to have knowledge about the concept of personal recovery-oriented practice, describing a recovery-oriented approach as being individual patient or client-centred and goals driven.  Theme-2 How to embed personal recovery- oriented care into clinical practice.  Most participants indicated that they had adequate knowledge of recovery-oriented care and several expressed that they did not see any changes in their own practice after the formal introduction of recovery- oriented practice on their unit. However, one participant commented that further culture change was needed to alter staff attitudes to consumer recovery. | N/R | N/R | N/R | N/R |
| Young et al. (2005) | CISSS | N/R | Compared with clinicians at the control organizations, clinicians at intervention organizations showed significantly greater improvement in education about care, rehabilitation methods, natural supports, holistic approaches, teamwork, overall competency, and recovery orientation.  The Competency Assessment Instrument (CAI):  - General competencies  - Assessment and treatment competencies  - Rehabilitation competencies Holistic approach | N/R | N/R | Preintervention interviews:  Interviews with managers and clinicians revealed substantial variation in the organizations’ understanding and use of recovery- oriented services.  Attitudes and behaviours that foster psychiatric rehabilitation and client-centred care were not well understood.  Staff at most sites reported that their organizations espoused a shift toward recovery-oriented services and that training toward this end would be beneficial.  1 year post training interview:  At one year, interviews revealed that intervention sites were providing more recovery-oriented services than control sites.  At intervention sites, clinicians reported more support from management for implementing new rehabilitation services.  They commonly stated that providing rehabilitation is hard work and takes a new kind of thoughtfulness. | N/R |
| Okamoto et al. (2018) | EBP | N/R | The mean Recovery Knowledge Inventory of nine participants ultimately included was 3.41 points (SD, 0.28) before the program and 3.69 points (SD, 0.24) after the program, indicating a significant difference (p = .004).  Mixed results:  RAQ: No significant difference was noted between the mean RAQ-7 score before and after the programme.  RKI: Significant differences were noted (p = .004)  Categories obtained from ACT support experiences included the following: “Continuing to attend to the need to live in one’s community/home regardless of how bad psychiatric symptoms become without the use of medicines,” “Viewing the person living their life in a place where they belong and in their own individual style,” “Valuing the patient’s wishes is the slow but sure way to a fruitful relationship,” and “Become familiar to the patient and their family’s life- style by carefully listening to the family’s feelings”. | N/R | Qualitative descriptive research using descriptions of observational practice:  Data were collected by asking participants to freely describe their thoughts on their experience of “further understanding recovery through experiencing ACT for one day” displayed as 4 categories as:  (i) Continuing to attend to the need to live in one’s community/home regardless of how bad psychiatric symptoms become without the use of medicines  - Even somebody with such bad symptoms as a result of non-medication can live in their community/at home by means of ACT  - Continuing to attend to whatever fundamental style or needs the person has to facilitate community life  (ii) Viewing the person living their life in a place where they belong and in their own individual style  - Continuing to believe in and to find out about the patient’s abilities so that they can live in a place where they belong in their own individual style  - Not being able to patiently respond to the patient at hospitals that offer medical and pharmaceutical treatment, focusing on the patient’s pathological condition alone  (III) Valuing the patient’s wishes is the slow but sure way to a fruitful relationship  - Value the patient’s wishes while building a steady relationship so that they can make a call for help whenever they need to  (Iv) Become familiar to the patient and their family’s lifestyle by carefully listening to the family’s feelings.  - Offering security and support by spending time close to the family so that the patient and their family could live together, keeping a certain amount of distance  - Reducing family stress by listening carefully to them and offering help when they are tired or weak] | N/R | N/R |
| Deane et al. (2014) | SAC + TC | N/R | N/R | N/R | N/R | Two days of the CRM training followed by coaching led to significant sustained improvements in the quality-of-care planning in accordance with the new model over the 12-month study period. No significant difference between the coaching conditions in the number of Coaching Record Sheets returned (p > 0.05). | N/R |

Note: CRTP = Collaborative Recovery Training Program, ROTP = Recovery-oriented Training Programme, ROTNP = Recovery-oriented Training Nursing Programme, BRPP = Brief Recovery Psychoeducation Programme, RRT = Recovery-related Training, ACT = Assertive Community Treatment, GIT = General/inspirational training, SPST = Specific/practical skills training, OARI = The Older Adults Recovery Intervention, PRTP = Personal Recovery Training Program, FPTP = Family Psychoeducational Training Program, RBT = Recovery-based Training, RTP = Recovery Training Programme, MHP = Mental Health Passport, CISSS = Consumer-led intervention, Staff Supporting Skills for Self-Help, EBP = Experience-based program for understanding the concept of recovery, SAC = Skills acquisition coaching, TC = Transformational Coaching, N/R = not reported
